# Insights into Carvone: Fatty Acid Hydrophobic NADES for Alkane Solubilization

**DOI:** 10.1021/acs.energyfuels.4c03623

**Published:** 2024-12-05

**Authors:** Nuria Aguilar, Cristina Benito, Sonia Martel-Martín, Alberto Gutiérrez, Sara Rozas, Pedro A. Marcos, Alfredo Bol-Arreba, Mert Atilhan, Santiago Aparicio

**Affiliations:** † Department of Chemistry, University of Burgos, 09001 Burgos, Spain; ‡ International Research Centre in Critical Raw Materials-ICCRAM, University of Burgos, 09001 Burgos, Spain; § Department of Physics, University of Burgos, 09001 Burgos, Spain; ∥ Department of Chemical and Paper Engineering, Western Michigan University, Kalamazoo, Michigan 49008-5462, United States

## Abstract

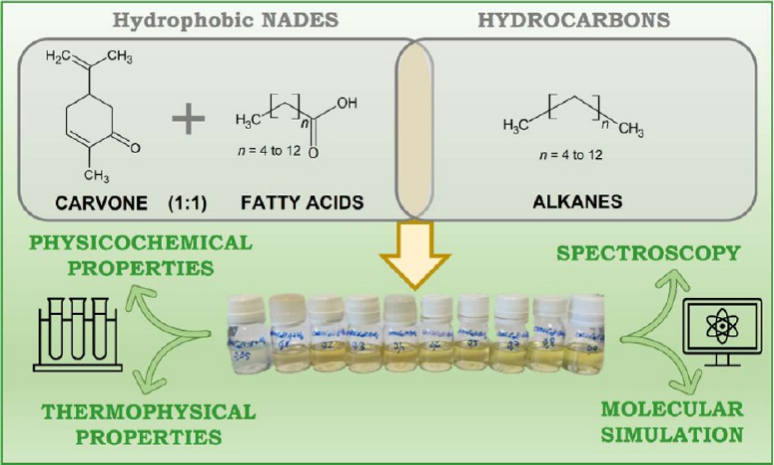

The urge to adopt cleaner technologies drives the search for novel and sustainable materials such as Hydrophobic Natural Deep Eutectic Solvents (HNADESs), a new class of green solvents characterized by their low toxicity, biodegradability, and tunable properties, aiming to be applied in various fields for handling non-polar substances. In this work, the solubilization of hydrocarbons in type V HNADESs (non-ionic organic molecules) formed by mixing carvone, a natural monoterpenoid, with organic acids (hexanoic to decanoic acids) is examined by applying both experimental and theoretical approaches. The synthesis and physicochemical characterization of different HNADESs allowed us to tailor their properties, aiming for optimal interactions with desired hydrocarbons. The solubilization of hydrocarbons in CAR:C10AC (1:1) HNADES is evaluated in terms of HNADES content, temperature, and the structure of the hydrocarbon itself (C6, C10, and C14 being the selected ones). To gain deeper insights into the underlying mechanisms of interactions between the solvents and the alkanes, a comprehensive multiscale computational study was carried out to analyze the nature of the interactions, the changes upon formation of HNADESs and hydrocarbon solubilization in the fluid’s nanostructure, and the possible toxicological effects of the solvents. The findings hold the potential to significantly impact the realm of hydrocarbon exploration and utilization.

## 1 Introduction

The ever-increasing demand for cleaner and more sustainable technologies has propelled the exploration of novel materials with tailored properties.^1^ In this context, deep eutectic solvents (DESs) have emerged as a promising class of tunable green solvents, offering unique advantages over conventional organic ones.^2^ DESs are formed by mixing two or more components, typically a hydrogen bond acceptor (HBA) and a hydrogen bond donor (HBD), characterized by a melting point depression, i.e., the melting point of the resulting mixture being lower than those of the individual components.^3,4^ This unique characteristic arises from strong intermolecular interactions that disrupt the crystal lattice, leading to a liquid state at room temperature.^5^ The remarkable diversity of HBAs and HBDs, along with their tunable properties, biodegradability,^6^ low toxicity,^7^ and low cost, has led to their diverse usages in various fields, including catalysis,^8^ material syntheses in polymer science, metal processing,^9^ natural products,^10^ separations,^11^ environmental remediation,^12^ pharmacology,^13^ and water treatment,^14,15^ to mention the most relevant applications.

DESs can be broadly classified into five families based on the nature of their components, with those belonging to types III (usually containing salts such as choline chloride as HBAs) and V (non-ionic organic components) being the most studied ones and with the most promising technological applications.^16^ Likewise, natural deep eutectic solvents (NADES) are developed considering biobased constituents like sugars, organic acids, and amino acids.^17,18^ NADES offer several advantages over conventional DESs, making them particularly attractive for sustainable applications. Their biobased components are readily available, renewable, and often exhibit low toxicity and biodegradability, minimizing environmental concerns. Additionally, NADES can be easily synthesized using simple methods and readily tailored for specific applications by manipulating the starting components and their ratios.^19−21^

Traditional DESs often exhibit hydrophilic character, but the development of hydrophobic DESs (HDESs) has opened new avenues for applications involving non-polar molecules.^22,23^ HDESs are typically synthesized using non-polar HBAs and HBDs, from which physicochemical properties like low water solubility and high affinity for organic compounds are derived.^24,25^ These advantages are enhanced if natural compounds are used, leading to hydrophobic natural deep eutectic solvents (HNADES). Therefore, these properties make them ideal candidates for various applications, including (i) extraction of hydrophobic pollutants from water: HDESs can selectively extract pollutants like polycyclic aromatic hydrocarbons (PAHs) or PFAS from aqueous environments, offering a green alternative to conventional methods;^26−29^ (ii) biocatalysis: HDESs can be used as reaction media for enzymatic reactions involving hydrophobic substrates, enhancing the solubility and activity of enzymes;^30−32^ and (iii) separation processes: HDESs can be utilized for selective separation of organic compounds based on their polarity, offering potential applications in purification and recycling.^33,34^

The unique properties of HNADESs make them promising candidates for various applications related to hydrocarbons. Some of the key areas include (i) extraction and processing of hydrocarbons: HNADESs can be used to extract hydrocarbons from unconventional sources such as shale formations or for selective separation of specific hydrocarbon fractions,^35,36^ (ii) upgrading heavy hydrocarbons: HNADESs can facilitate the demetalation, desulfurization, and other upgrading processes of heavy crude oils, leading to clean and more valuable products,^37−41^ and (iii) hydrocarbon storage and transportation: HNADESs with tailored properties could be used for storing and transporting hydrocarbons, offering advantages over traditional methods in terms of safety and efficiency. Therefore, this work explores the frontiers of HNADES technologies for hydrocarbon(s) solubilization, reporting a comprehensive investigation into the potential of HNADESs for solubilizing hydrocarbons. The considered study combines both experimental and theoretical approaches to (i) synthesize and characterize various HNADESs based on carvone (a natural monoterpenoid, CAR) and fatty acids (hexanoic, C6AC, to decanoic acid, C10AC) via different combinations to form HNADESs with diverse tailored properties, aiming optimal hydrocarbon solubilization. The solubility and liquid mixture behavior of various hydrocarbons in the synthesized HNADESs will be studied, analyzing the influence of factors like HNADES composition, temperature, and hydrocarbon structure. Likewise, a comprehensive theoretical study is carried out using molecular modeling techniques to analyze their fluid behavior at the nanoscopic level, thus allowing the development of predictive models for the interaction and properties of HNADES/hydrocarbons, providing insights into the underlying mechanisms of solubilization. Our findings will contribute to the growing body of knowledge about HNADES technologies for hydrocarbon extraction and solubilization.

## 2 Materials and Methods

### 2.1 Chemicals and HNADES Preparation

**Figure 1:**
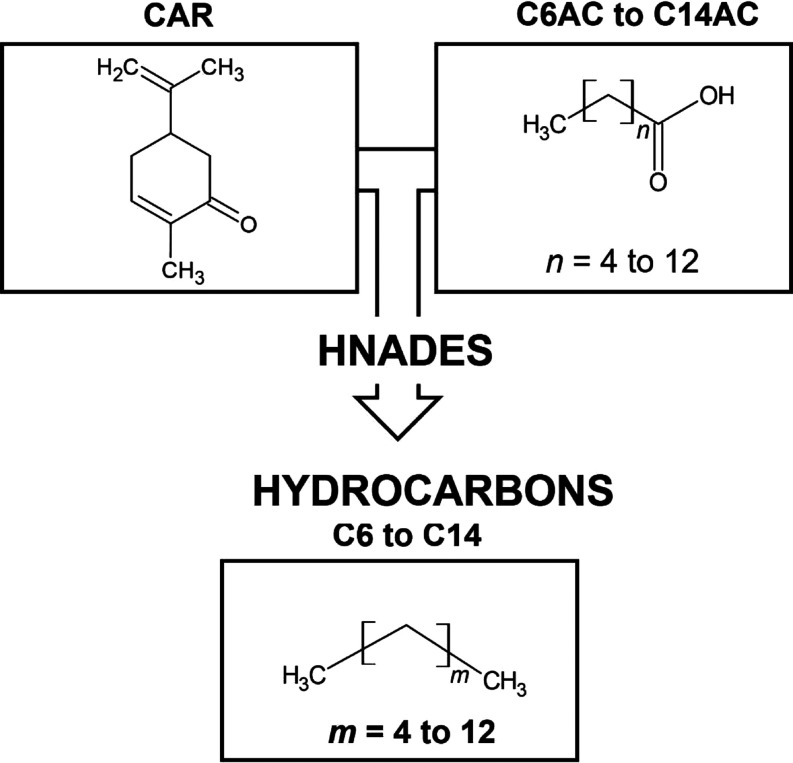
Molecular structures of compounds used in this work and the developed HNADESs.

HNADES components (CAR, C6AC, C8AC, C10AC, and C14AC) were purchased commercially, with purities listed in Table S1 (Supporting Information). As shown in Figure 1, HNADESs were prepared by precisely weighing (using a Mettler AT261 balance with ±1 × 10^–5^ g accuracy) the required amounts of HBA and HBD according to the desired molar ratios. The mixture was then stirred under heat (40 °C) in an inert atmosphere until a liquid phase was obtained. The liquid samples were dried using a Heidolph rotary evaporator to remove any residual solvents and water under vacuum at 40 °C. The resulting samples were cooled to room temperature (25 °C) and remained liquid for weeks without any solidification or turbidity. Notably, all of the HNADES prepared at different molar ratios were transparent and colorless. Melting temperatures for the prepared dried HNADES samples were measured using a home-built device with samples placed in glass vials into a thermostat bath with the temperature measured with a platinum resistance thermometer (PRT, ±0.01 K). For determining the transition temperature, cooling and heating steps were carried out for the samples, with solid formation detected through visual inspection of the sample (turbidity appearance or disappearance).

HNADES + hydrocarbon mixtures (Table S1 in the Supporting Information) were prepared as well by weighing the corresponding amounts of each component to cover the whole composition range.

### 2.2 Apparatus and Experimental Procedures

Density (ρ, uncertainty ±1 × 10^–4^ g cm^–3^) was measured using an Anton Paar DMA1001 vibrating tube densimeter, with the cell temperature controlled and measured by a built-in internal Peltier (uncertainty ±0.01 K). The temperature evolution of density followed a linear trend for all of the considered systems (pure HNADESs and HNADES + hydrocarbon mixtures with *R*^2^ > 0.9999 for linear fits of density vs temperature), from which the thermal expansion coefficients, α_p_, were calculated using eq 1
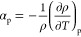
1

Dynamic viscosity (η) was measured with an electromagnetic VINCI Tech EV1000 viscometer^42^ (uncertainty 2%), coupled with a constant temperature circulating bath (Julabo Presto) for temperature control, with the temperature being measured in the cell with a platinum resistance thermometer (PRT, ±0.01 K). The measured η as a function of temperature did not follow Arrhenius behavior, and thus, they were fitted to the Vogel–Fulcher–Taman (VFT) equation (eq 2).
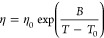
2

VFT fitting parameters were used to calculate Angell’s fragility parameter, *D*_f_, via eq 3.
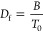
3

The refraction index (sodium *D*-line, *n*_D_) was measured using a Leica AR600 refractometer (±1 × 10^–5^), also coupled to an external circulator (Julabo F32) for temperature control, with the temperature measured with a PRT in the cell (±0.01 K). Thermal conductivity (σ) was measured with a Decagon devices KD2 Thermal analyzer (KS-1 sensor, 6 cm long, 1.3 mm diameter single needle, uncertainty 5%), and the temperature was controlled using a Julabo F32 bath and measured with a PRT (±0.01 K).

Raman spectra were collected using a StellarNet-HR-TEC-785 spectrometer (4 cm^–1^ resolution) for a 785 nm laser excitation wavelength, including an enhanced CCD array detector.

The hydrophilicity of the studied DES was studied by measuring the absorption rates of atmospheric water. These experiments were carried out by placing 15 cm^3^ of ethaline in a Petri dish, 90 mm in diameter, with 25.5 cm^2^ of liquid surface exposed to air. The water content was measured as a function of time by extracting aliquots (0.01 g) and measuring using a Karl Fischer coulometer. The atmospheric relative humidity during these experiments was 60 ± 5%. Water absorption kinetics was fitted to eq 4
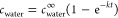
4where *c*_water_ stands for the water content (wt %), *c*_water_^∞^ is the limiting absorption value, and *k* is the kinetic constant.

Experimental properties are reported in Tables S2 and S3 (Supporting Information).

### 2.3 Molecular Modeling

#### 2.3.1 Density Functional Theory Calculations

DFT calculations for monomers, HBA–HBD dimers, and HBA–HBD–hydrocarbon complexes for different molecular orientations were carried out through geometrical optimizations at BP86/def2-TZVP plus Grimme’s D3^43^ theoretical level using Turbomole software.^44^ Binding energy, Δ*E*, for the different clusters was calculated as the difference between the energy of the corresponding cluster and the sum of the corresponding monomers. Topological analysis of intermolecular interactions was carried out by applying the quantum theory of atoms in molecules (Bader’s QTAIM theory)^45^ with MultiWFN software.^46^ The QTAIM analysis of intermolecular forces was carried out considering electron density (ρ_e_) and Laplacian of the electron density (∇^2^ρ_e_) of bond critical points (BCPs, type (3, −1) in QTAIM). Likewise, non-covalent interaction analysis (NCI^47^) analyses were also plotted for the optimized clusters. The electron localization function (ELF) using the core–valence bifurcation index (CVB^48^) was calculated.

The DFT-optimized structures were considered for the generation of *cosmo* files at the BP86/def-TZVP theoretical level for COSMOtherm calculations using COSMOthermX software.^49^*Cosmo* files were considered to predict the properties of the considered HNADESs. COSMO-RS calculations were carried out by considering *cosmo* files for isolated HBA and HBD molecules.

For studying the properties of large clusters, considering computational limitations, calculations were carried out using DFT-TB simulations for CAR:C10AC with the DFTB+ program.^50^ The Slater–Koster (SK) files corresponding to the third-order parameterization for organic and biological systems (3OB) were applied.^51,52^ The self-consistent calculations were carried out with a 10^–6^ tolerance. The *k*-point meshes corresponded to a 4 × 4 × 4 distribution with the self-consistent calculations carried out with 10^–6^ tolerance. Initial cubic simulation boxes were built with the Amorphous Cell program in Biovia Materials Studio.^53^

Additionally, the interaction of the considered HNADESs with a model lipid bilayer formed by DPPC lipid molecules was studied using the COSMOperm method.^54^ This lipid bilayer may be considered as a model of a plasma cell membrane, and thus, it may be established as a model of the interaction of the considered molecules and cells, thus allowing to infer possible toxic effects of the materials, as it may be considered as a molecular initiating event of the adverse outcome pathway for the toxicity of nanomaterials.^55−57^ In the COSMOperm approach, based on the free energy profiles of the selected molecules of interest (HNADES components) within the membrane of interest (model DPPC lipid bilayer), the permeability of these molecules across the membrane is predicted, as well as the corresponding thermodynamic and structural factors of the molecule–membrane interactions, allowing to infer the possible molecular permeation across the bilayer, as well as the corresponding energy and entropic effects for cell permeation.

#### 2.3.2 Classical Molecular Dynamics Simulations

Classical MD simulations were carried out with MDynaMix v.5.2^58^ software for systems and conditions reported in Table S4 (Supporting Information). Force field parameters for the considered molecules are reported in Table S5 (Supporting Information). The reported parameters were derived from the SwissParam database^59^ (Merck Molecular Force Field), except for atomic charges, which were inferred from ChelpG charges obtained from DFT calculations reported in Section 2.3.1. Force field parameters were validated by comparing experimental and predicted density, with an average absolute deviation (AAD) of 0.46, and the results are reported in Figure S1 (Supporting Information). Initial cubic simulation boxes were built with the Packmol program.^60^ All simulations were carried out using periodic boundary conditions in the three space directions applying a three-step consecutive procedure: (i) 10 ns NVT simulations at 303 K for pre-equilibration purposes, (ii) 20 ns NPT equilibration step at 1 bar and 303 K, and (iii) 50 ns NVT production run at 303 K. The Nose–Hoover method^61^ was selected for pressure and temperature control, with 30 and 1000 ps as the time constants for the thermostat and barostat, respectively. The Tuckerman–Berne double time step algorithm^62^ (with long- and short-time steps of 1 and 10 fs, respectively) was applied to solve the equations of motion. The Ewald method^63^ (cutoff radius of 1.0 nm ) was applied for handling Coulombic interactions. Intermolecular interactions were described with the Lennard–Jones potential with a 10 Å cutoff distance and Lorentz–Berthelot mixing rules for cross terms. The visualization, analysis, and postprocessing of MD trajectories were carried out using VMD^64^ and TRAVIS programs.^65^

## 3 Results and Discussion

### 3.1 Thermodynamic Properties, Hydrophobicity, and Hydrogen Bonding

**Figure 2:**
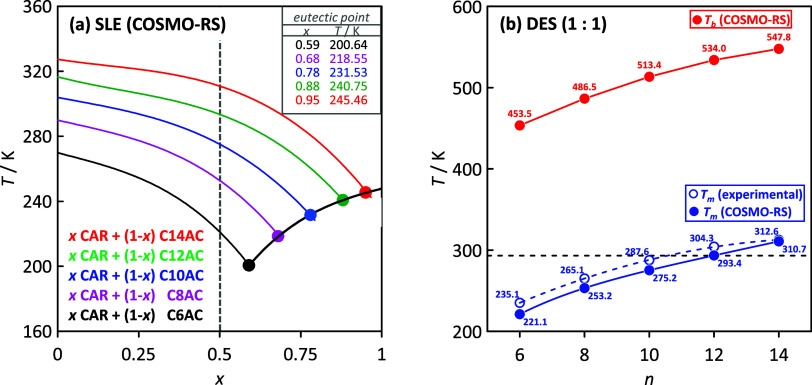
(a) Solid–liquid equilibria and eutectic points from COSMO-RS for the reported systems. (b) Melting (blue) and boiling (red) temperatures for CAR:organic acid (1:1) HNADESs as a function of the number of carbon atoms, *n*, in the fatty acid. The black dashed line indicates ambient temperature (298.15 K) for comparison purposes.

The HNADESs considered in this work are synthesized by a combination of CAR and organic acids. Previous studies have considered these HNADESs^66,67^ showing the formation of stable liquid phases, which is confirmed in this work for organic acids in the hexanoic to decanoic acid range. Nevertheless, to understand the evolution of liquid phases, the solid–liquid equilibria (SLE) of CAR + {organic acids} were predicted using the COSMO-RS method for the acid alkyl chain in the *n =* 6 (C6AC) to *n* = 14 (C14AC) range, where *n* stands for the number of carbon atoms in the considered organic acid (Figure 2a). The first relevant result stands on the fact that the eutectic composition appears for CAR-rich mixtures above the equimolar composition. Therefore, the systems experimentally and computationally considered in this work formed by CAR:organic acid in a (1:1) mole ratio are not eutectic mixtures from a purely thermodynamic point of view. Chen et al.^68^ recently proposed the use of the term “Low-Melting Mixtures Solvents” (LoMMSs) for those systems not being the true eutectic composition, but being liquid at close to ambient conditions and thus, having the same applicability of the true eutectic composition as green solvents. Likewise, the term DESs, and thus HNADESs, is largely used in the literature even for mixtures that are not real (thermodynamic) eutectic systems but are liquids at the working temperature. Therefore, the term HNADESs will be used in this work for the considered (1:1) mixtures, in parallel with the literature published in this research field. The calculated SLE show how increasing *n* leads to eutectic composition shifting toward CAR-rich mixtures. These eutectic mixtures with high CAR content would have lower hydrophobic character (e.g., for C14AC, the eutectic composition appears at a 0.95 CAR mole fraction), with low organic acid and physicochemical properties, very similar to those of neat CAR. Thus, equimolar mixtures were selected in this work, as all of them contained both CAR and organic acid, and they would have a large hydrophobic character, which would make them suitable for hydrocarbon solubilization. The properties of these equimolar mixtures are reported in Figure 2b as a function of *n* (organic acid size). The predicted melting point from COSMO-RS is compared with the data experimentally obtained in this work, showing how COSMO rightly predicts the trend with *n* and underpredicts the melting temperature (with the largest deviation of 14 K for *n* = 6). These results confirm the suitability of the COSMO-RS for SLE predictions in HNADESs. The reported results show how for *n* > 10, equimolar mixtures are not liquid at ambient (298.15 K) temperature; therefore, the following sections will consider only C6AC, C8AC, and C10AC. Considering the possible environmental impact of the selected HNADESs, the boiling temperature was also predicted via COSMO (Figure 2b), with the reported results showing very low volatile solvents, which combined with the natural (renewable) origin of CAR, and organic acids suggest a suitable alternative compared with usual volatile organic solvents.

**Figure 3:**
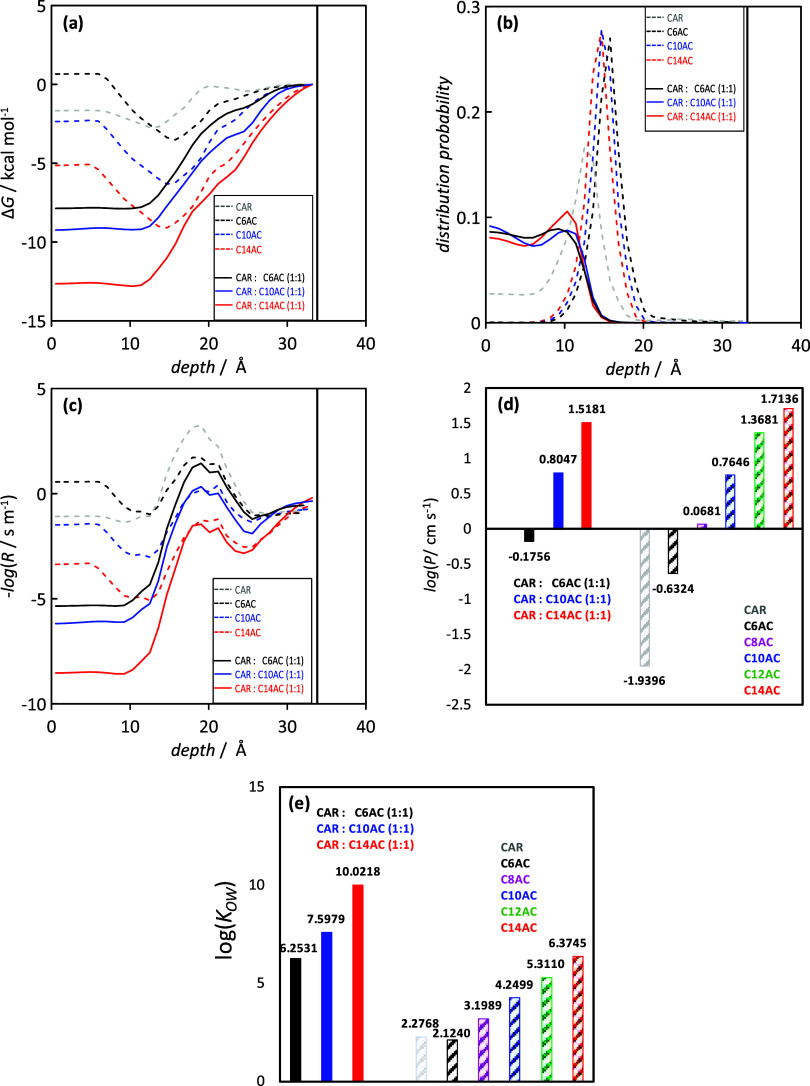
COSMOperm results for the interaction between the reported systems and the DPPC lipid bilayer: (a) free energy profiles, Δ*G*, (b) molecular distribution, (c) resistance, *R*, and (d) permeability, *P*. (e) COSMO-RS octanol–water partition coefficient, *K*_OW_. All values are calculated at 298.15 K. Vertical black lines indicate the external surface of the lipid bilayer.

To study the possible toxicological effects of the considered HNADESs, their interaction with a model lipid bilayer formed by DPPC lipid molecules was studied using the COSMOperm^54^ approach. The lipid bilayer can be a valuable model for studying how molecules and cells interact along the plasma membrane, allowing to infer potential toxic effects of materials. Because it represents an early event in the chain of cellular damage, the lipid bilayer model can be considered a key step in the adverse outcome pathway for material toxicity.^55−57^ Results in Figure 3a show the free energy, Δ*E*, for the permeation across the lipid bilayer. No energy barriers are inferred either for the individual HNADES components or for the CAR–organic acid complexes, leading to stabilization in the hydrophobic central region (i.e., alkyl chains) of the bilayer (depth = 0 Å represents the center of the lipid bilayer). The obtained molecular distribution reported in Figure 3b shows how for individual components molecules are placed in the hydrophobic central region, but upon the formation of CAR–organic acid complexes, molecules are able to penetrate deeper regions of the bilayer as a result of their larger hydrophobic nature. The resistance to penetration into the bilayer, Figure 3c, shows a small barrier close to the polar regions in all of the cases, but once it is overcome, penetration occurs easily. The calculated permeabilities are reported in Figure 3d. For the individual components, CAR shows the lowest permeability as a result of its lower hydrophobic nature, whereas for the organic acids, permeability increases with *n*. The formation of CAR–organic acid pairs leads to a synergistic effect regarding permeability for C6AC, with values larger than those of individual components, whereas for acids with long chains, permeability is determined by the acid. As the permeability into the lipid bilayer is calculated by the hydrophobic nature of the molecules, it was quantified via octanol–water partition coefficients, *K*_OW_, and also predicted via COSMO-RS (Figure 3e). For individual components, the hydrophobic character increases with *n* for acids, while for the CAR molecule, its *K*_OW_ value shows a non-negligible hydrophobic nature, remaining lower than that for acids with *n* ≥ 6. Upon the formation of CAR–organic acids, a remarkably synergistic effect is inferred, increasing the hydrophobic nature beyond that of pure components and thus pointing to largely hydrophobic fluids, which are very suitable for hydrocarbon-related applications.

**Figure 4:**
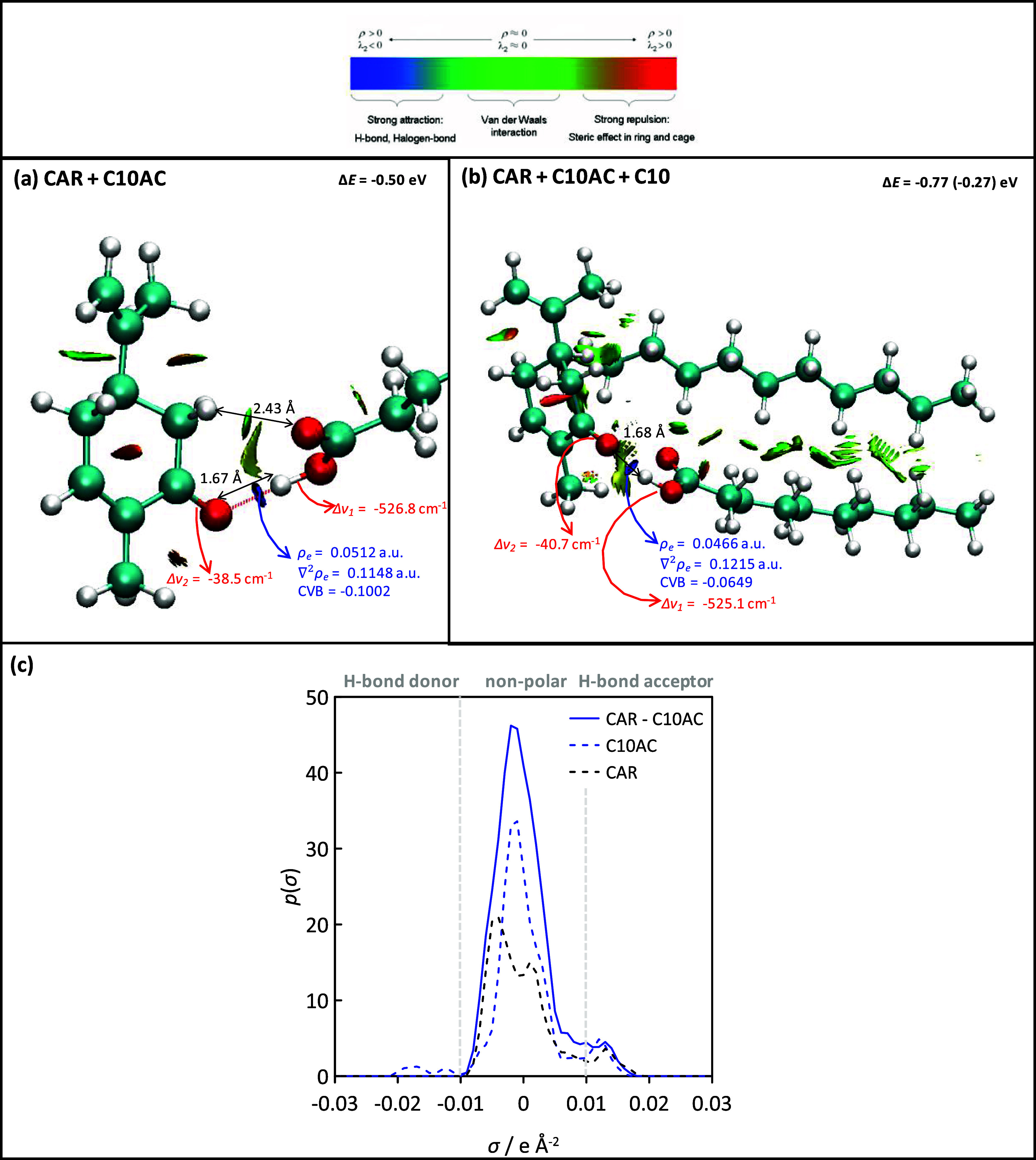
DFT-optimized structures of (a) CAR–C10AC and (b) CAR–C10AC–C10 clusters. Interaction energy, Δ*E*; QTAIM properties (ρ_e_ and ∇^2^ρ_e_); core valence bifurcation index (CVB) for the relevant hydrogen bonds; NCI analysis; and (red) shift of relevant vibration stretching frequencies (Δν_1_ and Δν_2_) upon cluster formation. (c) σ profiles for CAR and C10AC monomers and for the CAR–C10AC dimer, indicating the different molecular regions.

The results in the previous section have revealed how the formation of HNADESs via CAR–organic acid mixture leads to synergistic effects on their properties. This effect, as well as the actual formation of HNADESs, would be produced by the development of hydrogen bonding between CAR (acting as an HBA) and the organic acid (acting as an HBD). Hence, the nature of this interaction was studied via DFT using the corresponding dimer models. The CAR–organic acid interaction is characterized by large Δ*E*, corresponding to −0.56, −0.50, and −0.47 eV for C6AC, C10AC, and C14AC, respectively, i.e., a very minor *n* effect. The reported CAR–organic acid dimers, Figure 4, are characterized by a pseudo cyclic structure shape by the non-bonding interactions of (OH)acid–(CO)CAR (hydrogen bond) and (OH)acid–(CH)CAR (van der Waals). The presence of the CAR–acid hydrogen bond is confirmed by the NCI analysis, depicting a strong and localized (blue) spot. The strong van der Waals interaction regions explain the predicted large Δ*E*. The topology of the hydrogen bond is analyzed with the QTAIM approach, where 0.002 au < ρ_e_ < 0.04 au and 0.02 au < ∇^2^ρ_e_ < 0.139 au ranges correspond to hydrogen bonds, with larger values, meaning stronger interactions.^69^ The reported QTAIM data are above the upper limits of these ranges, thus manifesting very strong interactions, which agrees with the large negative CVB data in Figure 4. The development of hydrogen bonding is further confirmed by the calculated IR spectra with changes corresponding to the stretching vibrations of CO(CAR) and OH(organic acid). Both vibrations show a red shift, with the change inferred for the OH(organic acid) being especially large.

Although the considered systems have a large hydrophobic character because of the presence of large alkyl chains, as confirmed by properties such as the reported *K*_OW_ values, a certain polarity may be expected, mainly because of the presence of the CAR(CO) and organic acid (COOH) groups. The (DFT) calculated dipole moments for CAR and C10AC monomers are, respectively, 3.09 D (in agreement with values in the range of 3.2–3.8 D reported in the literature^70^) and 1.46 D (1.8 D in the literature^71^), and for the CAR–C10AC dimer (Figure 4a), a dipole moment of 2.59 D is inferred. Therefore, although the polarity of the considered HNADESs is not large, considering that the proposed HNADES is proposed for aliphatic extraction and solubilization, a certain amount of an aromatic hydrocarbon may be eluded into the aliphatic fraction. Nevertheless, results reported in Figure 4c for σ profiles reveal that most of the molecular regions upon formation of CAR–organic acid dimers (e.g., C10AC) via hydrogen bonding correspond to non-polar surfaces; thus, the possible interaction with aromatic molecules will be minimal.

The structure of the CAR–C10AC dimer was inferred in vacuum. To analyze the (solvent) environment effect, the dimer was optimized in an implicit (continuum) solvent by using COSMO to describe the surrounding solvent. Although the reported results indicated very low solubility of the considered HANDES in water, the simulations in an (implicit) solvent were carried out in water as a limiting effect for a largely polar surrounding medium. The structures of the CAR–C10AC dimer in vacuum and in (implicit) water were compared, leading to an RMSD between both structures of 0.1058. Likewise, the CAR(CO)–C10AC(OH) hydrogen bond changes from 1.67 Å in vacuum to 1.61 Å in (implicit) water. Therefore, even in a largely polar environment, the CAR–C10AC hydrogen bond remains.

**Figure 5:**
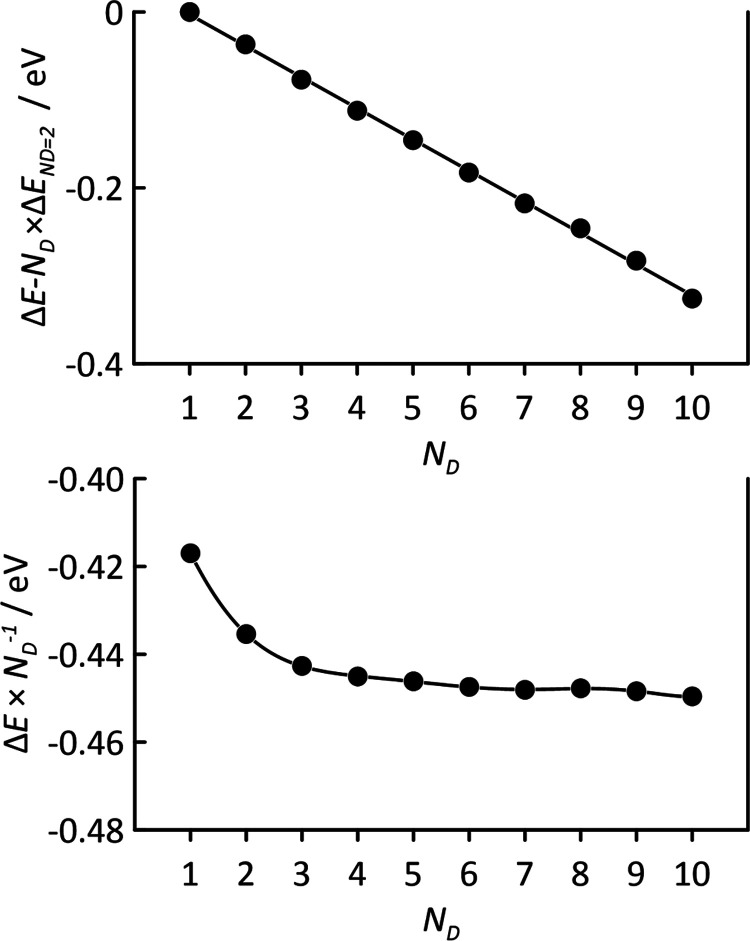
DFT-TB results for CAR–C10AC clusters as a function of containing dimers, *N*_D_, reporting interaction energy, Δ*E*.

In the case of HNADES liquid phases, intermolecular interactions will go beyond the minimal dimer model considered in Figure 4a. To analyze this effect, larger molecular models were prepared for CAR–C10AC, starting from the dimer proposed in Figure 4a and adding subsequent dimers, i.e., structures containing *N*_D_ dimers. The calculated Δ*E* evolves in a close to linear way with increasing molecular content, i.e., the main feature determining HNADES properties comes from the short-range contacts, hydrogen bonds, and van der Waals interactions between CAR and organic acids (Figure 4a). It should be remarked that the value of Δ*E* for *N*_D_ = 1 (i.e., dimer) being −0.42 eV from DFT-TB (Figure 5b) is very close to the value obtained from DFT (−0.50 eV, Figure 4a). Results in Figure 5b indicate a minor additional contribution to the Δ*E* beyond internal interaction among dimers, i.e., dimer–dimer interaction, being roughly 0.05 eV, which would correspond to van der Waals interactions among neighboring dimers directly hydrogen bonded.

As the considered HNADESs are proposed for dissolving hydrocarbons, the interaction between the CAR and organic acid dimers with hydrocarbon molecules was also studied via DFT. The results reported in Figure 4b show very efficient HNADES–hydrocarbon interaction as a consequence of the van der Waals contacts among the alkyl chains of the acid and hydrocarbon molecules (as predicted by NCI). At the same time, the CAR–organic acid interaction via hydrogen bonding does not change upon interaction with the hydrocarbon; only a minor change revealed by QTAIM data of the CAR–organic acid hydrogen bond is observed as a result of the acid molecule reorientation. Moreover, the σ profiles reported in Figure 4c indicate large non-polar regions in CAR–C10AC clusters, which will lead to efficient interactions with aliphatic hydrocarbon molecules via van der Waals contacts, as reported in Figure 4b. Therefore, the proposed HNADES is able to interact efficiently with hydrocarbon molecules, maintaining HNADES integrity, i.e., being a suitable solvent for hydrocarbon solubilization purposes.

### 3.2 Experimental Physicochemical and Spectroscopic Properties

**Figure 6:**
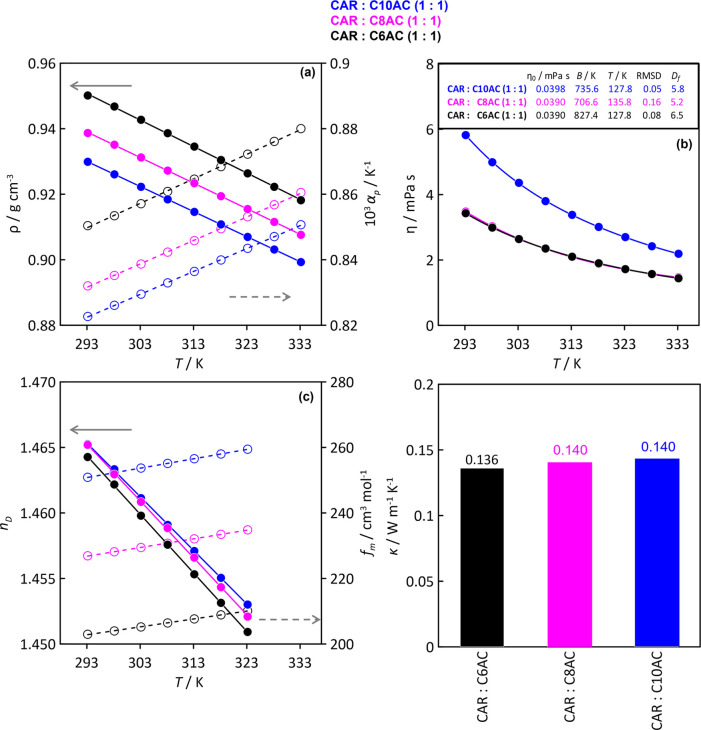
Experimental thermophysical properties of neat HNADESs. (a) Density, ρ, thermal expansion coefficient, α_p_, (b) dynamic viscosity, η, (c) refraction index, *n*_D_, free volume, (d) *f*_m_, and thermal conductivity, κ. Results for viscosity in panel (b) shows fit to the VFT equation with fitting parameters as well as Angel’s fragility parameter, *D*_f_. Thermal conductivity data reported at 298.15 K.

Relevant physicochemical properties were measured for CAR–organic acid (1:1) HNADESs considering C6AC, C8AC, and C10AC, as larger acids lead to solid HNADESs at close to ambient temperature (Figure 2). Results in Figure 6a show how all of the considered HNADESs are less dense than water (densities roughly in the 0.95–0.90 g cm^–3^ range) as a result of their hydrophobic character, with density decreasing as the organic acid alkyl chain increases. Likewise, α_p_, calculated using eq 1, implies compressible fluids (0.88–0.91 × 10^–3^ K^–1^ range). The values obtained for CAR:C10AC (1:1) are around those for other type V HNADESs, e.g., for Menthol:C10AC (1:1), the density and α_p_ are 0.90003 g cm^–3^ and 0.8162 × 10^–3^ K^–1^, respectively,^72^ and for CAR:C10AC (1:1), these values are 0.92989 g cm^–3^ and 0.823 × 10^–3^ K^–1^, respectively, all at 293.15. Thus, type V HNADESs present low density and are classified as compressible fluids, in contrast with type III DES, for which α_p_ values are frequently lower than 0.5 × 10^–3^ K^–1^^73,74^ The temperature effect both on density and α_p_ follows a regular linear evolution, with decreasing density and increasing compressibility upon heating (Figure 6a).

A pivotal property for the scalability of HNADESs for many relevant industrial operations, especially those involving heat and/or mass transfer, is viscosity, with many DESs (especially those belonging to type III because of their ionic nature) showing viscosities larger than 100 mPa·s,^75^ which may hinder the scaling up of these fluids. In the case of the HNADESs considered in this work, viscosities are lower than 6 mPa·s for all of the considered organic acids, decreasing with temperature in a non-Arrhenius trend and with increasing viscosity as organic acid chain length increases, reporting values close to those of common organic solvents used in the industry (Figure 6b). It is remarkable that even if the viscosities for the systems containing C6AC and C8AC as HBD are equivalent, the densities reported in Figure 6a are not equivalent but increase in a non-negligible trend. The molecular reason for the anomalous behavior of HNADES containing C8AC is analyzed in the following section from the MD study of liquid structuring as a function of organic acid chain length. The temperature evolution of viscosity for the considered HNADESs fitted to the VFT model, exemplified in eq 2, allowing the calculation, by means of eq 3, of *D*_f_ (fragility parameter), with the calculated values corresponding to fragile fluids^76^ (Figure 6c).

Additionally, refraction indexes were measured for the HNADESs, from which the free volume was calculated^77^ (Figure 6c). The obtained refraction indexes correspond to a moderately compressible fluid (Figure 6a), resulting from the available free volume (Figure 6c), because of the presence of an alkyl chain interacting through van der Waals forces (Figures 4 and 5). Moreover, these two properties evolve in a linear way upon heating, with the measured low refraction index corresponding to a large free volume. Thermal conductivity was measured for HNADESs (Figure 6d). Thermal conductivity slightly increases with increasing organic acid alkyl chain, showing values close to those of other type V HNADESs (e.g., 0.120 W m^–1^ K^–1^ for CIN:C10AC (1:1)^78^) and lower than those for type III DESs.^79^

**Figure 7:**
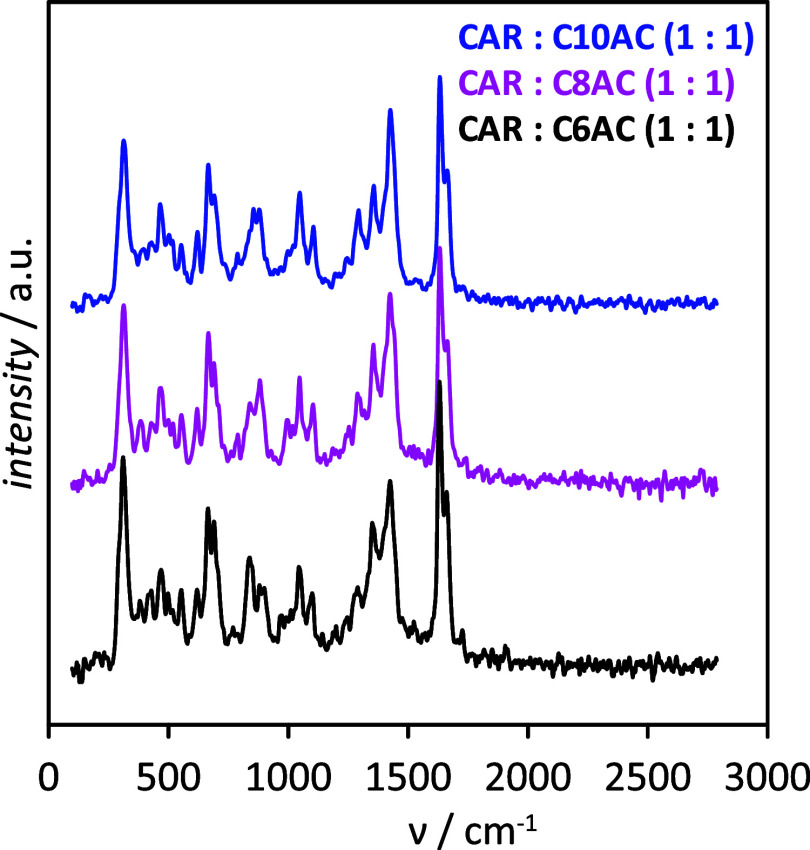
Raman spectra of the considered neat HNADESs at 298.15 K.

The Raman spectra for the considered HNADESs are reported in Figure 7. While a complete spectral assignment is not covered here, the aim was to identify the main spectral features for formation of HNADESs below 2800 cm^–1^. Those relevant peaks involving hydrogen bonds correspond to the stretching vibrations of CO groups in CAR and organic acids (those for the OH group in acids are beyond the 2800 cm^–1^ experimental limit). In the case of neat CAR, DFT results for the monomer led to a frequency for CO vibration of 1669 cm^–1^, which agrees with the experimental value obtained in this work as well as with the literature data.^80^ In the case of acids, e.g., C10AC (minor changes for the remaining considered acids), DFT results for the monomer led to a frequency for CO(C10AC) of 1733.8 cm^–1^, corresponding to non-hydrogen-bonded molecules, which shifted to 1660 cm^–1^ when C10AC dimers were considered because of acid–acid hydrogen bonding, in agreement with experimental results obtained in this work (1650 cm^–1^) and literature (1640 cm^–1^^81^). Once the CAR–organic acid HNADESs are formed (Figure 7), the peak for CO(organic acid) appears at 1649 cm^–1^, i.e., negligible changes compared with neat organic acids, whereas the peak for CO(CAR) appears at 1650 cm^–1^, i.e., a red shift of 19 cm^–1^ with regard to neat CAR (DFT results predicted a red shift of 38.5 cm^–1^ (Figure 4a)). Therefore, Raman results in Figure 7 confirm CAR–organic acid hydrogen bonding as well as that the organic acid self-association (hydrogen bonding) occurring in the pure acids is maintained upon formation of HNADESs; thus, both types of hydrogen bonds would remain in HNADES liquid structures. Likewise, C10AC in the neat state exhibited several relevant peaks corresponding to alkyl features: 878 cm^–1^ (CH_2_ rock), 1061 cm^–1^ (C–C stretch), 1292 cm^–1^ (CH_2_ twist), and 1430 cm^–1^ (CH_2_ wagging, scissoring, and deformation). These peaks remain upon HNADES formation with CAR; therefore, van der Waals-like interactions among alkyl chains of the acid molecules remain in the HNADES, leading to non-polar domains and increasing fluid hydrophobicity, in agreement with results in Figure 3.

**Figure 8:**
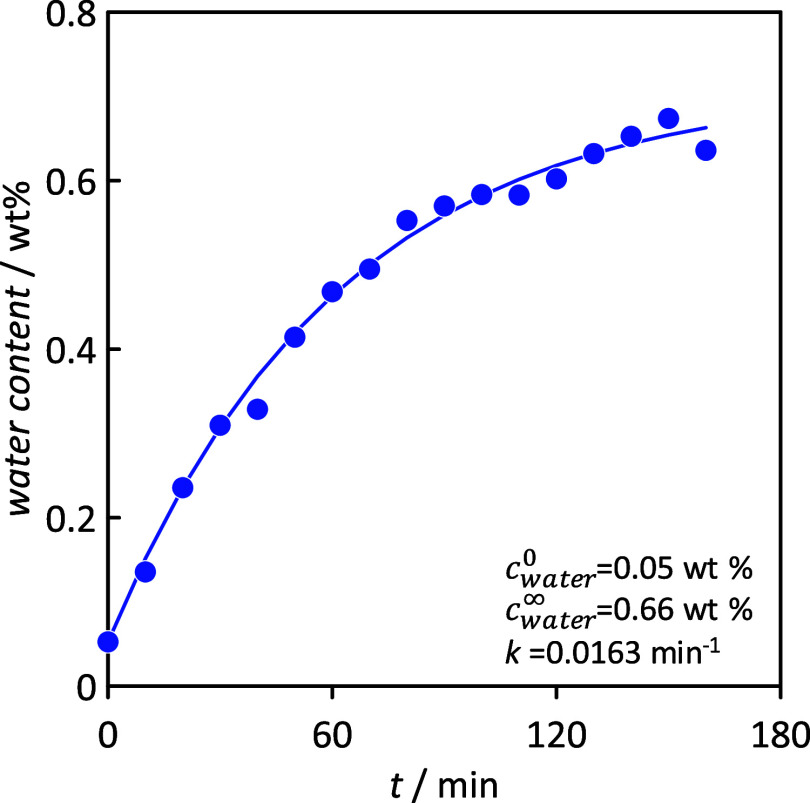
Kinetics for atmospheric water absorption in neat CAR:C10AC (1:1) at 298 K. Dashed lines show fitting to the kinetic model, eq 4.

Further, experimental proof of the hydrophobicity of the studied HNADESs was obtained by its capacity for capturing atmospheric water (Figure 8), including fitting to the kinetic model specified in eq 4. This property is largely relevant as in most of the possible technological applications of the considered HNADESs, they would be handled under open atmospheric conditions, which could lead to water sorption and thus to possible changes in the properties of HNADESs, considering the well-known effect of water.^82^ Samples after (vacuum) drying contained very low water (roughly 0.05 wt %), and after exposure to atmospheric water, this value increased only to 0.6 wt %, which confirms the large hydrophobicity of these fluids as well as the safe use under regular open conditions.

**Figure 9:**
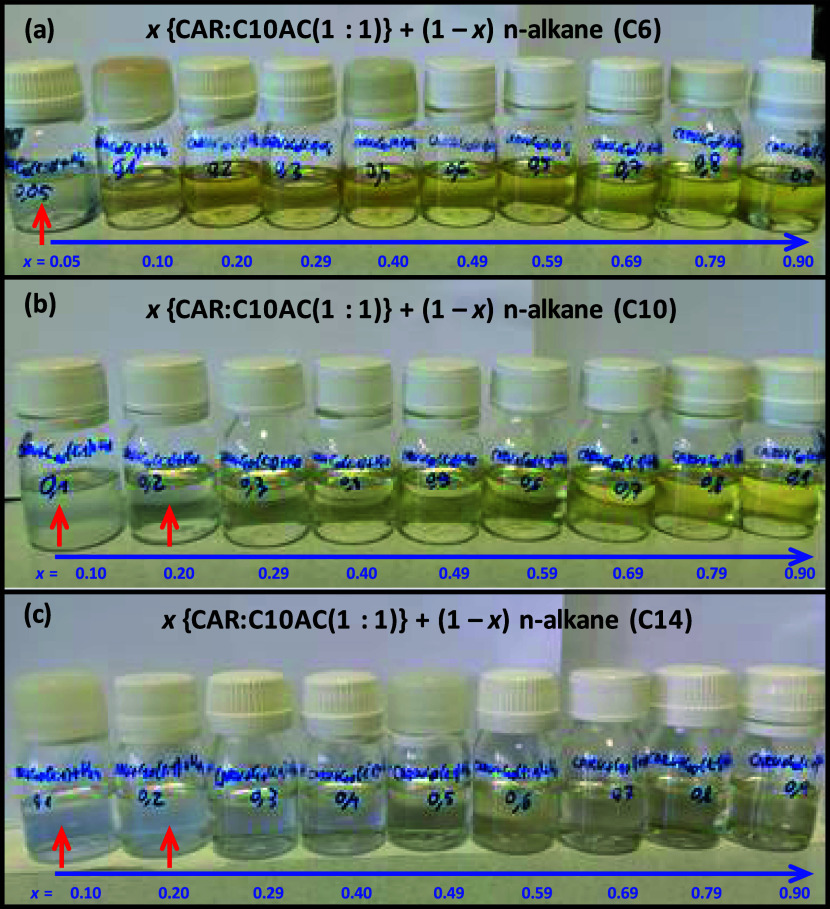
Samples of for *x* CAR:C10AC (1:1) + (1 – *x*) alkane (a) C6, (b) C10, or (d) C14 mixtures at 293.15 K. Red arrows indicate turbidity (i.e., phase separation) and blue arrows increasing HNADES content.

As the following objective of the work was to study the solubilization of hydrocarbons on the considered HNADESs, experimental studies were carried out for these mixtures. The first relevant result stands on the large solubility of alkanes (C6, C10, and C14) in the studied HNADES (CAR:C10AC (1:1), Figure 9). Phase separation only occurs for phases very rich in alkane, e.g., for *x* (HNADES mole fraction) = 0.05 (C6) or up to 0.2 (for C10 and C14). Therefore, whereas alkanes are largely soluble in the considered HNADESs, the reverse behavior is obtained for HNADESs in the alkane. This result is largely relevant as alkanes can be solubilized in HNADES,s but the HNADESs will not largely be present in alkane-rich phases.

Relevant thermophysical properties for HNADES + alkane mixtures are reported in Figure S2 (for CAR:C10AC HNADES) at isothermal conditions and Figures S3 and S4 (Supporting Information) as a function of temperature. Excess and related properties were calculated as described through eqs 1–8 in the Supporting Information. Excess molar volume data, reported in Figure S2a, correspond to largely non-ideal mixtures, evolving from negative values for C6 to almost null values for C10 and positive values for C14. As the considered organic acid is C10AC, and null excess volume is obtained for C10 alkane; it points to short alkane chains (C6) fitting into the holes of the HNADES, leading to contraction upon mixing, whereas for long chains (C14), expansion is inferred, as the long chain disrupts the HNADES structuring to allow alkane fitting. This behavior is confirmed by the minima in excess volume at *x*(HNADES) = 0.75 for C6, whereas the maxima for C14 appear at *x*(HNADES) = 0.50. The behavior of the excess thermal expansion coefficient (Figure S2b) is parallel to the one for excess volume, showing again how steric effects control the volumetric properties of the considered HNADES–alkane mixture. Excess partial molar volume for each component in the mixtures (Figure S2c) shows complex patterns. In the case of HNADES, C6 results confirm alkane molecules fitting into the HNADES structure, as inferred from the large negative excess partial molar volume. This value vanishes for C10 and is positive for C14, showing how large alkane molecules are poorly fitted into the HNADES liquid structure once the alkane overcomes the organic acid chain length. In the case of HNADES fitting into an alkane-rich liquid structure, the same trend is inferred. This behavior is confirmed by the values of partial excess molar volumes at infinite dilution (Figure S2d), which quantifies how isolated molecules are fitted into the liquid structure of almost pure liquid. The remarkably large (negative) values for C6 in HNADES (with C10AC) and for HNADES into C6 are in contrast with the positive values for C14. Likewise, values for HNADES + C6 evolve to more negative upon heating, whereas those for C14 increase to more positive values. This confirms not only how molecular fitting controls the volumetric properties and is a result of the differences among the alkyl chain lengths (of acids and alkanes) but also how these differences would lead to changes in the effectiveness of the van der Waals interaction, as reported in Figure 4b. Results in Figure S3 interpret the temperature effect on the considered volumetric excess properties with different effects for mixtures with C6, i.e., an increase of negative excess volume, to those with C14, i.e., larger expansion upon heating, as a consequence of the reinforcement, for C6, or the larger disruptive effect, for C14, of the chain length effect behavior with increasing temperature.

Dynamic viscosity and mixing dynamic viscosity are reported in Figure S4 (Supporting Information), exhibiting low viscous fluids along with negative mixing viscosity for all of the considered alkanes, but mixing viscosity decreases (in absolute value) when the alkane chain length is increased. This behavior corroborates once more how for short alkanes (C6), the fitting of alkane into the HNADES structure, with the C10AC chain longer than for C6, decreases viscosity because of both the interaction among alkyl chains and the appearance of holes into the fluid due to the asymmetry among the chain lengths. As the alkane chain length equals (C10) or overcomes (C14) the organic acid chain length (C10AC), this effect decreases, and so does the mixing viscosity. Nevertheless, having negative mixing viscosity, even for C14, indicates that alkyl–alkyl interaction is the main effect on the dynamic properties and allows the decrease in mixing viscosity for larger alkyl chains.

### 3.3 MD Simulations

To gain further insights into the intermolecular forces, the liquid (nano)structuring, and the dynamic properties of the considered fluids, MD simulations were carried out for neat HNADESs and HNADES + *n*-alkane mixtures. Experimental viscosity data reported in the previous section revealed low viscous fluids; thus, 50 ns long MD simulations led to equilibrated systems (Figure S5in the Supporting Information) and suitable prediction of properties.

**Figure 10:**
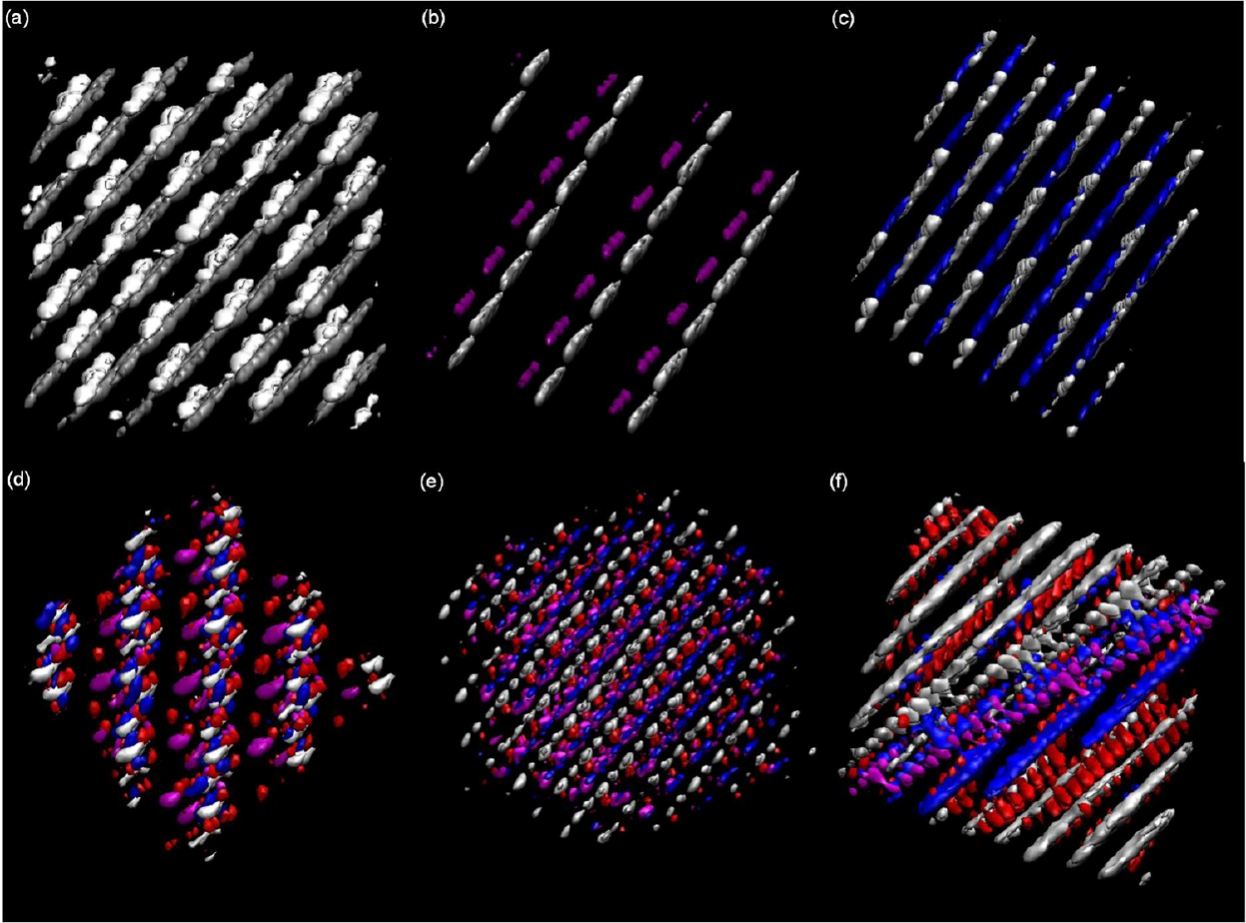
Volumetric maps for (a) CAR 1:1 C6AC, (b) CAR 1:1 C8AC, (c) CAR 1:1 C10AC, (d) *x* = 0.1 for *x* CAR:C10AC (1:1) + (1 – *x*) C10, (e) *x* = 0.5 for *x* CAR:C10AC (1:1) + (1 – *x*) C10, and (f) *x* = 0.9 for *x* CAR:C10AC (1:1) + (1 – *x*) C10 from MD simulations. Carvone is represented in silver, hexanoic acid in white, octanoic acid in magenta, and decanoic acid in blue; the oxygen donor atom of decanoic acid is depicted in red in panels (d–f).

Results in the previous section illustrated how alkyl–alkyl interactions developed a pivotal role in the liquid structuring both for neat HNADESs and for their mixtures with *n*-alkanes. This behavior was likewise analyzed via classical molecular dynamics simulations. An initial picture of the nanostructure of the considered fluids is presented as volumetric maps in Figure 10 for neat HNADES (Figure 10a–c) and for the mixtures of CAR:C10AC (1:1) as HNADES with C10 (Figure 10d–f) as a hydrocarbon. For mixtures, *x* CAR:C10AC (1:1) + (1 – *x*) C10 systems with *x* = 0.1, 0.5, and 0.9 were chosen. For neat HNADESs, C6AC (Figure 10a, in white) and C8AC (Figure 10b, in magenta) molecules are placed above carvone molecules (in silver), while for CAR:C10AC, the C10AC molecules (Figure 10c, in blue) are intertwined with carvone molecules rather than being placed on a different plane. This different behavior for C6AC/C8AC with C10AC would be the explanation of the experimental viscosity reported in Figure 6b because for C10AC, the intercalation of acid molecules would lead to a larger viscosity in contrast with the possible slipping behavior for C6AC/C8AC leading to lower, and similar for both acids, viscosity. For the mixtures, systems with *x* = 0.1 and *x* = 0.5 (in HNADES molar fraction), CAR (in silver), and C10AC (in blue) molecules are bonded, as shown by the position of the oxygen donor atom in decanoic acid (in red). For *x* = 0.9, the C10AC–C10 interaction is strong enough to concentrate the alkyl chains of acid molecules around C10, leaving the acid group (oxygen donor atom represented in red) in external positions, interacting with CAR molecules through hydrogen bond interactions. The DFT results (Figure 4b) indicated that the hydrogen bond in CAR 1:1 C10AC is maintained in the presence of C10 and that both 10-carbon-atom chains of acid and hydrocarbon interact via van der Waals with strong binding energies.

**Figure 11:**
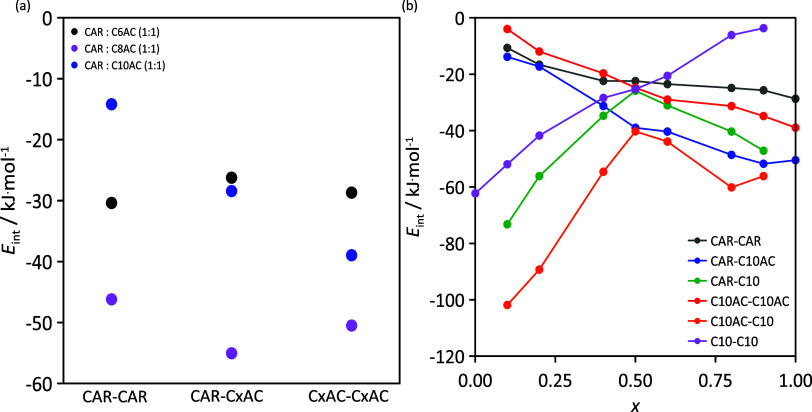
MD Lennard–Jones interaction energies for (a) neat HDESs and (b) *x* CAR: C10AC (1:1) + (1 – *x*) C10 mixtures.

The experimental density for neat HNADESs agrees with MD results, as shown in Figure S6a (Supporting Information), with deviations of −0.07, 0.56, and −0.54% for CAR:C6AC, CAR:C8AC, and CAR:C10AC, respectively. Likewise, suitable agreement is inferred for density in the (CAR:C10AC) HNADES + C10 mixtures (Figure S6b). The positive deviation of the mixture densities in the whole composition range indicates a negative excess molar volume, as obtained with experimental data (Figure S2a). The mechanism of intermolecular interaction as revealed in Figure S6 (Supporting Information) points to effective intermolecular interactions, which are quantified in Figure 11, considering Lennard–Jones intermolecular energies as the most relevant ones, as inferred from DFT results in Figure 4.

The strongest interaction belongs to the C10AC–C10 pair, where the van der Waals interactions (as represented in the NCI analysis and shown in Figure 4b) are responsible for the strong intermolecular forces developed between alkyl chains. Therefore, as expected, the CAR–C10AC and C10–C10 interaction energies are weaker than that of the C10AC–C10 pair for the whole composition range. For neat HNADESs (Figure 11a), the strongest intermolecular interaction energy corresponds to the CAR–organic acid pair, but strong acid–acid interactions are also reported. These results confirm that the formation of intermolecular hydrogen bonds is the driving force for the stabilization of the HNADES and, at the same time, that the acid–acid hydrogen bonding remains once the HNADES is formed. The CAR–CAR and CAR–acid interaction energies show almost negligible differences between the three HNADESs considered, whereas the acid–acid interaction energy decreases (more negative, i.e., stronger) linearly when increasing the number of carbon atoms in the acid alkyl chain. Likewise, it should be remarked that the van der Waals interactions developed between CAR molecules are remarkably strong, with an average value of (−28.4 ± 2.1) kJ/mol. The spatial distribution of CAR molecules in the bulk phase maximizes the CAR–CAR associations, as inferred from volumetric maps reported in Figure 11a–c.

**Figure 12:**
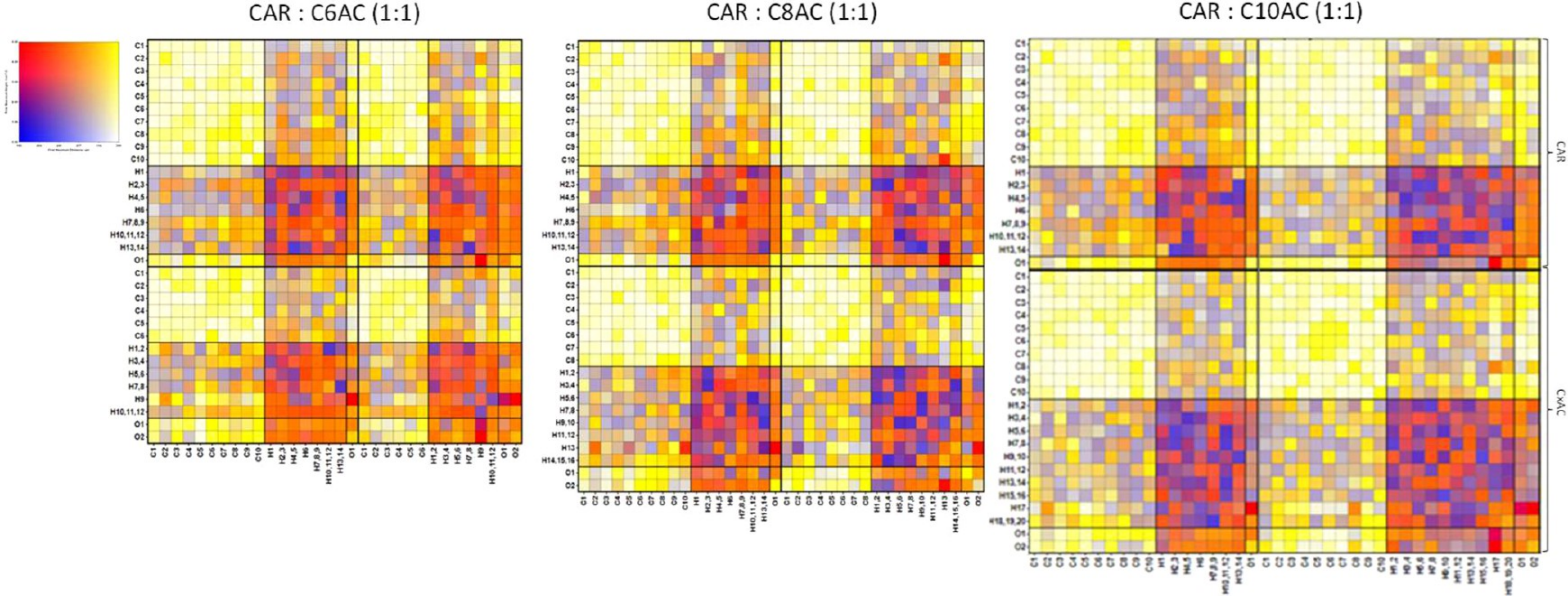
Connection matrix, *cmat*, analysis of the reported neat HDESs from MD simulations. The color in each square represents both the intensity and distance of the first maximum for the corresponding RDF.

**Figure 13:**
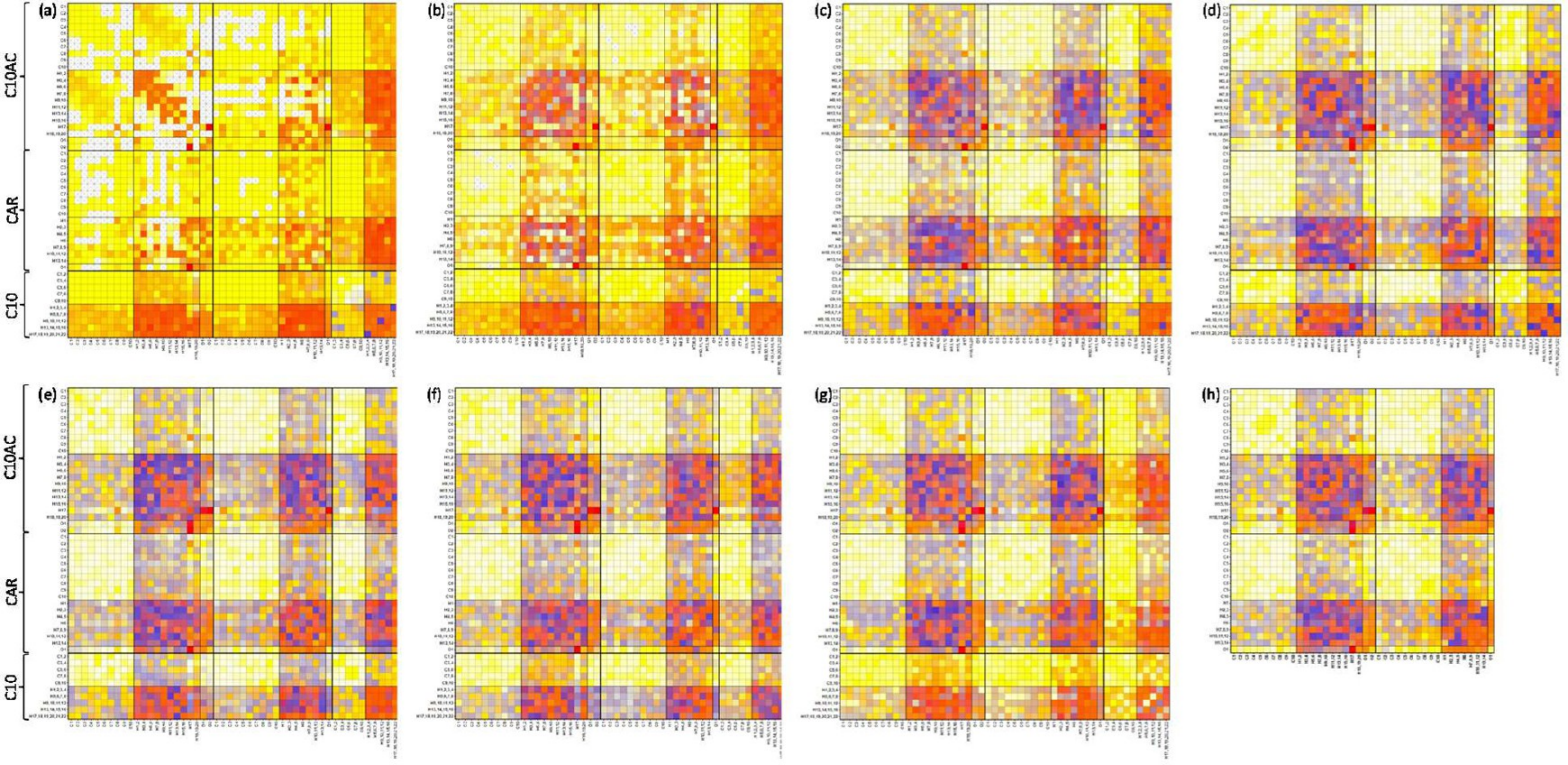
Connection matrix, *cmat*, analysis of the reported *x* CAR: C10AC (1:1) + (1 – *x*) C10 mixtures from MD simulations, ranging from (a) *x* = 0.1 to (g) *x* = 0.9. The panel (h) correspond to the *cmat* for the pure CAR:C10AC HNADES. The color in each square represents both the intensity and distance of the first maximum for the corresponding RDF.

**Figure 14:**
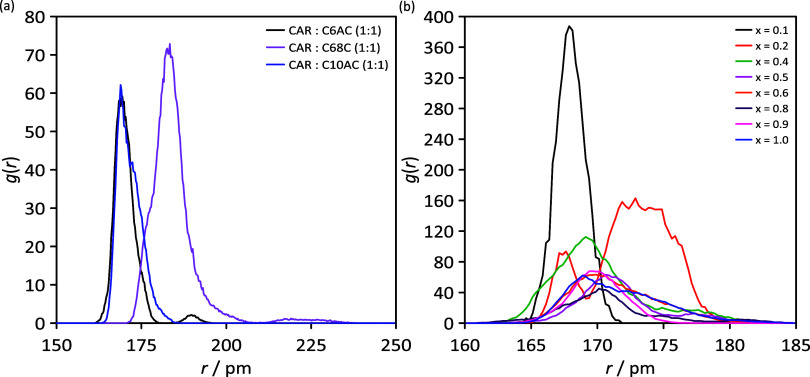
Site–site radial distribution functions, *g*(*r*), for the considered (a) neat HDESs and (b) *x* CAR: C10AC (1:1) + (1 – *x*) C10 mixtures from MD simulations.

In neat HNADESs, the hydrogen bond developed between the acceptor oxygen atom in CAR and the donor hydrogen atom in acids is responsible for the formation of the solvents (Figure 4a). Therefore, it is analyzed from the MD results. The distribution of intermolecular interactions is analyzed via the *connection matrix analysis (cmat*), a representation of the first peak for the site–site radial distribution function (RDF) of the corresponding atomic pairs quantified via the distance and height of these peaks, encoded in color, with red indicating strong interactions (hydrogen bond). The *cmat* for each neat HNADES is reported in Figure 12. The bright red color of O1(CAR)–H9(C6AC), O1(CAR)–H13(C8AC), and O1(CAR)–H17(C10AC) interactions indicates a very strong CAR–acid hydrogen bond, as well as the acid–acid intermolecular hydrogen bonds labeled as O2(C6AC)–H9(C6AC), O2(C8AC)–H13(C8AC), and O2(C10AC)–H17(C10AC). The effect of HNADES mixing with alkanes is also analyzed via *cmat* in Figure 13 from *x* = 0.1 (Figure 13a) to *x* = 1.0 (Figure 13h). Both homomolecular (O2–H17) and heteromolecular (O1–H17) hydrogen bonds are strong and maintained through the whole composition range studied, thus confirming that the presence of hydrocarbon (C10) in every molar fraction does not disrupt the hydrogen bond network established in the HNADES, as inferred from DFT cluster (Figure 4b) analysis, volumetric maps (Figure 10d–f), and interaction energies (Figure 11b). For mixtures, values for both the maximum distance and height for the first RDF are reported in Figure S7 (Supporting Information). The height and, therefore, the number of homomolecular hydrogen bonds (labeled as “HBD”) are greater than those for heteromolecular hydrogen bonds (labeled as “DES”) for the whole composition range. The extension of HBD–HBD hydrogen bonds is larger than that of HBA–HBD in the first solvation shell (of acid molecules), yet the strength of HBA–HBD hydrogen bonds is greater than that of homomolecular interactions, as reported in Figure 11b. To examine the CAR–C10AC hydrogen bond in more detail, radial, spatial, and combined distribution functions (CDFs) are computed. The RDFs for neat HNADESs (intermolecular hydrogen bonding CAR–acid; Figure 14a) show similar behavior for C6AC and C10AC HNADESs, whereas for C8AC HNADES, a different behavior is inferred with RDF peak shifting toward larger distances and being wider and more intense, which points to more hydrogen bonds but at larger distances, which agrees with volumetric maps (Figure 10a–c), where the distance between carvone and acid molecules are larger for CAR:C8AC (Figure 10b). Regarding mixtures, CAR–C10AC RDFs are reported in Figure 14b, which confirms hydrogen bonding in the whole composition range.

**Figure 15:**
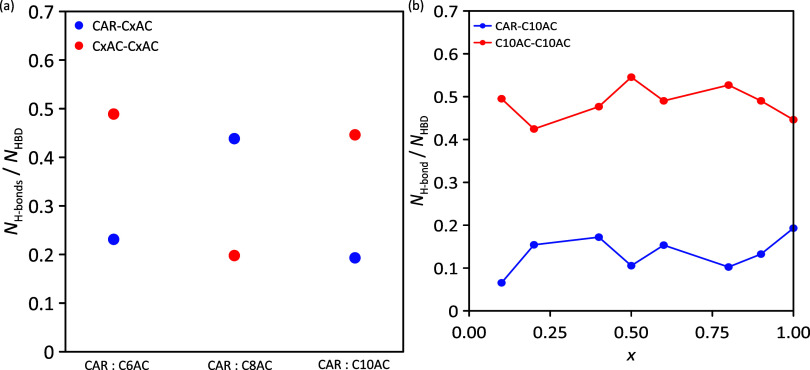
Average number of hydrogen bonds per hydrogen bond donor (HBD) molecule for (a) neat HDESs and (b) *x* CAR: C10AC (1:1) + (1 – *x*) C10 mixtures from MD simulations. HBA–HBD hydrogen bonds are depicted in blue, and HBD–HBD hydrogen bonds in red.

The integration of RDFs’ first peak for neat and mixed HNADESs allows to infer the average number of C10AC molecules in the first solvation shell of CAR, i.e., hydrogen bonding extension (Figure S8 in the Supporting Information). By combining these results with the number of hydrogen bonds per (organic acid) donor molecule for neat HNADES and mixtures (Figure 15a,15b), it can be concluded that the addition of a hydrocarbon to the HNADES does not affect the extension of developed hydrogen bonds, as the changes are almost negligible. In both neat HNADES and mixtures, the number of HBD–HBD hydrogen bonds is larger than that of HBA–HBD, with the exception of C8AC, as previously discussed (Figure 14a).

**Figure 16:**
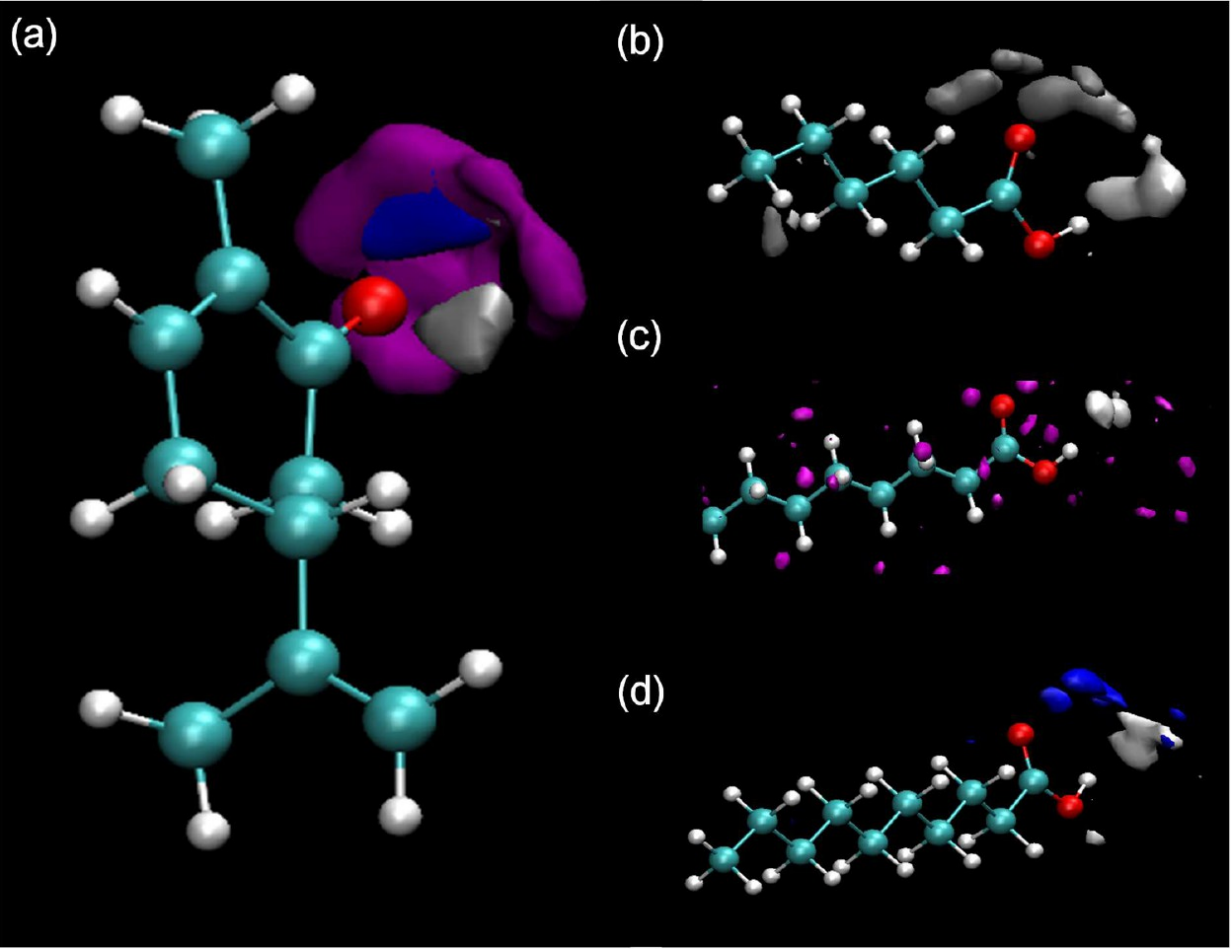
Spatial distribution functions (SDFs) for neat HDESs from MD simulations. Values are reported around carvone in panel (a), hexanoic acid in panel (b), octanoic acid in panel (c), and decanoic acid in panel (d) as reference molecules, showing the distribution of the corresponding observed molecules (carvone is depicted in white, hexanoic acid in gray, octanoic acid in magenta, and decanoic acid in blue).

**Figure 17:**
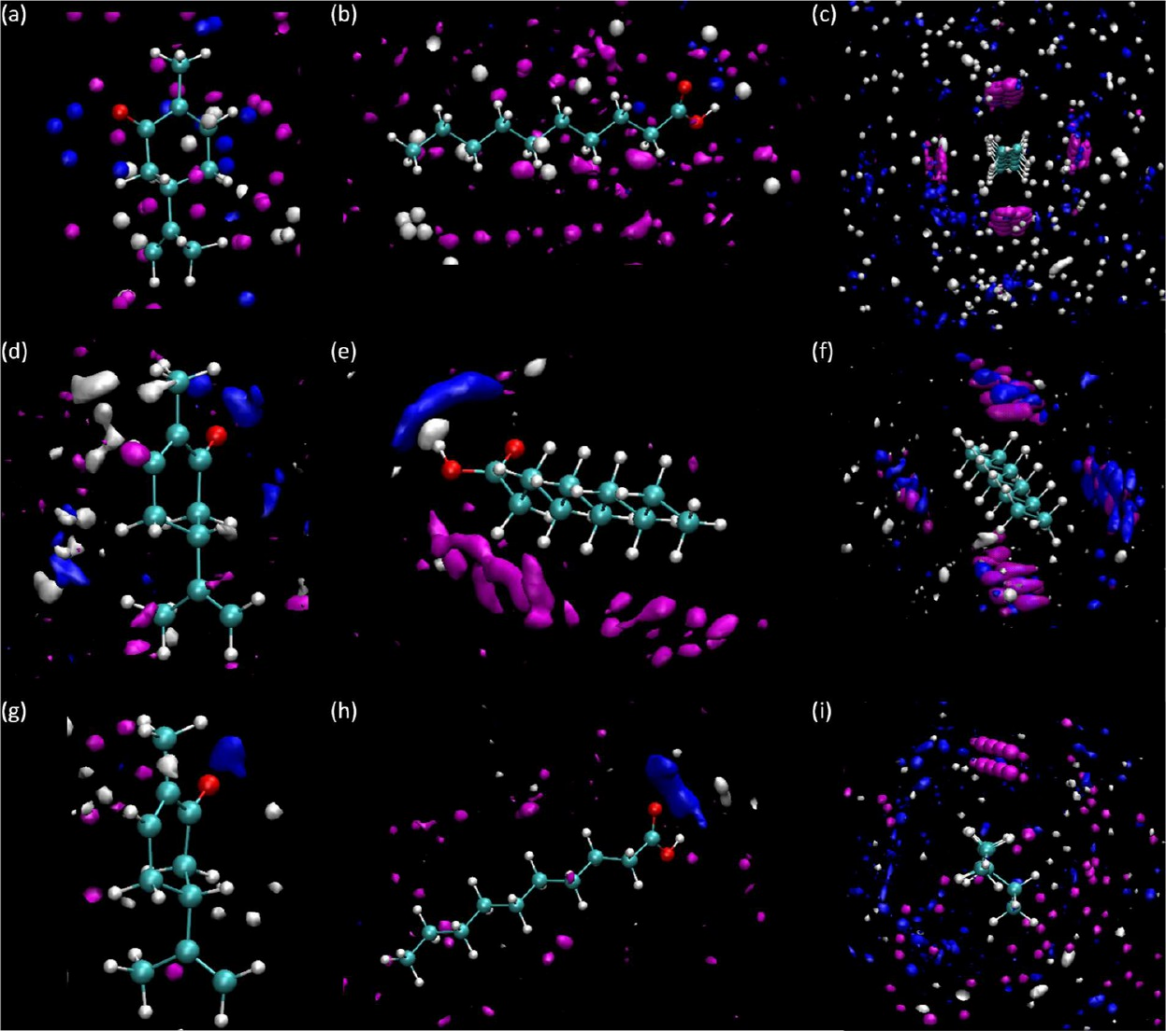
Spatial distribution functions (SDFs) for (a–c) *x* = 0.1 for *x* CAR: C10AC (1:1) + (1 – *x*) C10, (d–f) *x* = 0.5 for *x* CAR: C10AC (1:1) + (1 – *x*) C10, and (g–i) *x* = 0.9 for *x* CAR: C10AC (1:1) + (1 – *x*) C10 from MD simulations. Values are reported around carvone in panels (a, d, g), decanoic acid in panels (b, e, h), and decane in panels (c, f, i) as reference molecules, showing the distribution of the corresponding observed molecules (carvone is depicted in white, decanoic acid in blue, and decane in magenta).

Further analyses of intermolecular forces were carried out considering both the distance and angle for hydrogen bond interactions via spatial distribution functions (SDFs) and combined distribution functions (CDFs, considering separation and orientation). For neat HNADESs, the interaction with OH(organic acid) as a donor well localized around the CO acceptor in CAR is inferred for all of the considered acids (Figure 16a–d). For acid–acid hydrogen bonding, the behavior of C8AC differs from C6AC and C10AC, with the hydrogen donor atom (Figure 16c) placed around the whole molecule in contrast with the donor atoms for C6AC and C10AC (Figure 16b,d). In the case of alkane mixtures, increasing the molar fraction of HNADES affects the location of hydrogen bonding atoms, as hydrogen donor atoms for *x* = 0.1 (Figure 17a, in blue) are more distributed than those for *x* = 0.5 (Figure 17d) and *x* = 0.9 (Figure 17g), as well as for the oxygen acceptor atom in CAR (Figure 17b,e,h, in white). Figure 17c,f,i shows the distribution of CAR, in white, C10AC, in blue, and C10, in magenta, molecules, around C10. Carbon atoms for both C10AC and C10 interact with the C10 alkyl chain, confirming the results depicted in Figure 4b (DFT cluster analysis), Figure 10d,e, and interaction energies (Figure 11b), where the strongest interaction is C10AC–C10 for the whole composition range.

**Figure 18:**
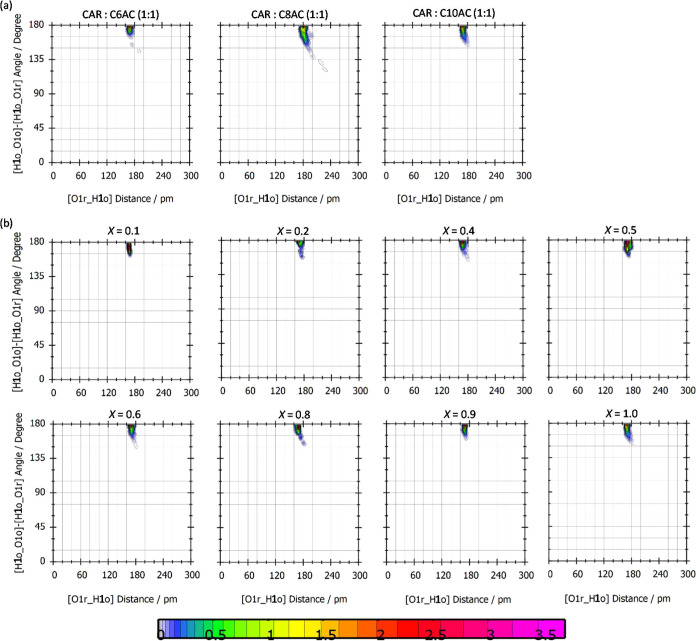
Combined distribution functions (CDFs) of HBA–HBD hydrogen bonds for the reported distance, *r*, and angle, φ, functions for (a) neat HDESs and (b) *x* CAR: C10AC (1:1) + (1 – *x*) C10 mixtures from MD simulations.

The CDFs represent the distance and angle (orientation) for the hydrogen bonding atoms. All of the simulated systems present an intense spot at around 180 pm and 180° (Figure 18); thus, both the type of HBD (organic acid) or the addition of C10 does not disrupt the hydrogen bond network.

**Figure 19:**
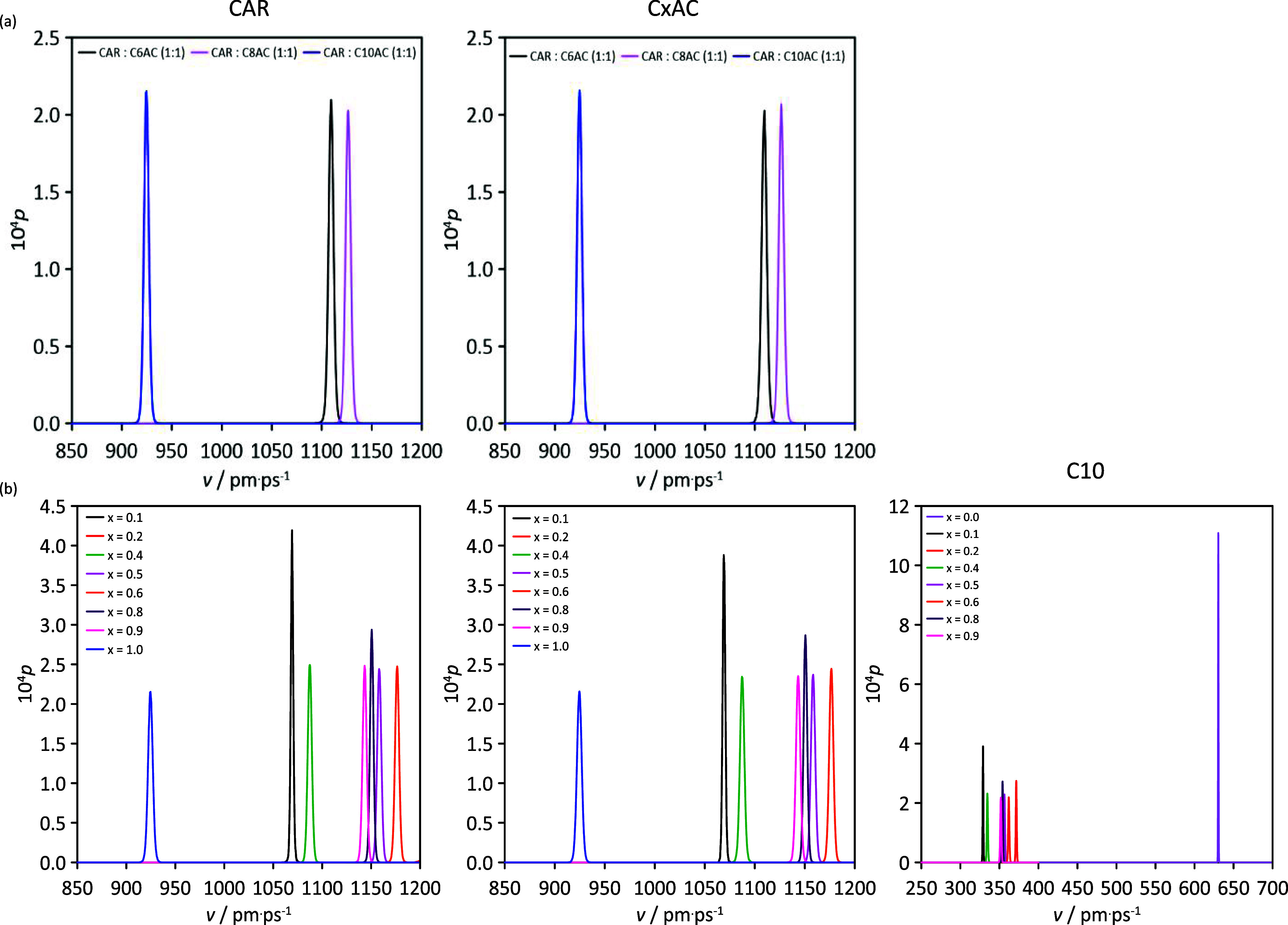
Velocity distribution functions (VDFs) of carvone, acid, and decane molecules for (a) neat HDESs and (b) *x* CAR: C10AC (1:1) + (1 – *x*) C10 mixtures from MD simulations.

**Figure 20:**
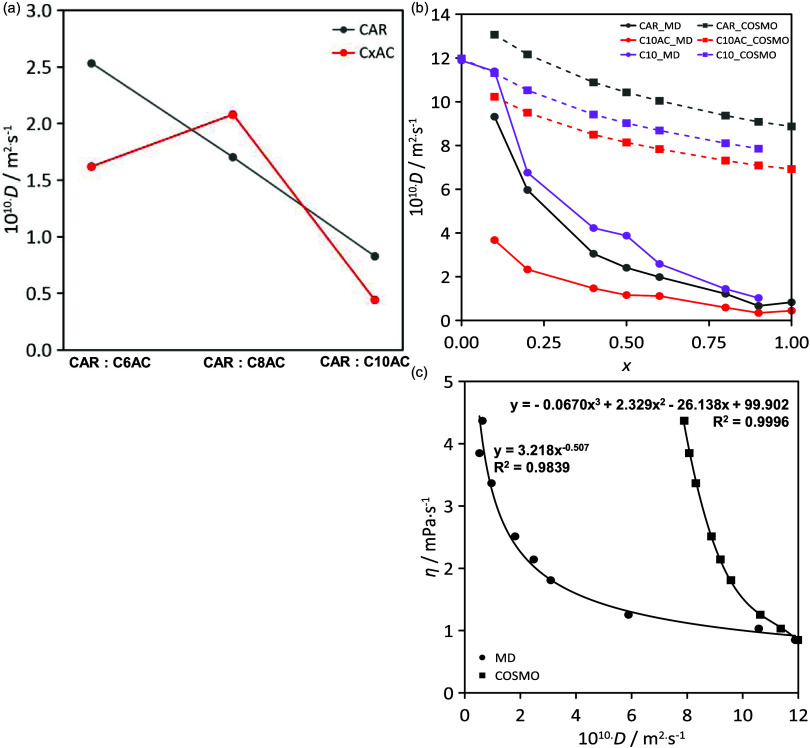
Diffusion coefficient, *D*, for (a) neat HDESs and (b) *x* CAR: C10AC (1:1) + (1 – *x*) C10 mixtures from MD simulations. In panel (b), the diffusion coefficient is calculated with both Travis (circles and straight lines) and COSMO-RS (squares and dotted lines) software. Panel (c) represents the experimental dynamic viscosity versus the MD-predicted diffusion coefficient from both Travis (circles) and COSMO-RS (squares) software. Fitting curves, equations, and *R*^2^ are included.

The dynamic of these fluids is analyzed via the velocity distribution functions (VDFs) for all of the considered molecules (Figure 19). For neat HNADESs, the results agree with the experimental viscosities (Figure 6b), with both CAR and organic acid molecules in C6AC and C8AC moving faster than in C10AC-based HNADES, which leads to larger viscosity in the neat HANDES. For mixtures, different behaviors are observed for HNADES and hydrocarbon: CAR and the acid molecules move faster than in pure HNADESs, but the velocity for C10 molecules decreases in comparison with pure C10, as a result of alkane molecules being trapped into the HNADES network (Figure 10). The dynamics of the fluids was also quantified via the calculated self-diffusion coefficients from MD simulations using mean square displacement and Einstein’s equation (Figure 20a). Results for neat HNADES show faster diffusion for CAR than for acids (with the exception of HNADES with C8AC, which agrees with results in previous sections), with the mobility decreasing with increasing acid alkyl chain length. This behavior results from acid molecules, leading to homo- and heteroassociations via hydrogen bonding, whereas CAR molecules are self-associated via van der Waals interactions and heteroassociated through hydrogen bonds, which agrees with the results in Figure 11a for interaction energy. In the case of mixtures (Figure 20b), self-diffusion coefficients were predicted from MD simulations and compared with those from COSMO-RS calculations with COSMO-RS overpredicting (i.e., faster diffusion) MD results. For neat HNADESs, CAR diffuses faster than C10AC but the differences among both ones are larger when considering the alkane–rich mixtures as the acid–acid self-association increases with HNADES content, then attracting CAR molecules which moves accordingly. Nevertheless, for HNADES-rich mixtures, the dynamic behavior resembles that for neat HNADES, thus confirming the fit of alkane into the HNADES liquid structuring, with alkane self-diffusion coefficients being close to those of HNADES components (i.e., decrease of alkane mobility when compared with neat alkane as reported in VDF; Figure 19b), with minor disruptive effects. The relationship between dynamic viscosity and self-diffusion is reported in Figure 20c, as it may be expected that both properties are related via the Stokes–Einstein equation. The inferred non-linearity points to the changes in the hydrodynamic radii of the diffusing species, i.e., changes in the size of the aggregated clusters upon mixture composition changes, and formation of small HNADES clusters in alkane-rich mixtures would lead to lower viscosities, whereas for HNADES-rich mixtures, the aggregation (homo and hetero) decreases molecular mobility and thus increases viscosity, evolving in a non-linear way.

**Figure 21:**
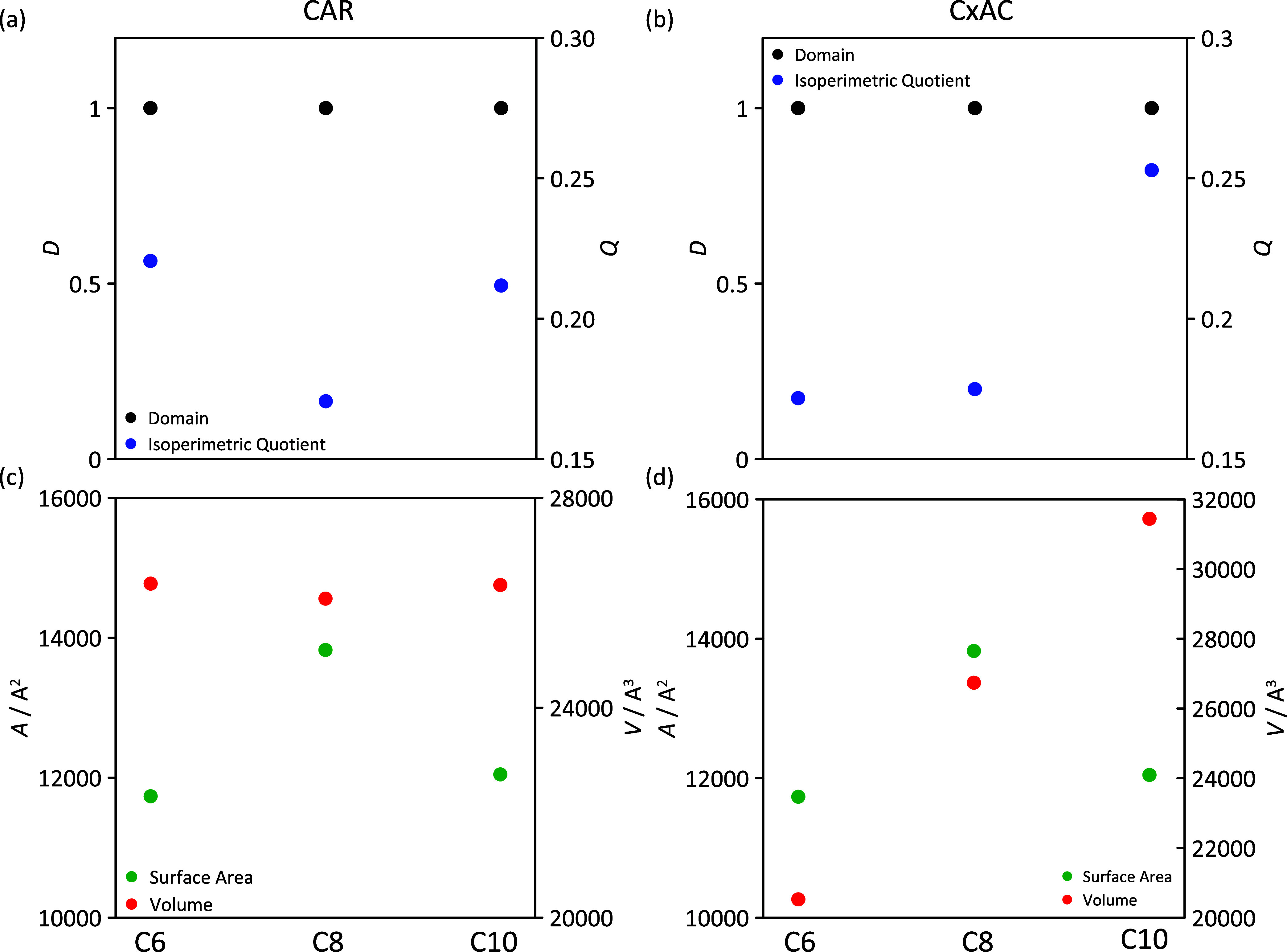
Domain analysis from MD simulations for neat HDESs for carvone molecules (a, c) and acid molecules (b, d). Color code: number of domains in black, isoperimetric quotient in blue, surface area in red, and volume in red.

**Figure 22:**
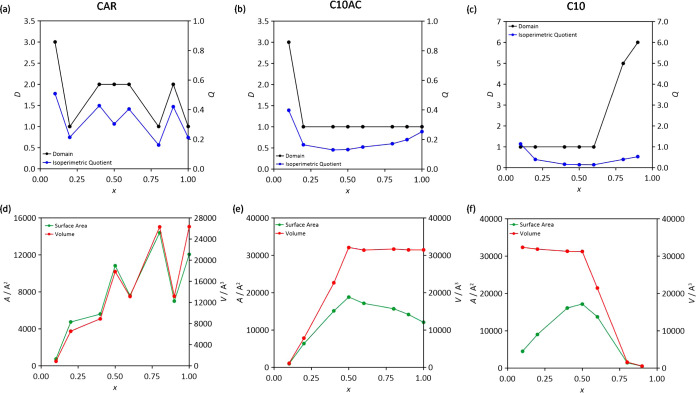
Domain analysis from MD simulations for *x* CAR: C10AC (1:1) + (1 – *x*) C10 mixtures for carvone molecules (a, d), decanoic acid molecules (b, e), and decane molecules (c, f). Color code: number of domains in black, isoperimetric quotient in blue, surface area in red, and volume in red.

The changes in clustering sizes inferred from dynamic properties were analyzed via the structuring and extension of domains into the considered neat (Figure 21) and mixed (Figure 22) fluids. For neat HNADES, the whole fluids are connected in a single domain, i.e., a hydrogen-bonded network connected along the whole fluid for all of the considered acids, forming non-spherical domains (low isoperimetric index) and thus larger domain area and volume. In the case of mixtures, HNADES components lead to large domains with the exception of alkane-rich mixtures, with the properties of these domains remaining unchanged for HNADES-rich mixtures, whereas alkane molecules are dispersed into the HNADES domains, as indicated in Figure 22c,f. These results agree with those in Figure 10 and prove how HNADES is able to fit (solve) very efficiently alkane molecules into their structure without disruption, leading to a suitable renewable solvent for hydrophobic solubilization purposes such as those related to alkane/hydrocarbons technologies.

## 4 Conclusions

A comprehensive analysis of the studied CAR–organic acid (1:1) HNADESs revealed quantitative characterization of key physicochemical properties, including density, compressibility, viscosity, and refractive index. The findings demonstrate that these HNADESs exhibit characteristics derived from their hydrophobic nature, with densities lower than that of water and compressibility in the expected range for fluids of this type. Importantly, the viscosity measurements showcase the suitability of these fluids for industrial applications involving heat and mass transfer, given their low viscosity even at low temperatures (values in the range of 3.4–5.8 at 293.15 K).

The solubilization behavior of alkanes in HNADESs was investigated, exhibiting high capacities across various alkane lengths. This is particularly significant for industrial processes requiring the dissolution of hydrophobic compounds. Examination of thermophysical properties in HNADES + alkane mixtures provided insights into their non-ideal mixing behavior, influenced by the alkane chain length. These findings elucidate the fundamental interactions governing HNADES behavior in complex mixtures, providing a critical foundation for the development of their application in diverse industrial processes. Multiscale modeling provided further insights into the intermolecular forces, liquid structuring, and dynamic properties of HNADESs. The simulations validated the experimental findings, revealing strong intermolecular interactions, particularly between alkyl chains. Analysis of intermolecular energies highlighted the significance of van der Waals interactions, especially in the C10AC–C10 pair, which exhibited the pivotal role for alkane solubilization as well as the steric effect arising from the size of involved alkyl chains in the considered organic acid with regard to a particular hydrocarbon. Moreover, examination of the hydrogen bonding networks in both neat HNADESs and mixtures demonstrated their persistence, even in the presence of *n*-alkanes. Dynamic properties, such as self-diffusion coefficients, quantified the molecular transport processes within the fluids. The self-diffusion coefficients revealed the interplay between the molecular structure and composition in fluid dynamics for neat HNADESs and HNADES + alkane mixtures.

Likewise, the solubilization of hydrocarbons exhibits a positive correlation with increasing alkyl chain length of the hydrophobic deep eutectic solvent. This phenomenon is observed across a spectrum of alkane chain lengths due to the development of appropriate hydrophobicity. The significance of this study lies in its comprehensive analysis of both hydrophobic deep eutectic solvent and hydrocarbon properties, coupled with an examination of their nanoscopic behavior. This investigation presents novel insights into the relationship between the alkyl chain length and the formation of nanoscopic domains, revealing complex molecular arrangements. These findings contribute substantially to the current understanding of hydrophobic solvent–hydrocarbon interactions and their structural implications at the nanoscale level.

Overall, the combined experimental and computational approach presented in this work offers a comprehensive study for understanding the behavior of HNADESs and their interactions with *n*-alkanes. These findings contribute significantly to the field of HNADES/DES research by providing a fundamental understanding of their physicochemical properties, behavior as solvents, and molecular dynamics. This knowledge is essential for the rational design of HNADESs as solvents for various industrial applications.

## Supporting Information Available

Additional experimental and computational details, including thermophysical properties and molecular dynamics results (PDF)
