# Supplementary material for: Insights into Carvone: Fatty Acid Hydrophobic NADES for Alkane Solubilization
Source: Energy Fuels. 2024 Dec 5;38(24):23633–53. doi: 10.1021/acs.energyfuels.4c03623 (PMC11664508; doi:10.1021/acs.energyfuels.4c03623)
Supplement: Supplementary file 1 — ef4c03623_si_001.pdf [file ef4c03623_si_001.pdf]

Supplementary Information

Insights into carvone : fatty acids hydrophobic NADES for alkanes solubilization

Nuria Aguilar,[a] Cristina Benito,[a] Sonia Martel-Martín,[b] Alberto Gutiérrez, [a] Sara Rozas,[a] Pedro A. Marcos,[c] Alfredo Bol-Arreba,[b,c] Mert Atilhan,[d] Santiago Aparicio,[a,b*]

[a] Department of Chemistry, University of Burgos, 09001 Burgos, Spain

[b] International Research Centre in Critical Raw Materials-ICCRAM, University of Burgos, 09001 Burgos, Spain

[c] Department of Physics, University of Burgos, 09001 Burgos, Spain

[d] Department of Chemical and Paper Engineering, Western Michigan University, Kalamazoo MI 49008-5462, USA

[*]Corresponding authors: sapar@ubu.es (S.A.)

Table S1. Specifications of the chemicals used in this work.

name	molar mass (g/mol)	purity (mass%)	source	CAS number	melting temperature / K
CAR	150.22	99.7	Sigma - Aldrich	6485-40-1	298
C6AC	116.16	99.5	Sigma – Aldrich	142-62-1	270
C8AC	144.21	99.5	Sigma – Aldrich	124-07-2	289
C10AC	172.26	99.5	Sigma – Aldrich	334-48-5	304
C12AC	200.32	99.5	Sigma – Aldrich	143-07-7	317
C14AC	228.37	99.0	Sigma – Aldrich	544-63-8	327
C6	86.18	99.9	Sigma – Aldrich	110-54-3	178
C10	142.29	99.5	Sigma – Aldrich	124-18-5	243
C14	198.39	99.0	Sigma - Aldrich	629-59-4	278

Table S2. Properties of HNADESs considered in this work.

CAR:C6AC (1:1) **CAR:C8AC (1:1)** **CAR:C10AC (1:1)**

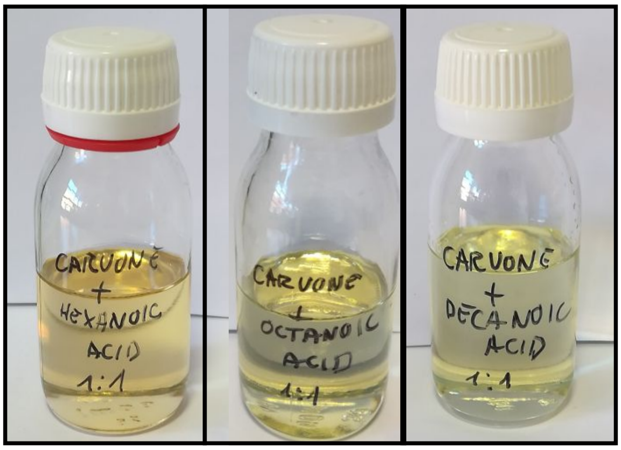

T / K	ρ / g cm^{-3}	α_p / g cm^{-3}	η / mPa s	n_D	κ / W m^{-1} K^{-1}
CAR : C6AC (1:1)					
293.15	0.95013	0.850	3.43	1.46427	—
298.15	0.94676	0.853	2.99	1.46217	0.136
303.15	0.94271	0.857	2.64	1.45979	—
308.15	0.93862	0.861	2.35	1.45758	—
313.15	0.93453	0.865	2.10	1.45532	—
318.15	0.93044	0.868	1.90	1.45314	—
323.15	0.92636	0.872	1.72	1.45092	—
328.15	0.92225	0.876	1.57	—	—
333.15	0.91816	0.880	1.44	—	—
CAR : C8AC (1:1)					
293.15	0.93867	0.832	3.48	1.46520	—
298.15	0.93510	0.835	3.03	1.46295	0.140
303.15	0.93121	0.839	2.65	1.46084	—
308.15	0.92720	0.842	2.34	1.45884	—
313.15	0.92335	0.846	2.09	1.45659	—
318.15	0.91940	0.850	1.88	1.45433	—
323.15	0.91547	0.853	1.71	1.45209	—
328.15	0.91154	0.857	1.58	—	—
333.15	0.90760	0.861	1.47	—	—
CAR : C10AC (1:1)					
293.15	0.92989	0.823	5.82	1.46525	—
298.15	0.92606	0.826	4.99	1.46333	0.143
303.15	0.92225	0.830	4.36	1.46113	—
308.15	0.91843	0.833	3.80	1.45909	—
313.15	0.91460	0.836	3.38	1.45709	—
318.15	0.91079	0.840	3.01	1.45505	—
323.15	0.90696	0.844	2.70	1.45301	—
328.15	0.90312	0.847	2.42	—	—
333.15	0.89928	0.851	2.19	—	—

Table S3. Properties of *x* CAR : C10AC (1 : 1) + (1 – *x*) hydrocarbon mixtures considered in this work as a function of temperature. Density (*ρ*) and dynamic viscosity (η).

	ρ / g cm^{-3}					
x DES	293.15	298.15	303.15	308.15	313.15	318.15
			C6			
0.0000	0.66099	0.65650	0.65196	0.64741	0.64280	0.63813
0.0983	0.72245	0.71818	0.71379	0.70938	0.70497	0.70055
0.1988	0.76950	0.76530	0.76110	0.75690	0.75260	0.74830
0.2880	0.80231	0.79820	0.79410	0.79000	0.78580	0.78158
0.3953	0.83420	0.83018	0.82620	0.82210	0.81795	0.81385
0.4928	0.85789	0.85402	0.85000	0.84600	0.84200	0.83791
0.5921	0.87820	0.87428	0.87035	0.86636	0.86239	0.85848
0.6885	0.89480	0.89094	0.88701	0.88310	0.87917	0.87531
0.7908	0.90940	0.90555	0.90170	0.89790	0.89400	0.89011
0.8951	0.92115	0.91740	0.91365	0.90982	0.90600	0.90220
1.0000	0.92989	0.92606	0.92225	0.91843	0.91460	0.91079
			C10			
0.0000	0.72970	0.72601	0.72227	0.71849	0.71469	0.71089
0.2979	0.81583	0.81206	0.80826	0.80443	0.80059	0.79675
0.3962	0.83766	0.83387	0.83007	0.82623	0.82238	0.81854
0.4973	0.85758	0.85378	0.84997	0.84613	0.84228	0.83844
0.5928	0.87440	0.87059	0.86678	0.86294	0.85909	0.85526
0.6799	0.88827	0.88446	0.88065	0.87682	0.87297	0.86914
0.7953	0.90483	0.90101	0.89720	0.89337	0.88953	0.88571
0.8972	0.91792	0.91410	0.91030	0.90648	0.90265	0.89884
			C14			
0.0000	0.76286	0.75934	0.75582	0.75232	0.74883	0.74533
0.2970	0.82134	0.81765	0.81398	0.81031	0.80666	0.80301
0.4027	0.83971	0.83601	0.83231	0.82861	0.82491	0.82121
0.5000	0.85585	0.85213	0.84840	0.84468	0.84094	0.83720
0.5947	0.87097	0.86719	0.86345	0.85968	0.85595	0.85219
0.7072	0.88823	0.88443	0.88065	0.87687	0.87307	0.86933
0.7959	0.90136	0.89754	0.89374	0.88995	0.88615	0.88235
0.8758	0.91286	0.90906	0.90526	0.90145	0.89762	0.89379

	η / mPa s		
x DES	293.15	303.15	313.15
	C6		
0.0000	0.322	0.301	0.287
0.0983	0.506	0.473	0.428
0.1988	0.758	0.664	0.598
0.2880	1.06	0.903	0.787
0.3972	1.49	1.25	1.06
0.4928	1.96	1.61	1.34
0.5921	2.53	2.04	1.67
0.6885	3.16	2.52	2.03
0.7908	3.94	3.06	2.44
0.8951	4.83	3.68	2.90
1.0000	5.82	4.36	3.38
	C10		
0.0000	0.970	0.850	0.750
0.2979	1.83	1.51	1.28
0.3962	2.23	1.79	1.50
0.4973	2.69	2.13	1.75
0.5928	3.18	2.48	2.01
0.6799	3.67	2.84	2.27
0.7953	4.39	3.35	2.64
0.8972	5.07	3.84	3.00
	C14		
0.0000	2.25	1.82	1.51
0.2970	2.85	2.29	1.86
0.4027	3.15	2.51	2.03
0.5000	3.48	2.74	2.20
0.5947	3.84	3.00	2.38
0.7072	4.33	3.33	2.63
0.7959	4.75	3.62	2.84
0.8758	5.16	3.90	3.05

Table S4. HNADESs systems considered for molecular dynamics simulations. *N* stands for the number of molecules of each type, *N_{atoms}* for the total number of atoms used in each system and *L* for the dimensions of the cubic simulation boxes in the studied pressure and temperature.

Compound A	Compound B	Compound C	*N*(A)	*N*(B)	*N*(C)	N_{atoms}	*P* / bar	*T* / K	*L* / Å
CAR	C6AC	--	100	100	0	4500	1	303	36.06
CAR	C8AC	--	100	100	0	5100	1	303	37.51
CAR	C10AC	--	100	100	0	5700	1	303	38.65
--	--	C10	0	0	100	3200	1	303	31.87
CAR	C10AC	C10	10	10	100	3770	1	303	33.71
CAR	C10AC	C10	25	25	100	4625	1	303	35.90
CAR	C10AC	C10	68	68	100	7076	1	303	41.54
CAR	C10AC	C10	100	100	100	8900	1	303	44.66
CAR	C10AC	C10	100	100	68	7876	1	303	42.92
CAR	C10AC	C10	100	100	25	6500	1	303	40.42
CAR	C10AC	C10	100	100	10	6020	1	303	39.38

Table S5. Forcefield parameterization for compounds studied in this work.

The general form of the applied force field is:

$$E = \sum_{bonds} k_r (r - r_{eq})^2 + \sum_{angles} k_\theta (\theta - \theta_{eq})^2 + E_{tor}$$
$$+ \sum_i \sum_j \left\{ 4\varepsilon_{ij} \left[\left(\frac{\sigma_{ij}}{r_{ij}} \right)^{12} - \left(\frac{\sigma_{ij}}{r_{ij}} \right)^{6} \right] + \frac{q_i q_j e^2}{4\pi\varepsilon_0 r_{ij}} \right\}$$

Dihedrals (E_{tor}) were described according to:

$$E_{tor} = \sum_{torsions} k_\phi (1 + \cos(m\phi - \delta))$$

Improper dihedrals were described according to:

$$E_{improper} = k_\phi(\phi - \phi_0)^2$$

[CAR]

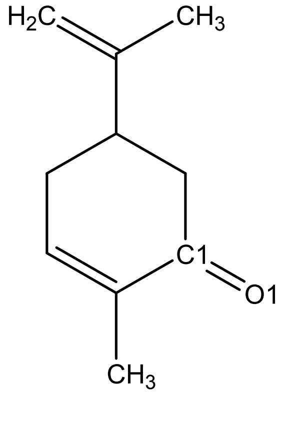

#	Atoms							
#	X	Y	Z	M	Q	sigma	epsilon	Number
#		(A)		(u)	(e)	(A)	(kJ/M)	
C	-0.193413	1.726493	-0.791143	12.011000	-0.591423	3.581410	0.234304	1
C	-0.086402	0.361762	-0.182180	12.011000	0.076876	3.723960	0.284512	2
H	0.547850	1.851061	-1.579922	1.007947	0.179335	2.351970	0.092048	3
H	-0.040165	2.499009	-0.036843	1.007947	0.155000	2.351970	0.092048	4
H	-1.166686	1.873361	-1.265906	1.007947	0.177556	2.351970	0.092048	5
C	-0.029969	0.135924	1.132088	12.011000	-0.218337	3.723960	0.284512	6
C	0.125560	-1.217703	1.761775	12.011000	-0.263425	3.581410	0.234304	7
H	-0.100923	0.984062	1.809603	1.007947	0.165086	2.351970	0.092048	8
C	0.798329	-2.201192	0.809108	12.011000	0.166037	3.581410	0.234304	9
H	-0.861800	-1.588220	2.065296	1.007947	0.127812	2.351970	0.092048	10
H	0.703981	-1.126979	2.683675	1.007947	0.105393	2.351970	0.092048	11
C	0.070084	-2.179838	-0.535989	12.011000	-0.396721	3.581410	0.234304	12
C1	-0.059243	-0.791227	-1.122827	12.011000	0.598021	3.563590	0.460240	13
H	-0.945325	-2.575751	-0.404338	1.007947	0.126593	2.351970	0.092048	14
H	0.555785	-2.802133	-1.288159	1.007947	0.118266	2.351970	0.092048	15
C	0.958239	-3.611813	1.326066	12.011000	0.262282	3.723960	0.284512	16
H	1.814142	-1.822987	0.627998	1.007947	0.039190	2.351970	0.092048	17
C	1.794425	-4.515317	0.453887	12.011000	-0.512476	3.581410	0.234304	18
H	1.173487	-5.000090	-0.304161	1.007947	0.153808	2.351970	0.092048	19
H	2.278274	-5.295662	1.042175	1.007947	0.150101	2.351970	0.092048	20
H	2.566199	-3.952839	-0.076703	1.007947	0.131734	2.351970	0.092048	21
C	0.398309	-4.057179	2.443433	12.011000	-0.652528	3.723960	0.284512	22
H	-0.230009	-3.435857	3.068896	1.007947	0.190691	2.351970	0.092048	23
H	0.570726	-5.072212	2.785405	1.007947	0.231453	2.351970	0.092048	24
O1	-0.160534	-0.628244	-2.318145	15.999400	-0.520325	3.029050	0.502080	25

#	Bonds			
	25			
#	N1	N2	R-eqv	Force
0	25	13	1.222	3899.333
0	3	1	1.093	1435.0745
0	15	12	1.093	1435.0745
0	5	1	1.093	1435.0745
0	13	12	1.492	1261.639
0	13	2	1.468	1374.553
0	1	2	1.482	1366.7245
0	1	4	1.093	1435.0745
0	12	14	1.093	1435.0745
0	12	9	1.508	1282.1115
0	19	18	1.093	1435.0745
0	2	6	1.333	2862.019
0	21	18	1.093	1435.0745
0	18	20	1.093	1435.0745
0	18	16	1.482	1366.7245
0	17	9	1.093	1435.0745
0	9	16	1.482	1366.7245
0	9	7	1.508	1282.1115
0	6	7	1.482	1366.7245
0	6	8	1.083	1556.724
0	16	22	1.333	2862.019
0	7	10	1.093	1435.0745
0	7	11	1.093	1435.0745
0	22	24	1.083	1556.724
0	22	23	1.083	1556.724

#	Angles			
	45			
#N1	N2	N3	A-eqv	Force
2	1	3	110.292	380.59
2	1	4	110.292	380.59
2	1	5	110.292	380.59
3	1	4	108.836	310.74
3	1	5	108.836	310.74

4	1	5	108.836	310.74		
1	2	6	122.141	404.68		
1	2	13	116.104	420.34		
6	2	13	111.297	328.2		
2	6	7	122.141	404.68		
2	6	8	121.004	322.18		
7	6	8	120.108	268.59		
6	7	9	109.445	443.23		
6	7	10	110.292	380.59		
6	7	11	110.292	380.59		
9	7	10	110.549	383		
9	7	11	110.549	383		
10	7	11	108.836	310.74		
7	9	12	109.608	512.48		
7	9	16	109.445	443.23		
7	9	17	110.549	383		
12	9	16	109.445	443.23		
12	9	17	110.549	383		
16	9	17	110.292	380.59		
9	12	13	107.517	467.91		
9	12	14	110.549	383		
9	12	15	110.549	383		
13	12	14	108.385	391.44		
13	12	15	108.385	391.44		
14	12	15	108.836	310.74		
2	13	12	116.853	666.04		
2	13	25	122.623	563.67		
12	13	25	124.41	564.87		
9	16	18	118.043	452.86		
9	16	22	122.141	404.68		
18	16	22	122.141	404.68		
16	18	19	110.292	380.59		
16	18	20	110.292	380.59		
16	18	21	110.292	380.59		
19	18	20	108.836	310.74		
19	18	21	108.836	310.74		
20	18	21	108.836	310.74		
16	22	23	121.004	322.18		
16	22	24	121.004	322.18		
23	22	24	119.523	219.8		
#	Dihedrals					
	136					
#N1	N2	N3	N4	A-eqv	Force	n
1	2	6	7	0	-0.8452	1
1	2	6	7	180	25.104	2
1	2	6	8	180	25.104	2
1	2	13	12	180	5.23	2
1	2	13	25	180	5.23	2
2	6	7	9	0	-1.0334	1
2	6	7	9	180	0.5732	2
2	6	7	9	0	-1.318	3
2	6	7	10	0	1.0502	1
2	6	7	10	180	-0.8577	2
2	6	7	10	0	-1.1213	3
2	6	7	11	0	1.0502	1
2	6	7	11	180	-0.8577	2
2	6	7	11	0	-1.1213	3
2	13	12	9	180	1.046	2
2	13	12	9	0	0.7322	3
2	13	12	14	0	0.2427	3
2	13	12	15	0	0.2427	3
3	1	2	6	0	1.0502	1
3	1	2	6	180	-0.8577	2
3	1	2	6	0	-1.1213	3
3	1	2	13	0	-0.2259	3

4	1	2	6	0	1.0502	1
4	1	2	6	180	-0.8577	2
4	1	2	6	0	-1.1213	3
4	1	2	13	0	-0.2259	3
5	1	2	6	0	1.0502	1
5	1	2	6	180	-0.8577	2
5	1	2	6	0	-1.1213	3
5	1	2	13	0	-0.2259	3
6	2	13	12	180	5.23	2
6	2	13	25	180	5.23	2
6	7	9	12	0	-0.615	1
6	7	9	12	180	0.9163	2
6	7	9	12	0	1.2217	3
6	7	9	16	0	0.6276	3
6	7	9	17	0	0.6736	1
6	7	9	17	180	-0.8577	2
6	7	9	17	0	0.3012	3
7	6	2	13	180	3.7656	2
7	9	12	13	0	0.1381	1
7	9	12	13	180	-0.3264	2
7	9	12	13	0	0.2971	3
7	9	12	14	0	1.3389	1
7	9	12	14	180	-1.318	2
7	9	12	14	0	0.5523	3
7	9	12	15	0	1.3389	1
7	9	12	15	180	-1.318	2
7	9	12	15	0	0.5523	3
7	9	16	18	0	0.8745	1
7	9	16	18	180	0.6192	2
7	9	16	18	0	0.5899	3
7	9	16	22	0	-1.0334	1
7	9	16	22	180	0.5732	2
7	9	16	22	0	-1.318	3
8	6	2	13	180	3.7656	2
8	6	7	9	0	0.1548	1
8	6	7	9	0	0.7489	3
8	6	7	10	0	-1.0962	1
8	6	7	10	180	-0.477	2
8	6	7	10	0	0.4351	3
8	6	7	11	0	-1.0962	1
8	6	7	11	180	-0.477	2
8	6	7	11	0	0.4351	3
9	12	13	25	0	1.7238	1
9	12	13	25	180	0.2929	2
9	12	13	25	0	0.682	3
9	16	18	19	180	-0.3849	2
9	16	18	19	0	0.4602	3
9	16	18	20	180	-0.3849	2
9	16	18	20	0	0.4602	3
9	16	18	21	180	-0.3849	2
9	16	18	21	0	0.4602	3
9	16	22	23	180	25.104	2
9	16	22	24	180	25.104	2
10	7	9	12	0	1.3389	1
10	7	9	12	180	-1.318	2
10	7	9	12	0	0.5523	3
10	7	9	16	0	0.6736	1
10	7	9	16	180	-0.8577	2
10	7	9	16	0	0.3012	3
10	7	9	17	0	0.5941	1
10	7	9	17	180	-2.8995	2
10	7	9	17	0	0.6569	3
11	7	9	12	0	1.3389	1
11	7	9	12	180	-1.318	2
11	7	9	12	0	0.5523	3

11	7	9	16	0	0.6736	1
11	7	9	16	180	-0.8577	2
11	7	9	16	0	0.3012	3
11	7	9	17	0	0.5941	1
11	7	9	17	180	-2.8995	2
11	7	9	17	0	0.6569	3
12	9	16	18	0	0.8745	1
12	9	16	18	180	0.6192	2
12	9	16	18	0	0.5899	3
12	9	16	22	0	-1.0334	1
12	9	16	22	180	0.5732	2
12	9	16	22	0	-1.318	3
13	12	9	16	0	0.6276	3
13	12	9	17	0	-0.5356	1
13	12	9	17	180	0.1213	2
14	12	9	16	0	0.6736	1
14	12	9	16	180	-0.8577	2
14	12	9	16	0	0.3012	3
14	12	9	17	0	0.5941	1
14	12	9	17	180	-2.8995	2
14	12	9	17	0	0.6569	3
14	12	13	25	0	1.3807	1
14	12	13	25	180	-2.9455	2
14	12	13	25	0	0.6443	3
15	12	9	16	0	0.6736	1
15	12	9	16	180	-0.8577	2
15	12	9	16	0	0.3012	3
15	12	9	17	0	0.5941	1
15	12	9	17	180	-2.8995	2
15	12	9	17	0	0.6569	3
15	12	13	25	0	1.3807	1
15	12	13	25	180	-2.9455	2
15	12	13	25	0	0.6443	3
17	9	16	18	180	-0.3849	2
17	9	16	18	0	0.4602	3
17	9	16	22	0	1.0502	1
17	9	16	22	180	-0.8577	2
17	9	16	22	0	-1.1213	3
18	16	22	23	180	25.104	2
18	16	22	24	180	25.104	2
19	18	16	22	0	1.0502	1
19	18	16	22	180	-0.8577	2
19	18	16	22	0	-1.1213	3
20	18	16	22	0	1.0502	1
20	18	16	22	180	-0.8577	2
20	18	16	22	0	-1.1213	3
21	18	16	22	0	1.0502	1
21	18	16	22	180	-0.8577	2
21	18	16	22	0	-1.1213	3
improper						
	15					
1	3	2	5	0	0	
1	3	2	4	0	0	
2	6	1	13	0	15.6565	
6	7	2	8	0	7.8324	
7	9	6	10	0	0	
7	9	6	11	0	0	
13	25	2	12	0	83.1026	
12	9	13	15	0	0	
12	15	13	14	0	0	
9	12	7	16	0	0	
9	12	7	17	0	0	
16	18	9	22	0	18.0665	
18	19	16	21	0	0	
18	19	16	20	0	0	

22	24	16	23	0	3.615

[C6AC]

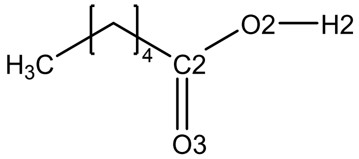

#	Atoms							
#	X	Y	Z	M	Q	sigma	epsilon	Number
#		(A)		(u)	(e)	(A)	(kJ/M)	
C2	-2.619388	1.132423	-0.029920	12.011000	0.711297	3.563590	0.460240	1
O2	-3.761008	0.414381	-0.031600	15.999400	-0.640772	3.153780	0.636386	2
H2	-4.484422	1.054634	-0.049935	1.007947	0.444301	0.400010	0.192464	3
O3	-2.623926	2.333848	-0.044888	15.999400	-0.575282	3.029050	0.502080	4
C	-1.399642	0.251500	-0.006836	12.011000	-0.090158	3.581410	0.234304	5
C	-0.096823	1.029066	-0.011657	12.011000	-0.080894	3.581410	0.234304	6
H	-1.467123	-0.422221	-0.865751	1.007947	0.061712	2.351970	0.092048	7
H	-1.478925	-0.389780	0.875750	1.007947	0.062695	2.351970	0.092048	8
C	1.135471	0.133961	0.019944	12.011000	-0.075361	3.581410	0.234304	9
H	-0.081714	1.708644	0.844278	1.007947	0.040710	2.351970	0.092048	10
H	-0.064306	1.667671	-0.898048	1.007947	0.040852	2.351970	0.092048	11
C	2.431555	0.930630	-0.000218	12.011000	0.242235	3.581410	0.234304	12
H	1.118119	-0.550238	-0.836252	1.007947	0.022440	2.351970	0.092048	13
H	1.111394	-0.497732	0.915498	1.007947	0.022039	2.351970	0.092048	14
C	3.683901	0.066329	0.014550	12.011000	-0.400712	3.581410	0.234304	15
H	2.446234	1.608720	0.858989	1.007947	-0.031274	2.351970	0.092048	16
H	2.441365	1.569080	-0.889056	1.007947	-0.030663	2.351970	0.092048	17
H	3.733267	-0.548540	0.916956	1.007947	0.090052	2.351970	0.092048	18
H	4.584276	0.683264	-0.021684	1.007947	0.097054	2.351970	0.092048	19
H	3.708885	-0.612830	-0.841280	1.007947	0.089730	2.351970	0.092048	20

#	Bonds			
	19			
#	N1	N2	R-eqv	Force
0	11	6	1.093	1435.0745
0	17	12	1.093	1435.0745
0	7	5	1.093	1435.0745
0	20	15	1.093	1435.0745
0	13	9	1.093	1435.0745
0	3	2	0.981	2229.09
0	4	1	1.222	3899.33
0	2	1	1.355	1746.72
0	1	5	1.492	1261.64
0	19	15	1.093	1435.0745
0	6	5	1.508	1282.11
0	6	9	1.508	1282.11
0	6	10	1.093	1435.0745
0	5	8	1.093	1435.0745
0	12	15	1.508	1282.11
0	12	9	1.508	1282.11
0	12	16	1.093	1435.0745
0	15	18	1.093	1435.0745
0	9	14	1.093	1435.0745

#	Angles			
	34			
#N1	N2	N3	A-eqv	Force
2	1	4	124.425	695.55
2	1	5	109.716	628.1
4	1	5	124.41	564.87
1	2	3	111.948	351.09
1	5	6	107.517	467.91
1	5	7	108.385	391.44
1	5	8	108.385	391.44

6	5	7	110.549	383		
6	5	8	110.549	383		
7	5	8	108.836	310.74		
5	6	9	109.608	512.48		
5	6	10	110.549	383		
5	6	11	110.549	383		
9	6	10	110.549	383		
9	6	11	110.549	383		
10	6	11	108.836	310.74		
6	9	12	109.608	512.48		
6	9	13	110.549	383		
6	9	14	110.549	383		
12	9	13	110.549	383		
12	9	14	110.549	383		
13	9	14	108.836	310.74		
9	12	15	109.608	512.48		
9	12	16	110.549	383		
9	12	17	110.549	383		
15	12	16	110.549	383		
15	12	17	110.549	383		
16	12	17	108.836	310.74		
12	15	18	110.549	383		
12	15	19	110.549	383		
12	15	20	110.549	383		
18	15	19	108.836	310.74		
18	15	20	108.836	310.74		
19	15	20	108.836	310.74		
#	Dihedrals					
	128					
#N1	N2	N3	N4	A-eqv	Force	n
1	5	6	9	0	0.1381	1
1	5	6	9	180	-0.3264	2
1	5	6	9	0	0.2971	3
1	5	6	10	0	-0.5356	1
1	5	6	10	180	0.1213	2
1	5	6	11	0	-0.5356	1
1	5	6	11	180	0.1213	2
2	1	5	6	0	-0.2469	1
2	1	5	6	180	-0.6987	2
2	1	5	6	0	0.4226	3
2	1	5	7	180	-1.3054	2
2	1	5	7	0	0.6904	3
2	1	5	8	180	-1.3054	2
2	1	5	8	0	0.6904	3
3	2	1	4	0	3.4769	1
3	2	1	4	180	12.87	2
3	2	1	4	0	-0.1213	3
3	2	1	5	0	-2.4393	1
3	2	1	5	180	10.6232	2
3	2	1	5	0	-1.1422	3
4	1	5	6	0	1.7238	1
4	1	5	6	180	0.2929	2
4	1	5	6	0	0.682	3
4	1	5	7	0	1.3807	1
4	1	5	7	180	-2.9455	2
4	1	5	7	0	0.6443	3
4	1	5	8	0	1.3807	1
4	1	5	8	180	-2.9455	2
4	1	5	8	0	0.6443	3
5	6	9	12	0	0.2134	1
5	6	9	12	180	1.4267	2
5	6	9	12	0	0.6945	3
5	6	9	13	0	1.3389	1
5	6	9	13	180	-1.318	2
5	6	9	13	0	0.5523	3

5	6	9	14	0	1.3389	1
5	6	9	14	180	-1.318	2
5	6	9	14	0	0.5523	3
6	9	12	15	0	0.2134	1
6	9	12	15	180	1.4267	2
6	9	12	15	0	0.6945	3
6	9	12	16	0	1.3389	1
6	9	12	16	180	-1.318	2
6	9	12	16	0	0.5523	3
6	9	12	17	0	1.3389	1
6	9	12	17	180	-1.318	2
6	9	12	17	0	0.5523	3
7	5	6	9	0	1.3389	1
7	5	6	9	180	-1.318	2
7	5	6	9	0	0.5523	3
7	5	6	10	0	0.5941	1
7	5	6	10	180	-2.8995	2
7	5	6	10	0	0.6569	3
7	5	6	11	0	0.5941	1
7	5	6	11	180	-2.8995	2
7	5	6	11	0	0.6569	3
8	5	6	9	0	1.3389	1
8	5	6	9	180	-1.318	2
8	5	6	9	0	0.5523	3
8	5	6	10	0	0.5941	1
8	5	6	10	180	-2.8995	2
8	5	6	10	0	0.6569	3
8	5	6	11	0	0.5941	1
8	5	6	11	180	-2.8995	2
8	5	6	11	0	0.6569	3
9	12	15	18	0	1.3389	1
9	12	15	18	180	-1.318	2
9	12	15	18	0	0.5523	3
9	12	15	19	0	1.3389	1
9	12	15	19	180	-1.318	2
9	12	15	19	0	0.5523	3
9	12	15	20	0	1.3389	1
9	12	15	20	180	-1.318	2
9	12	15	20	0	0.5523	3
10	6	9	12	0	1.3389	1
10	6	9	12	180	-1.318	2
10	6	9	12	0	0.5523	3
10	6	9	13	0	0.5941	1
10	6	9	13	180	-2.8995	2
10	6	9	13	0	0.6569	3
10	6	9	14	0	0.5941	1
10	6	9	14	180	-2.8995	2
10	6	9	14	0	0.6569	3
11	6	9	12	0	1.3389	1
11	6	9	12	180	-1.318	2
11	6	9	12	0	0.5523	3
11	6	9	13	0	0.5941	1
11	6	9	13	180	-2.8995	2
11	6	9	13	0	0.6569	3
11	6	9	14	0	0.5941	1
11	6	9	14	180	-2.8995	2
11	6	9	14	0	0.6569	3
13	9	12	15	0	1.3389	1
13	9	12	15	180	-1.318	2
13	9	12	15	0	0.5523	3
13	9	12	16	0	0.5941	1
13	9	12	16	180	-2.8995	2
13	9	12	16	0	0.6569	3
13	9	12	17	0	0.5941	1
13	9	12	17	180	-2.8995	2

13	9	12	17	0	0.6569	3
14	9	12	15	0	1.3389	1
14	9	12	15	180	-1.318	2
14	9	12	15	0	0.5523	3
14	9	12	16	0	0.5941	1
14	9	12	16	180	-2.8995	2
14	9	12	16	0	0.6569	3
14	9	12	17	0	0.5941	1
14	9	12	17	180	-2.8995	2
14	9	12	17	0	0.6569	3
16	12	15	18	0	0.5941	1
16	12	15	18	180	-2.8995	2
16	12	15	18	0	0.6569	3
16	12	15	19	0	0.5941	1
16	12	15	19	180	-2.8995	2
16	12	15	19	0	0.6569	3
16	12	15	20	0	0.5941	1
16	12	15	20	180	-2.8995	2
16	12	15	20	0	0.6569	3
17	12	15	18	0	0.5941	1
17	12	15	18	180	-2.8995	2
17	12	15	18	0	0.6569	3
17	12	15	19	0	0.5941	1
17	12	15	19	180	-2.8995	2
17	12	15	19	0	0.6569	3
17	12	15	20	0	0.5941	1
17	12	15	20	180	-2.8995	2
17	12	15	20	0	0.6569	3
improper						
	11					
1	2	5	4	0	84.9101	
5	6	1	7	0	0	
5	7	1	8	0	0	
6	9	5	11	0	0	
6	11	5	10	0	0	
9	12	6	13	0	0	
9	13	6	14	0	0	
12	15	9	17	0	0	
12	17	9	16	0	0	
15	20	12	19	0	0	
15	20	12	18	0	0	

[C8AC]

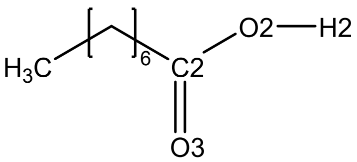

#	Atoms							
#	X	Y	Z	M	Q	sigma	epsilon	Number
#		(A)		(u)	(e)	(A)	(kJ/M)	
C	21.2641	-11.6771	0.0032	12.011	-0.419	3.87541	0.23012	1
C	20.0101	-12.5331	-0.0789	12.011	-0.276	3.87541	0.23012	2
H	21.9583	-11.9194	-0.8078	1.007947	0.1462	2.35197	0.092048	3
H	21.7814	-11.8299	0.9558	1.007947	0.1462	2.35197	0.092048	4
H	21.0053	-10.6167	-0.0765	1.007947	0.1462	2.35197	0.092048	5
C	20.3413	-14.0214	0.0257	12.011	-0.277	3.87541	0.23012	6
H	19.4983	-12.3312	-1.0271	1.007947	0.14682	2.35197	0.092048	7
H	19.3252	-12.2458	0.7273	1.007947	0.1462	2.35197	0.092048	8
C	19.0782	-14.8808	-0.0505	12.011	-0.277	3.87541	0.23012	9
H	20.8612	-14.2165	0.9716	1.007947	0.1462	2.35197	0.092048	10
H	21.0265	-14.3026	-0.7833	1.007947	0.14682	2.35197	0.092048	11
C	19.4143	-16.3699	0.0449	12.011	-0.277	3.87541	0.23012	12

H	18.5543	-14.6822	-0.9934	1.007947	0.14682	2.35197	0.092048	13
H	18.3963	-14.6046	0.763	1.007947	0.1462	2.35197	0.092048	14
C	18.1535	-17.2324	-0.0215	12.011	-0.277	3.87541	0.23012	15
H	19.9445	-16.5675	0.9845	1.007947	0.1462	2.35197	0.092048	16
H	20.0914	-16.6459	-0.7726	1.007947	0.1462	2.35197	0.092048	17
C	18.4937	-18.7191	0.0636	12.011	-0.2777	3.87541	0.23012	18
H	17.6145	-17.0303	-0.9556	1.007947	0.1462	2.35197	0.092048	19
H	17.476	-16.9607	0.7978	1.007947	0.14682	2.35197	0.092048	20
C2	17.2598	-19.5778	0.0032	12.011	0.0928	3.56359	0.46024	21
H	19.0106	-18.9299	1.0063	1.007947	0.1462	2.35197	0.092048	22
H	19.1458	-18.9977	-0.7716	1.007947	0.1462	2.35197	0.092048	23
O3	16.0996	-19.2111	-0.0623	15.9994	-0.27231	3.02905	0.50208	24
O2	17.5621	-20.8911	0.0345	15.9994	-0.20069	3.15378	0.636386	25
H2	16.6895	-21.3357	-0.0076	1.007947	0.26479	0.40001	0.192464	26

#	Bonds			
	25			
#	N1	N2	R-eqv	Force
0	1	2	1.508	1282.11
0	1	3	1.093	1435.07
0	1	4	1.093	1435.07
0	1	5	1.093	1435.07
0	2	6	1.508	1282.11
0	2	7	1.093	1435.07
0	2	8	1.093	1435.07
0	6	9	1.508	1282.11
0	6	10	1.093	1435.07
0	6	11	1.093	1435.07
0	9	12	1.508	1282.11
0	9	13	1.093	1435.07
0	9	14	1.093	1435.07
0	12	15	1.508	1282.11
0	12	16	1.093	1435.07
0	12	17	1.093	1435.07
0	15	18	1.508	1282.11
0	15	19	1.093	1435.07
0	15	20	1.093	1435.07
0	18	21	1.492	1261.64
0	18	22	1.093	1435.07
0	18	23	1.093	1435.07
0	21	24	1.222	3899.33
0	21	25	1.355	1746.72
0	25	26	0.981	2229.09
#	Angles			
	46			
#N1	N2	N3	A-eqv	Force
2	1	3	110.55	383
2	1	4	110.55	383
2	1	5	110.55	383
3	1	4	108.84	310.74
3	1	5	108.84	310.74
4	1	5	108.84	310.74
1	2	6	109.61	512.48
1	2	7	110.55	383
1	2	8	110.55	383
6	2	7	110.55	383
6	2	8	110.55	383
7	2	8	108.84	310.74
2	6	9	109.61	512.48
2	6	10	110.55	383
2	6	11	110.55	383
9	6	10	110.55	383
9	6	11	110.55	383
10	6	11	108.84	310.74
6	9	12	109.61	512.48
6	9	13	110.55	383

6	9	14	110.55	383		
12	9	13	110.55	383		
12	9	14	110.55	383		
13	9	14	108.84	310.74		
9	12	15	109.61	512.48		
9	12	16	110.55	383		
9	12	17	110.55	383		
15	12	16	110.55	383		
15	12	17	110.55	383		
16	12	17	108.84	310.74		
12	15	18	109.61	512.48		
12	15	19	110.55	383		
12	15	20	110.55	383		
18	15	19	110.55	383		
18	15	20	110.55	383		
19	15	20	108.84	310.74		
15	18	21	107.52	467.91		
15	18	22	110.55	383		
15	18	23	110.55	383		
21	18	22	108.39	391.44		
21	18	23	108.39	391.44		
22	18	23	108.84	310.74		
18	21	24	124.41	564.87		
18	21	25	109.72	628.1		
24	21	25	124.43	695.55		
21	25	26	111.95	351.09		
#	Dihedrals					
	182					
#N1	N2	N3	N4	A-eqv	Force	n
1	2	6	9	0	0.2134	1
1	2	6	9	180	1.4267	2
1	2	6	9	0	0.6945	3
1	2	6	10	0	1.3389	1
1	2	6	10	180	-1.318	2
1	2	6	10	0	0.5523	3
1	2	6	11	0	1.3389	1
1	2	6	11	180	-1.318	2
1	2	6	11	0	0.5523	3
2	6	9	12	0	0.2134	1
2	6	9	12	180	1.4267	2
2	6	9	12	0	0.6945	3
2	6	9	13	0	1.3389	1
2	6	9	13	180	-1.318	2
2	6	9	13	0	0.5523	3
2	6	9	14	0	1.3389	1
2	6	9	14	180	-1.318	2
2	6	9	14	0	0.5523	3
3	1	2	6	0	1.3389	1
3	1	2	6	180	-1.318	2
3	1	2	6	0	0.5523	3
3	1	2	7	0	0.5941	1
3	1	2	7	180	-2.8995	2
3	1	2	7	0	0.6569	3
3	1	2	8	0	0.5941	1
3	1	2	8	180	-2.8995	2
3	1	2	8	0	0.6569	3
4	1	2	6	0	1.3389	1
4	1	2	6	180	-1.318	2
4	1	2	6	0	0.5523	3
4	1	2	7	0	0.5941	1
4	1	2	7	180	-2.8995	2
4	1	2	7	0	0.6569	3
4	1	2	8	0	0.5941	1
4	1	2	8	180	-2.8995	2
4	1	2	8	0	0.6569	3

5	1	2	6	0	1.3389	1
5	1	2	6	180	-1.318	2
5	1	2	6	0	0.5523	3
5	1	2	7	0	0.5941	1
5	1	2	7	180	-2.8995	2
5	1	2	7	0	0.6569	3
5	1	2	8	0	0.5941	1
5	1	2	8	180	-2.8995	2
5	1	2	8	0	0.6569	3
6	9	12	15	0	0.2134	1
6	9	12	15	180	1.4267	2
6	9	12	15	0	0.6945	3
6	9	12	16	0	1.3389	1
6	9	12	16	180	-1.318	2
6	9	12	16	0	0.5523	3
6	9	12	17	0	1.3389	1
6	9	12	17	180	-1.318	2
6	9	12	17	0	0.5523	3
7	2	6	9	0	1.3389	1
7	2	6	9	180	-1.318	2
7	2	6	9	0	0.5523	3
7	2	6	10	0	0.5941	1
7	2	6	10	180	-2.8995	2
7	2	6	10	0	0.6569	3
7	2	6	11	0	0.5941	1
7	2	6	11	180	-2.8995	2
7	2	6	11	0	0.6569	3
8	2	6	9	0	1.3389	1
8	2	6	9	180	-1.318	2
8	2	6	9	0	0.5523	3
8	2	6	10	0	0.5941	1
8	2	6	10	180	-2.8995	2
8	2	6	10	0	0.6569	3
8	2	6	11	0	0.5941	1
8	2	6	11	180	-2.8995	2
8	2	6	11	0	0.6569	3
9	12	15	18	0	0.2134	1
9	12	15	18	180	1.4267	2
9	12	15	18	0	0.6945	3
9	12	15	19	0	1.3389	1
9	12	15	19	180	-1.318	2
9	12	15	19	0	0.5523	3
9	12	15	20	0	1.3389	1
9	12	15	20	180	-1.318	2
9	12	15	20	0	0.5523	3
10	6	9	12	0	1.3389	1
10	6	9	12	180	-1.318	2
10	6	9	12	0	0.5523	3
10	6	9	13	0	0.5941	1
10	6	9	13	180	-2.8995	2
10	6	9	13	0	0.6569	3
10	6	9	14	0	0.5941	1
10	6	9	14	180	-2.8995	2
10	6	9	14	0	0.6569	3
11	6	9	12	0	1.3389	1
11	6	9	12	180	-1.318	2
11	6	9	12	0	0.5523	3
11	6	9	13	0	0.5941	1
11	6	9	13	180	-2.8995	2
11	6	9	13	0	0.6569	3
11	6	9	14	0	0.5941	1
11	6	9	14	180	-2.8995	2
11	6	9	14	0	0.6569	3
12	15	18	21	0	0.1381	1
12	15	18	21	180	-0.3264	2

12	15	18	21	0	0.2971	3
12	15	18	22	0	1.3389	1
12	15	18	22	180	-1.318	2
12	15	18	22	0	0.5523	3
12	15	18	23	0	1.3389	1
12	15	18	23	180	-1.318	2
12	15	18	23	0	0.5523	3
13	9	12	15	0	1.3389	1
13	9	12	15	180	-1.318	2
13	9	12	15	0	0.5523	3
13	9	12	16	0	0.5941	1
13	9	12	16	180	-2.8995	2
13	9	12	16	0	0.6569	3
13	9	12	17	0	0.5941	1
13	9	12	17	180	-2.8995	2
13	9	12	17	0	0.6569	3
14	9	12	15	0	1.3389	1
14	9	12	15	180	-1.318	2
14	9	12	15	0	0.5523	3
14	9	12	16	0	0.5941	1
14	9	12	16	180	-2.8995	2
14	9	12	16	0	0.6569	3
14	9	12	17	0	0.5941	1
14	9	12	17	180	-2.8995	2
14	9	12	17	0	0.6569	3
15	18	21	24	0	1.7238	1
15	18	21	24	180	0.2929	2
15	18	21	24	0	0.682	3
15	18	21	25	0	-0.2469	1
15	18	21	25	180	-0.6987	2
15	18	21	25	0	0.4226	3
16	12	15	18	0	1.3389	1
16	12	15	18	180	-1.318	2
16	12	15	18	0	0.5523	3
16	12	15	19	0	0.5941	1
16	12	15	19	180	-2.8995	2
16	12	15	19	0	0.6569	3
16	12	15	20	0	0.5941	1
16	12	15	20	180	-2.8995	2
16	12	15	20	0	0.6569	3
17	12	15	18	0	1.3389	1
17	12	15	18	180	-1.318	2
17	12	15	18	0	0.5523	3
17	12	15	19	0	0.5941	1
17	12	15	19	180	-2.8995	2
17	12	15	19	0	0.6569	3
17	12	15	20	0	0.5941	1
17	12	15	20	180	-2.8995	2
17	12	15	20	0	0.6569	3
18	21	25	26	0	-2.4393	1
18	21	25	26	180	10.6232	2
18	21	25	26	0	-1.1422	3
19	15	18	21	0	-0.5356	1
19	15	18	21	180	0.1213	2
19	15	18	22	0	0.5941	1
19	15	18	22	180	-2.8995	2
19	15	18	22	0	0.6569	3
19	15	18	23	0	0.5941	1
19	15	18	23	180	-2.8995	2
19	15	18	23	0	0.6569	3
20	15	18	21	0	-0.5356	1
20	15	18	21	180	0.1213	2
20	15	18	22	0	0.5941	1
20	15	18	22	180	-2.8995	2
20	15	18	22	0	0.6569	3

20	15	18	23	0	0.5941	1
20	15	18	23	180	-2.8995	2
20	15	18	23	0	0.6569	3
22	18	21	24	0	1.3807	1
22	18	21	24	180	-2.9455	2
22	18	21	24	0	0.6443	3
22	18	21	25	180	-1.3054	2
22	18	21	25	0	0.6904	3
23	18	21	24	0	1.3807	1
23	18	21	24	180	-2.9455	2
23	18	21	24	0	0.6443	3
23	18	21	25	180	-1.3054	2
23	18	21	25	0	0.6904	3
24	21	25	26	0	3.4769	1
24	21	25	26	180	12.87	2
24	21	25	26	0	-0.1213	3

improper

	15				
1	3	2	4	0	0
1	3	2	5	0	0
2	6	1	7	0	0
2	6	1	8	0	0
6	9	2	10	0	0
6	9	2	11	0	0
9	12	6	13	0	0
9	12	6	14	0	0
12	15	9	16	0	0
12	15	9	17	0	0
15	18	12	19	0	0
15	18	12	20	0	0
18	21	15	22	0	0
18	21	15	23	0	0
21	25	18	24	0	84.9101

[C10AC]

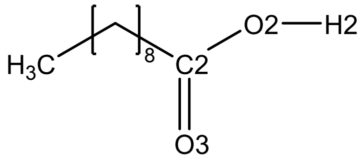

#	Atoms							
#	X	Y	Z	M	Q	sigma	epsilon	Number
#		(A)		(u)	(e)	(A)	(kJ/M)	
C	2.88781	1.20937	0.03317	12.01100	-0.35999	3.87541	0.23012	1
C	4.13461	2.09756	0.06791	12.01100	0.24019	3.87541	0.23012	2
H	2.91576	0.52095	-0.81831	1.00795	0.07728	2.35197	0.09205	3
H	1.97357	1.80391	-0.05101	1.00795	0.08329	2.35197	0.09205	4
H	2.80790	0.60421	0.94258	1.00795	0.07590	2.35197	0.09205	5
C	5.44141	1.30406	0.18150	12.01100	-0.00305	3.87541	0.23012	6
H	4.06252	2.79602	0.91145	1.00795	-0.04021	2.35197	0.09205	7
H	4.16764	2.71751	-0.83721	1.00795	-0.04059	2.35197	0.09205	8
C	6.69478	2.18453	0.22539	12.01100	-0.07570	3.87541	0.23012	9
H	5.51553	0.60854	-0.66593	1.00795	-0.00274	2.35197	0.09205	10
H	5.40592	0.67836	1.08404	1.00795	-0.00101	2.35197	0.09205	11
C	8.00190	1.39117	0.33526	12.01100	0.12001	3.87541	0.23012	12
H	6.62041	2.87755	1.07451	1.00795	0.00508	2.35197	0.09205	13
H	6.72795	2.81227	-0.67556	1.00795	0.00318	2.35197	0.09205	14
C	9.25345	2.27431	0.38584	12.01100	0.01066	3.87541	0.23012	15
H	8.07887	0.70143	-0.51636	1.00795	-0.02753	2.35197	0.09205	16
H	7.96738	0.76025	1.23400	1.00795	-0.02938	2.35197	0.09205	17
C	10.56079	1.48154	0.49569	12.01100	-0.06582	3.87541	0.23012	18
H	9.17681	2.96333	1.23770	1.00795	-0.00339	2.35197	0.09205	19

H	9.28854	2.90579	-0.51214	1.00795	-0.00249	2.35197	0.09205	20
C	11.80631	2.37236	0.54944	12.01100	0.04125	3.87541	0.23012	21
H	10.63909	0.79377	-0.35751	1.00795	0.01397	2.35197	0.09205	22
H	10.52483	0.84849	1.39303	1.00795	0.01442	2.35197	0.09205	23
C	13.09951	1.56561	0.66421	12.01100	-0.21337	3.87541	0.23012	24
H	11.73462	3.06105	1.39828	1.00795	0.02255	2.35197	0.09205	25
H	11.85201	3.00312	-0.34495	1.00795	0.02160	2.35197	0.09205	26
C2	14.34666	2.41555	0.72217	12.01100	0.78799	3.56359	0.46024	27
H	13.21325	0.87422	-0.17964	1.00795	0.08575	2.35197	0.09205	28
H	13.09364	0.93182	1.55931	1.00795	0.08494	2.35197	0.09205	29
O3	14.39668	3.61935	0.68011	15.99940	-0.59654	3.02905	0.50208	30
O2	15.46420	1.64814	0.82963	15.99940	-0.67011	3.15378	0.63639	31
H2	16.22159	2.25208	0.86185	1.00795	0.44383	0.40001	0.19246	32

#	Bonds			
	31			
#	N1	N2	R-eqv	Force
0	8	2	1.093	1435.0745
0	3	1	1.093	1435.0745
0	14	9	1.093	1435.0745
0	10	6	1.093	1435.0745
0	16	12	1.093	1435.0745
0	20	15	1.093	1435.0745
0	22	18	1.093	1435.0745
0	26	21	1.093	1435.0745
0	28	24	1.093	1435.0745
0	4	1	1.093	1435.0745
0	1	2	1.508	1282.1115
0	1	5	1.093	1435.0745
0	2	6	1.508	1282.1115
0	2	7	1.093	1435.0745
0	6	9	1.508	1282.1115
0	6	11	1.093	1435.0745
0	9	12	1.508	1282.1115
0	9	13	1.093	1435.0745
0	12	15	1.508	1282.1115
0	12	17	1.093	1435.0745
0	15	18	1.508	1282.1115
0	15	19	1.093	1435.0745
0	18	21	1.508	1282.1115
0	18	23	1.093	1435.0745
0	21	24	1.508	1282.1115
0	21	25	1.093	1435.0745
0	24	27	1.492	1261.639
0	24	29	1.093	1435.0745
0	30	27	1.222	3899.333
0	27	31	1.355	1746.7195
0	31	32	0.981	2229.093
#	Angles			
	58			
#N1	N2	N3	A-eqv	Force
2	1	3	110.549	383
2	1	4	110.549	383
2	1	5	110.549	383
3	1	4	108.836	310.74
3	1	5	108.836	310.74
4	1	5	108.836	310.74
1	2	6	109.608	512.48
1	2	7	110.549	383
1	2	8	110.549	383
6	2	7	110.549	383
6	2	8	110.549	383
7	2	8	108.836	310.74
2	6	9	109.608	512.48
2	6	10	110.549	383
2	6	11	110.549	383

9	6	10	110.549	383		
9	6	11	110.549	383		
10	6	11	108.836	310.74		
6	9	12	109.608	512.48		
6	9	13	110.549	383		
6	9	14	110.549	383		
12	9	13	110.549	383		
12	9	14	110.549	383		
13	9	14	108.836	310.74		
9	12	15	109.608	512.48		
9	12	16	110.549	383		
9	12	17	110.549	383		
15	12	16	110.549	383		
15	12	17	110.549	383		
16	12	17	108.836	310.74		
12	15	18	109.608	512.48		
12	15	19	110.549	383		
12	15	20	110.549	383		
18	15	19	110.549	383		
18	15	20	110.549	383		
19	15	20	108.836	310.74		
15	18	21	109.608	512.48		
15	18	22	110.549	383		
15	18	23	110.549	383		
21	18	22	110.549	383		
21	18	23	110.549	383		
22	18	23	108.836	310.74		
18	21	24	109.608	512.48		
18	21	25	110.549	383		
18	21	26	110.549	383		
24	21	25	110.549	383		
24	21	26	110.549	383		
25	21	26	108.836	310.74		
21	24	27	107.517	467.91		
21	24	28	110.549	383		
21	24	29	110.549	383		
27	24	28	108.385	391.44		
27	24	29	108.385	391.44		
28	24	29	108.836	310.74		
24	27	30	124.41	564.87		
24	27	31	109.716	628.1		
30	27	31	124.425	695.55		
27	31	32	111.948	351.09		
#	Dihedrals					
	236					
#N1	N2	N3	N4	A-eqv	Force	n
1	2	6	9	0	0.2134	1
1	2	6	9	180	1.4267	2
1	2	6	9	0	0.6945	3
1	2	6	10	0	1.3389	1
1	2	6	10	180	-1.318	2
1	2	6	10	0	0.5523	3
1	2	6	11	0	1.3389	1
1	2	6	11	180	-1.318	2
1	2	6	11	0	0.5523	3
2	6	9	12	0	0.2134	1
2	6	9	12	180	1.4267	2
2	6	9	12	0	0.6945	3
2	6	9	13	0	1.3389	1
2	6	9	13	180	-1.318	2
2	6	9	13	0	0.5523	3
2	6	9	14	0	1.3389	1
2	6	9	14	180	-1.318	2
2	6	9	14	0	0.5523	3
3	1	2	6	0	1.3389	1

3	1	2	6	180	-1.318	2
3	1	2	6	0	0.5523	3
3	1	2	7	0	0.5941	1
3	1	2	7	180	-2.8995	2
3	1	2	7	0	0.6569	3
3	1	2	8	0	0.5941	1
3	1	2	8	180	-2.8995	2
3	1	2	8	0	0.6569	3
4	1	2	6	0	1.3389	1
4	1	2	6	180	-1.318	2
4	1	2	6	0	0.5523	3
4	1	2	7	0	0.5941	1
4	1	2	7	180	-2.8995	2
4	1	2	7	0	0.6569	3
4	1	2	8	0	0.5941	1
4	1	2	8	180	-2.8995	2
4	1	2	8	0	0.6569	3
5	1	2	6	0	1.3389	1
5	1	2	6	180	-1.318	2
5	1	2	6	0	0.5523	3
5	1	2	7	0	0.5941	1
5	1	2	7	180	-2.8995	2
5	1	2	7	0	0.6569	3
5	1	2	8	0	0.5941	1
5	1	2	8	180	-2.8995	2
5	1	2	8	0	0.6569	3
6	9	12	15	0	0.2134	1
6	9	12	15	180	1.4267	2
6	9	12	15	0	0.6945	3
6	9	12	16	0	1.3389	1
6	9	12	16	180	-1.318	2
6	9	12	16	0	0.5523	3
6	9	12	17	0	1.3389	1
6	9	12	17	180	-1.318	2
6	9	12	17	0	0.5523	3
7	2	6	9	0	1.3389	1
7	2	6	9	180	-1.318	2
7	2	6	9	0	0.5523	3
7	2	6	10	0	0.5941	1
7	2	6	10	180	-2.8995	2
7	2	6	10	0	0.6569	3
7	2	6	11	0	0.5941	1
7	2	6	11	180	-2.8995	2
7	2	6	11	0	0.6569	3
8	2	6	9	0	1.3389	1
8	2	6	9	180	-1.318	2
8	2	6	9	0	0.5523	3
8	2	6	10	0	0.5941	1
8	2	6	10	180	-2.8995	2
8	2	6	10	0	0.6569	3
8	2	6	11	0	0.5941	1
8	2	6	11	180	-2.8995	2
8	2	6	11	0	0.6569	3
9	12	15	18	0	0.2134	1
9	12	15	18	180	1.4267	2
9	12	15	18	0	0.6945	3
9	12	15	19	0	1.3389	1
9	12	15	19	180	-1.318	2
9	12	15	19	0	0.5523	3
9	12	15	20	0	1.3389	1
9	12	15	20	180	-1.318	2
9	12	15	20	0	0.5523	3
10	6	9	12	0	1.3389	1
10	6	9	12	180	-1.318	2
10	6	9	12	0	0.5523	3

10	6	9	13	0	0.5941	1
10	6	9	13	180	-2.8995	2
10	6	9	13	0	0.6569	3
10	6	9	14	0	0.5941	1
10	6	9	14	180	-2.8995	2
10	6	9	14	0	0.6569	3
11	6	9	12	0	1.3389	1
11	6	9	12	180	-1.318	2
11	6	9	12	0	0.5523	3
11	6	9	13	0	0.5941	1
11	6	9	13	180	-2.8995	2
11	6	9	13	0	0.6569	3
11	6	9	14	0	0.5941	1
11	6	9	14	180	-2.8995	2
11	6	9	14	0	0.6569	3
12	15	18	21	0	0.2134	1
12	15	18	21	180	1.4267	2
12	15	18	21	0	0.6945	3
12	15	18	22	0	1.3389	1
12	15	18	22	180	-1.318	2
12	15	18	22	0	0.5523	3
12	15	18	23	0	1.3389	1
12	15	18	23	180	-1.318	2
12	15	18	23	0	0.5523	3
13	9	12	15	0	1.3389	1
13	9	12	15	180	-1.318	2
13	9	12	15	0	0.5523	3
13	9	12	16	0	0.5941	1
13	9	12	16	180	-2.8995	2
13	9	12	16	0	0.6569	3
13	9	12	17	0	0.5941	1
13	9	12	17	180	-2.8995	2
13	9	12	17	0	0.6569	3
14	9	12	15	0	1.3389	1
14	9	12	15	180	-1.318	2
14	9	12	15	0	0.5523	3
14	9	12	16	0	0.5941	1
14	9	12	16	180	-2.8995	2
14	9	12	16	0	0.6569	3
14	9	12	17	0	0.5941	1
14	9	12	17	180	-2.8995	2
14	9	12	17	0	0.6569	3
15	18	21	24	0	0.2134	1
15	18	21	24	180	1.4267	2
15	18	21	24	0	0.6945	3
15	18	21	25	0	1.3389	1
15	18	21	25	180	-1.318	2
15	18	21	25	0	0.5523	3
15	18	21	26	0	1.3389	1
15	18	21	26	180	-1.318	2
15	18	21	26	0	0.5523	3
16	12	15	18	0	1.3389	1
16	12	15	18	180	-1.318	2
16	12	15	18	0	0.5523	3
16	12	15	19	0	0.5941	1
16	12	15	19	180	-2.8995	2
16	12	15	19	0	0.6569	3
16	12	15	20	0	0.5941	1
16	12	15	20	180	-2.8995	2
16	12	15	20	0	0.6569	3
17	12	15	18	0	1.3389	1
17	12	15	18	180	-1.318	2
17	12	15	18	0	0.5523	3
17	12	15	19	0	0.5941	1
17	12	15	19	180	-2.8995	2

17	12	15	19	0	0.6569	3
17	12	15	20	0	0.5941	1
17	12	15	20	180	-2.8995	2
17	12	15	20	0	0.6569	3
18	21	24	27	0	0.1381	1
18	21	24	27	180	-0.3264	2
18	21	24	27	0	0.2971	3
18	21	24	28	0	1.3389	1
18	21	24	28	180	-1.318	2
18	21	24	28	0	0.5523	3
18	21	24	29	0	1.3389	1
18	21	24	29	180	-1.318	2
18	21	24	29	0	0.5523	3
19	15	18	21	0	1.3389	1
19	15	18	21	180	-1.318	2
19	15	18	21	0	0.5523	3
19	15	18	22	0	0.5941	1
19	15	18	22	180	-2.8995	2
19	15	18	22	0	0.6569	3
19	15	18	23	0	0.5941	1
19	15	18	23	180	-2.8995	2
19	15	18	23	0	0.6569	3
20	15	18	21	0	1.3389	1
20	15	18	21	180	-1.318	2
20	15	18	21	0	0.5523	3
20	15	18	22	0	0.5941	1
20	15	18	22	180	-2.8995	2
20	15	18	22	0	0.6569	3
20	15	18	23	0	0.5941	1
20	15	18	23	180	-2.8995	2
20	15	18	23	0	0.6569	3
21	24	27	30	0	1.7238	1
21	24	27	30	180	0.2929	2
21	24	27	30	0	0.682	3
21	24	27	31	0	-0.2469	1
21	24	27	31	180	-0.6987	2
21	24	27	31	0	0.4226	3
22	18	21	24	0	1.3389	1
22	18	21	24	180	-1.318	2
22	18	21	24	0	0.5523	3
22	18	21	25	0	0.5941	1
22	18	21	25	180	-2.8995	2
22	18	21	25	0	0.6569	3
22	18	21	26	0	0.5941	1
22	18	21	26	180	-2.8995	2
22	18	21	26	0	0.6569	3
23	18	21	24	0	1.3389	1
23	18	21	24	180	-1.318	2
23	18	21	24	0	0.5523	3
23	18	21	25	0	0.5941	1
23	18	21	25	180	-2.8995	2
23	18	21	25	0	0.6569	3
23	18	21	26	0	0.5941	1
23	18	21	26	180	-2.8995	2
23	18	21	26	0	0.6569	3
24	27	31	32	0	-2.4393	1
24	27	31	32	180	10.6232	2
24	27	31	32	0	-1.1422	3
25	21	24	27	0	-0.5356	1
25	21	24	27	180	0.1213	2
25	21	24	28	0	0.5941	1
25	21	24	28	180	-2.8995	2
25	21	24	28	0	0.6569	3
25	21	24	29	0	0.5941	1
25	21	24	29	180	-2.8995	2

25	21	24	29	0	0.6569	3
26	21	24	27	0	-0.5356	1
26	21	24	27	180	0.1213	2
26	21	24	28	0	0.5941	1
26	21	24	28	180	-2.8995	2
26	21	24	28	0	0.6569	3
26	21	24	29	0	0.5941	1
26	21	24	29	180	-2.8995	2
26	21	24	29	0	0.6569	3
28	24	27	30	0	1.3807	1
28	24	27	30	180	-2.9455	2
28	24	27	30	0	0.6443	3
28	24	27	31	180	-1.3054	2
28	24	27	31	0	0.6904	3
29	24	27	30	0	1.3807	1
29	24	27	30	180	-2.9455	2
29	24	27	30	0	0.6443	3
29	24	27	31	180	-1.3054	2
29	24	27	31	0	0.6904	3
30	27	31	32	0	3.4769	1
30	27	31	32	180	12.87	2
30	27	31	32	0	-0.1213	3
improper						
	19					
1	3	2	4	0	0	
1	3	2	5	0	0	
2	6	1	8	0	0	
2	8	1	7	0	0	
6	9	2	10	0	0	
6	10	2	11	0	0	
9	12	6	14	0	0	
9	14	6	13	0	0	
12	15	9	16	0	0	
12	16	9	17	0	0	
15	18	12	20	0	0	
15	20	12	19	0	0	
18	21	15	22	0	0	
18	22	15	23	0	0	
21	24	18	26	0	0	
21	26	18	25	0	0	
24	27	21	28	0	0	
24	28	21	29	0	0	
27	31	24	30	0	84.9101	

[C10]

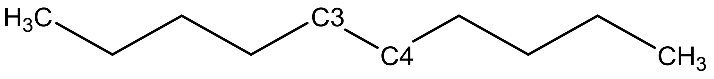

#	Atoms							
#	X	Y	Z	M	Q	sigma	epsilon	Number
#		(A)		(u)	(e)	(A)	(kJ/M)	
C	-1.452601	1.560314	-0.009639	12.011000	-0.402985	3.581410	0.234304	1
C	-0.174529	0.735448	-0.002968	12.011000	0.232440	3.581410	0.234304	2
H	-2.336055	0.918522	0.010544	1.007947	0.095316	2.351970	0.092048	3
H	-1.512768	2.188832	-0.901646	1.007947	0.087219	2.351970	0.092048	4
H	-1.499338	2.223368	0.857708	1.007947	0.086740	2.351970	0.092048	5
C	1.092529	1.578749	-0.014819	12.011000	0.038184	3.581410	0.234304	6
H	-0.168416	0.066351	-0.869656	1.007947	-0.036717	2.351970	0.092048	7
H	-0.164853	0.087631	0.879573	1.007947	-0.036656	2.351970	0.092048	8
C	2.367990	0.749240	0.002977	12.011000	-0.107130	3.581410	0.234304	9
H	1.086125	2.252364	0.849676	1.007947	-0.009693	2.351970	0.092048	10
H	1.090667	2.222257	-0.902040	1.007947	-0.008795	2.351970	0.092048	11
C3	3.641769	1.581372	-0.000028	12.011000	0.103277	3.581410	0.234304	12

H	2.372741	0.079302	-0.864214	1.007947	0.004676	2.351970	0.092048	13
H	2.362323	0.102237	0.887343	1.007947	0.005369	2.351970	0.092048	14
C4	4.914161	0.746543	0.017834	12.011000	0.102457	3.581410	0.234304	15
H	3.637697	2.250978	0.867374	1.007947	-0.026224	2.351970	0.092048	16
H	3.648057	2.228448	-0.884310	1.007947	-0.025553	2.351970	0.092048	17
C	6.187710	1.579023	0.009060	12.011000	-0.105234	3.581410	0.234304	18
H	4.916417	0.074734	-0.847828	1.007947	-0.025318	2.351970	0.092048	19
H	4.909769	0.101741	0.903834	1.007947	-0.025207	2.351970	0.092048	20
C	7.463774	0.750244	0.014701	12.011000	0.027159	3.581410	0.234304	21
H	6.188682	2.246187	0.878447	1.007947	0.006827	2.351970	0.092048	22
H	6.186899	2.228839	-0.873203	1.007947	0.006252	2.351970	0.092048	23
C	8.729877	1.594662	-0.014523	12.011000	0.239980	3.581410	0.234304	24
H	7.461130	0.074430	-0.848050	1.007947	-0.008011	2.351970	0.092048	25
H	7.475772	0.109048	0.903556	1.007947	-0.007964	2.351970	0.092048	26
C	10.008923	0.771351	-0.026731	12.011000	-0.394350	3.581410	0.234304	27
H	8.735522	2.263011	0.852800	1.007947	-0.039135	2.351970	0.092048	28
H	8.706739	2.243225	-0.896191	1.007947	-0.038499	2.351970	0.092048	29
H	10.088891	0.152260	0.870263	1.007947	0.084438	2.351970	0.092048	30
H	10.890844	1.413887	-0.072853	1.007947	0.092321	2.351970	0.092048	31
H	10.038551	0.099296	-0.887900	1.007947	0.084814	2.351970	0.092048	32
#	Bonds							
	31							
#	N1	N2	R-eqv	Force				
0	1	2	1.508	1282.11				
0	1	3	1.093	1435.07				
0	1	4	1.093	1435.07				
0	1	5	1.093	1435.07				
0	2	7	1.093	1435.07				
0	2	8	1.093	1435.07				
0	2	6	1.508	1282.11				
0	6	9	1.508	1282.11				
0	6	10	1.093	1435.07				
0	6	11	1.093	1435.07				
0	9	13	1.093	1435.07				
0	9	14	1.093	1435.07				
0	9	12	1.508	1282.11				
0	12	15	1.508	1282.11				
0	12	16	1.093	1435.07				
0	12	17	1.093	1435.07				
0	15	19	1.093	1435.07				
0	15	20	1.093	1435.07				
0	15	18	1.508	1282.11				
0	18	21	1.508	1282.11				
0	18	22	1.093	1435.07				
0	18	23	1.093	1435.07				
0	21	25	1.093	1435.07				
0	21	26	1.093	1435.07				
0	21	24	1.508	1282.11				
0	24	27	1.508	1282.11				
0	24	28	1.093	1435.07				
0	24	29	1.093	1435.07				
0	27	30	1.093	1435.07				
0	27	31	1.093	1435.07				
0	27	32	1.093	1435.07				
#	Angles							
	60							
#N1	N2	N3	A-eqv	Force				
2	1	3	110.549	383				
2	1	4	110.549	383				
2	1	5	110.549	383				
3	1	4	108.836	310.74				
3	1	5	108.836	310.74				
4	1	5	108.836	310.74				
1	2	6	109.608	512.48				
1	2	7	110.549	383				

1	2	8	110.549	383		
6	2	7	110.549	383		
6	2	8	110.549	383		
7	2	8	108.836	310.74		
2	6	9	109.608	512.48		
2	6	10	110.549	383		
2	6	11	110.549	383		
9	6	10	110.549	383		
9	6	11	110.549	383		
10	6	11	108.836	310.74		
6	9	12	109.608	512.48		
6	9	13	110.549	383		
6	9	14	110.549	383		
12	9	13	110.549	383		
12	9	14	110.549	383		
13	9	14	108.836	310.74		
9	12	15	109.608	512.48		
9	12	16	110.549	383		
9	12	17	110.549	383		
15	12	16	110.549	383		
15	12	17	110.549	383		
16	12	17	108.836	310.74		
12	15	18	109.608	512.48		
12	15	19	110.549	383		
12	15	20	110.549	383		
18	15	19	110.549	383		
18	15	20	110.549	383		
19	15	20	108.836	310.74		
15	18	21	109.608	512.48		
15	18	22	110.549	383		
15	18	23	110.549	383		
21	18	22	110.549	383		
21	18	23	110.549	383		
22	18	23	108.836	310.74		
18	21	24	109.608	512.48		
18	21	25	110.549	383		
18	21	26	110.549	383		
24	21	25	110.549	383		
24	21	26	110.549	383		
25	21	26	108.836	310.74		
21	24	27	109.608	512.48		
21	24	28	110.549	383		
21	24	29	110.549	383		
27	24	28	110.549	383		
27	24	29	110.549	383		
28	24	29	108.836	310.74		
24	27	30	110.549	383		
24	27	31	110.549	383		
24	27	32	110.549	383		
30	27	31	108.836	310.74		
30	27	32	108.836	310.74		
31	27	32	108.836	310.74		
#	Dihedrals					
	243					
#N1	N2	N3	N4	A-eqv	Force	n
1	2	6	9	0	0.2134	1
1	2	6	9	180	1.4267	2
1	2	6	9	0	0.6945	3
1	2	6	10	0	1.3389	1
1	2	6	10	180	-1.318	2
1	2	6	10	0	0.5523	3
1	2	6	11	0	1.3389	1
1	2	6	11	180	-1.318	2
1	2	6	11	0	0.5523	3
2	6	9	12	0	0.2134	1

2	6	9	12	180	1.4267	2
2	6	9	12	0	0.6945	3
2	6	9	13	0	1.3389	1
2	6	9	13	180	-1.318	2
2	6	9	13	0	0.5523	3
2	6	9	14	0	1.3389	1
2	6	9	14	180	-1.318	2
2	6	9	14	0	0.5523	3
3	1	2	6	0	1.3389	1
3	1	2	6	180	-1.318	2
3	1	2	6	0	0.5523	3
3	1	2	7	0	0.5941	1
3	1	2	7	180	-2.8995	2
3	1	2	7	0	0.6569	3
3	1	2	8	0	0.5941	1
3	1	2	8	180	-2.8995	2
3	1	2	8	0	0.6569	3
4	1	2	6	0	1.3389	1
4	1	2	6	180	-1.318	2
4	1	2	6	0	0.5523	3
4	1	2	7	0	0.5941	1
4	1	2	7	180	-2.8995	2
4	1	2	7	0	0.6569	3
4	1	2	8	0	0.5941	1
4	1	2	8	180	-2.8995	2
4	1	2	8	0	0.6569	3
5	1	2	6	0	1.3389	1
5	1	2	6	180	-1.318	2
5	1	2	6	0	0.5523	3
5	1	2	7	0	0.5941	1
5	1	2	7	180	-2.8995	2
5	1	2	7	0	0.6569	3
5	1	2	8	0	0.5941	1
5	1	2	8	180	-2.8995	2
5	1	2	8	0	0.6569	3
6	9	12	15	0	0.2134	1
6	9	12	15	180	1.4267	2
6	9	12	15	0	0.6945	3
6	9	12	16	0	1.3389	1
6	9	12	16	180	-1.318	2
6	9	12	16	0	0.5523	3
6	9	12	17	0	1.3389	1
6	9	12	17	180	-1.318	2
6	9	12	17	0	0.5523	3
7	2	6	9	0	1.3389	1
7	2	6	9	180	-1.318	2
7	2	6	9	0	0.5523	3
7	2	6	10	0	0.5941	1
7	2	6	10	180	-2.8995	2
7	2	6	10	0	0.6569	3
7	2	6	11	0	0.5941	1
7	2	6	11	180	-2.8995	2
7	2	6	11	0	0.6569	3
8	2	6	9	0	1.3389	1
8	2	6	9	180	-1.318	2
8	2	6	9	0	0.5523	3
8	2	6	10	0	0.5941	1
8	2	6	10	180	-2.8995	2
8	2	6	10	0	0.6569	3
8	2	6	11	0	0.5941	1
8	2	6	11	180	-2.8995	2
8	2	6	11	0	0.6569	3
9	12	15	18	0	0.2134	1
9	12	15	18	180	1.4267	2
9	12	15	18	0	0.6945	3

9	12	15	19	0	1.3389	1
9	12	15	19	180	-1.318	2
9	12	15	19	0	0.5523	3
9	12	15	20	0	1.3389	1
9	12	15	20	180	-1.318	2
9	12	15	20	0	0.5523	3
10	6	9	12	0	1.3389	1
10	6	9	12	180	-1.318	2
10	6	9	12	0	0.5523	3
10	6	9	13	0	0.5941	1
10	6	9	13	180	-2.8995	2
10	6	9	13	0	0.6569	3
10	6	9	14	0	0.5941	1
10	6	9	14	180	-2.8995	2
10	6	9	14	0	0.6569	3
11	6	9	12	0	1.3389	1
11	6	9	12	180	-1.318	2
11	6	9	12	0	0.5523	3
11	6	9	13	0	0.5941	1
11	6	9	13	180	-2.8995	2
11	6	9	13	0	0.6569	3
11	6	9	14	0	0.5941	1
11	6	9	14	180	-2.8995	2
11	6	9	14	0	0.6569	3
12	15	18	21	0	0.2134	1
12	15	18	21	180	1.4267	2
12	15	18	21	0	0.6945	3
12	15	18	22	0	1.3389	1
12	15	18	22	180	-1.318	2
12	15	18	22	0	0.5523	3
12	15	18	23	0	1.3389	1
12	15	18	23	180	-1.318	2
12	15	18	23	0	0.5523	3
13	9	12	15	0	1.3389	1
13	9	12	15	180	-1.318	2
13	9	12	15	0	0.5523	3
13	9	12	16	0	0.5941	1
13	9	12	16	180	-2.8995	2
13	9	12	16	0	0.6569	3
13	9	12	17	0	0.5941	1
13	9	12	17	180	-2.8995	2
13	9	12	17	0	0.6569	3
14	9	12	15	0	1.3389	1
14	9	12	15	180	-1.318	2
14	9	12	15	0	0.5523	3
14	9	12	16	0	0.5941	1
14	9	12	16	180	-2.8995	2
14	9	12	16	0	0.6569	3
14	9	12	17	0	0.5941	1
14	9	12	17	180	-2.8995	2
14	9	12	17	0	0.6569	3
15	18	21	24	0	0.2134	1
15	18	21	24	180	1.4267	2
15	18	21	24	0	0.6945	3
15	18	21	25	0	1.3389	1
15	18	21	25	180	-1.318	2
15	18	21	25	0	0.5523	3
15	18	21	26	0	1.3389	1
15	18	21	26	180	-1.318	2
15	18	21	26	0	0.5523	3
16	12	15	18	0	1.3389	1
16	12	15	18	180	-1.318	2
16	12	15	18	0	0.5523	3
16	12	15	19	0	0.5941	1
16	12	15	19	180	-2.8995	2

16	12	15	19	0	0.6569	3
16	12	15	20	0	0.5941	1
16	12	15	20	180	-2.8995	2
16	12	15	20	0	0.6569	3
17	12	15	18	0	1.3389	1
17	12	15	18	180	-1.318	2
17	12	15	18	0	0.5523	3
17	12	15	19	0	0.5941	1
17	12	15	19	180	-2.8995	2
17	12	15	19	0	0.6569	3
17	12	15	20	0	0.5941	1
17	12	15	20	180	-2.8995	2
17	12	15	20	0	0.6569	3
18	21	24	27	0	0.2134	1
18	21	24	27	180	1.4267	2
18	21	24	27	0	0.6945	3
18	21	24	28	0	1.3389	1
18	21	24	28	180	-1.318	2
18	21	24	28	0	0.5523	3
18	21	24	29	0	1.3389	1
18	21	24	29	180	-1.318	2
18	21	24	29	0	0.5523	3
19	15	18	21	0	1.3389	1
19	15	18	21	180	-1.318	2
19	15	18	21	0	0.5523	3
19	15	18	22	0	0.5941	1
19	15	18	22	180	-2.8995	2
19	15	18	22	0	0.6569	3
19	15	18	23	0	0.5941	1
19	15	18	23	180	-2.8995	2
19	15	18	23	0	0.6569	3
20	15	18	21	0	1.3389	1
20	15	18	21	180	-1.318	2
20	15	18	21	0	0.5523	3
20	15	18	22	0	0.5941	1
20	15	18	22	180	-2.8995	2
20	15	18	22	0	0.6569	3
20	15	18	23	0	0.5941	1
20	15	18	23	180	-2.8995	2
20	15	18	23	0	0.6569	3
21	24	27	30	0	1.3389	1
21	24	27	30	180	-1.318	2
21	24	27	30	0	0.5523	3
21	24	27	31	0	1.3389	1
21	24	27	31	180	-1.318	2
21	24	27	31	0	0.5523	3
21	24	27	32	0	1.3389	1
21	24	27	32	180	-1.318	2
21	24	27	32	0	0.5523	3
22	18	21	24	0	1.3389	1
22	18	21	24	180	-1.318	2
22	18	21	24	0	0.5523	3
22	18	21	25	0	0.5941	1
22	18	21	25	180	-2.8995	2
22	18	21	25	0	0.6569	3
22	18	21	26	0	0.5941	1
22	18	21	26	180	-2.8995	2
22	18	21	26	0	0.6569	3
23	18	21	24	0	1.3389	1
23	18	21	24	180	-1.318	2
23	18	21	24	0	0.5523	3
23	18	21	25	0	0.5941	1
23	18	21	25	180	-2.8995	2
23	18	21	25	0	0.6569	3
23	18	21	26	0	0.5941	1

23	18	21	26	180	-2.8995	2
23	18	21	26	0	0.6569	3
25	21	24	27	0	1.3389	1
25	21	24	27	180	-1.318	2
25	21	24	27	0	0.5523	3
25	21	24	28	0	0.5941	1
25	21	24	28	180	-2.8995	2
25	21	24	28	0	0.6569	3
25	21	24	29	0	0.5941	1
25	21	24	29	180	-2.8995	2
25	21	24	29	0	0.6569	3
26	21	24	27	0	1.3389	1
26	21	24	27	180	-1.318	2
26	21	24	27	0	0.5523	3
26	21	24	28	0	0.5941	1
26	21	24	28	180	-2.8995	2
26	21	24	28	0	0.6569	3
26	21	24	29	0	0.5941	1
26	21	24	29	180	-2.8995	2
26	21	24	29	0	0.6569	3
28	24	27	30	0	0.5941	1
28	24	27	30	180	-2.8995	2
28	24	27	30	0	0.6569	3
28	24	27	31	0	0.5941	1
28	24	27	31	180	-2.8995	2
28	24	27	31	0	0.6569	3
28	24	27	32	0	0.5941	1
28	24	27	32	180	-2.8995	2
28	24	27	32	0	0.6569	3
29	24	27	30	0	0.5941	1
29	24	27	30	180	-2.8995	2
29	24	27	30	0	0.6569	3
29	24	27	31	0	0.5941	1
29	24	27	31	180	-2.8995	2
29	24	27	31	0	0.6569	3
29	24	27	32	0	0.5941	1
29	24	27	32	180	-2.8995	2
29	24	27	32	0	0.6569	3

Procedures for calculating excess and derived properties:

Experimental density data was considered for the calculation of excess molar volume, V^E:

$$V^E = \frac{\sum_{i=1}^{2} x_i M_i}{\rho} - \sum_{i=1}^{2} x_i \frac{M_i}{\rho_i} \tag{1}$$

Where M_i is the molar mass of the component i, ρ_i and ρ the densities of the pure compound i and for the corresponding mixture, respectively. Likewise, excess thermal expansion coefficient, α_p^E, was calculated as follows:

$$\alpha_p^E = \alpha_P - \sum_{i=1}^{2} \varphi_i \alpha_{p,i} \tag{2}$$

where α_p and $\alpha_{p,i}$ are the thermal expansion coefficients of the mixture and pure component i, respectively; φ_i stands for the volume fraction of component i, φ_i:

$$\varphi_i = \frac{x_i V_i^*}{\sum_{i=1}^{2} x_i V_i^*} \tag{3}$$

where x_i stands for the mole fraction of i component and V_i^* for the molar volume of pure i compound. For the case of viscosity, as ideal state reference term cannot be defined and thus excess property could not be considered, the so-called mixing viscosity, $\Delta\eta$, was calculated:

$$\Delta\eta = \eta - \sum_{i=1}^{2} x_i \eta_i \tag{4}$$

Additionally, excess and mixing properties were fitted to an isothermal Redlich–Kister type polynomial equation as a function of mole fraction, x:

$$Y^E = x(1-x)\sum_{i=0}^{n} A_i (1-2x)^i \tag{5}$$

Likewise, the partial molar volume $\overline{V}_i$ of each i - component was calculated:

$$\overline{V}_i = V_m^E + V_i^* + (1-x_i)\left(\frac{\partial V^E}{\partial x_i}\right)_{T,P} \tag{6}$$

where V_i^* stands for the molar volume of pure component i, with the partial derivative of excess volume calculated from the corresponding Redlich-Kister coefficients. Excess partial molar volumes, $\overline{V}_i^E$, were defined as:

$$\overline{V}_i^E = \overline{V}_i - V_i^* \tag{7}$$

The partial molar volumes at infinite dilution, $\overline{V}_i^{E,\infty}$ were calculated:

$$\overline{V}_i^{E,\infty} = \overline{V}_i^{\infty} - V_i^* \tag{8}$$

where the partial molar volume at infinite dilution, $\overline{V}_i^{\infty}$, is defined as the corresponding limiting value when $x_i \rightarrow 0$.

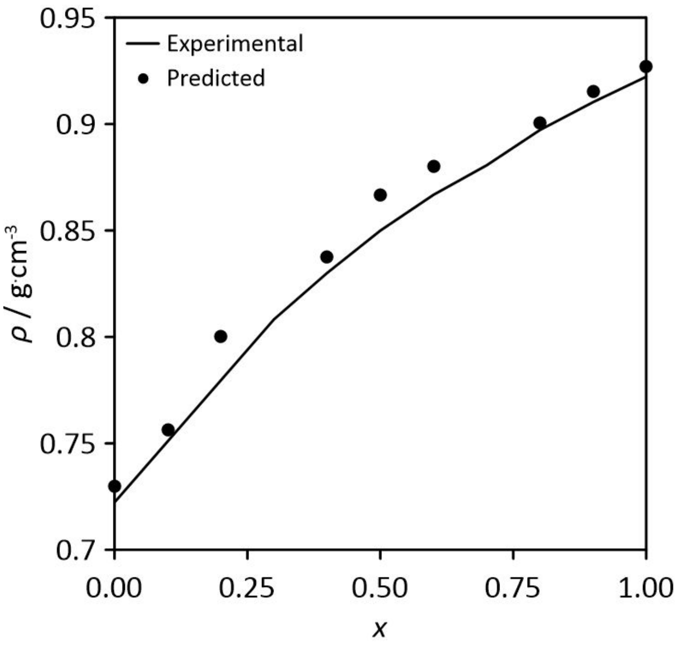

Figure S1. Experimental (line) and MD predicted (dots) density, ρ, for *x* CAR : C10AC (1 : 1) + (1 – *x*) C10 mixtures.

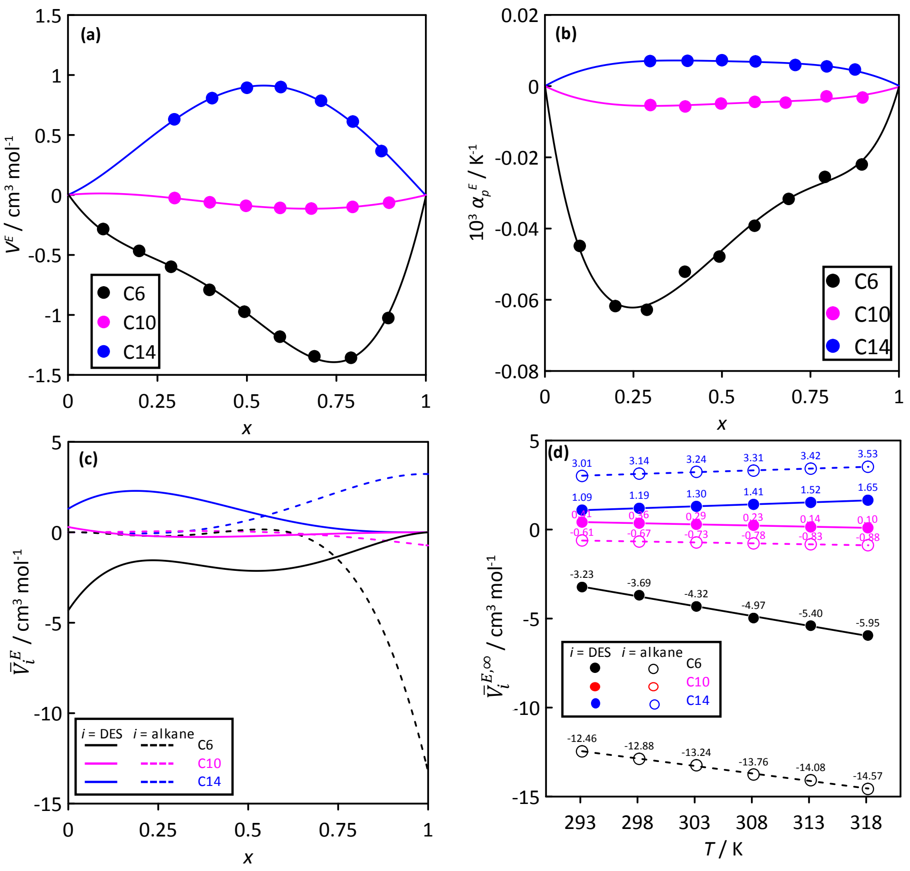

Figure S2. Excess molar volume, V^E, excess thermal expansion coefficient, α_p^E, excess partial molar volume, $\bar{V}_i^E$, for DES and alkane, and excess partial molar volume at infinite dilution, $\bar{V}_i^{E,\infty}$, for HNADES and alkane, for *x* CAR : C10AC (1 : 1) + (1 – *x*) alkane (C6, C10 or C14) mixtures at 303.15 K.

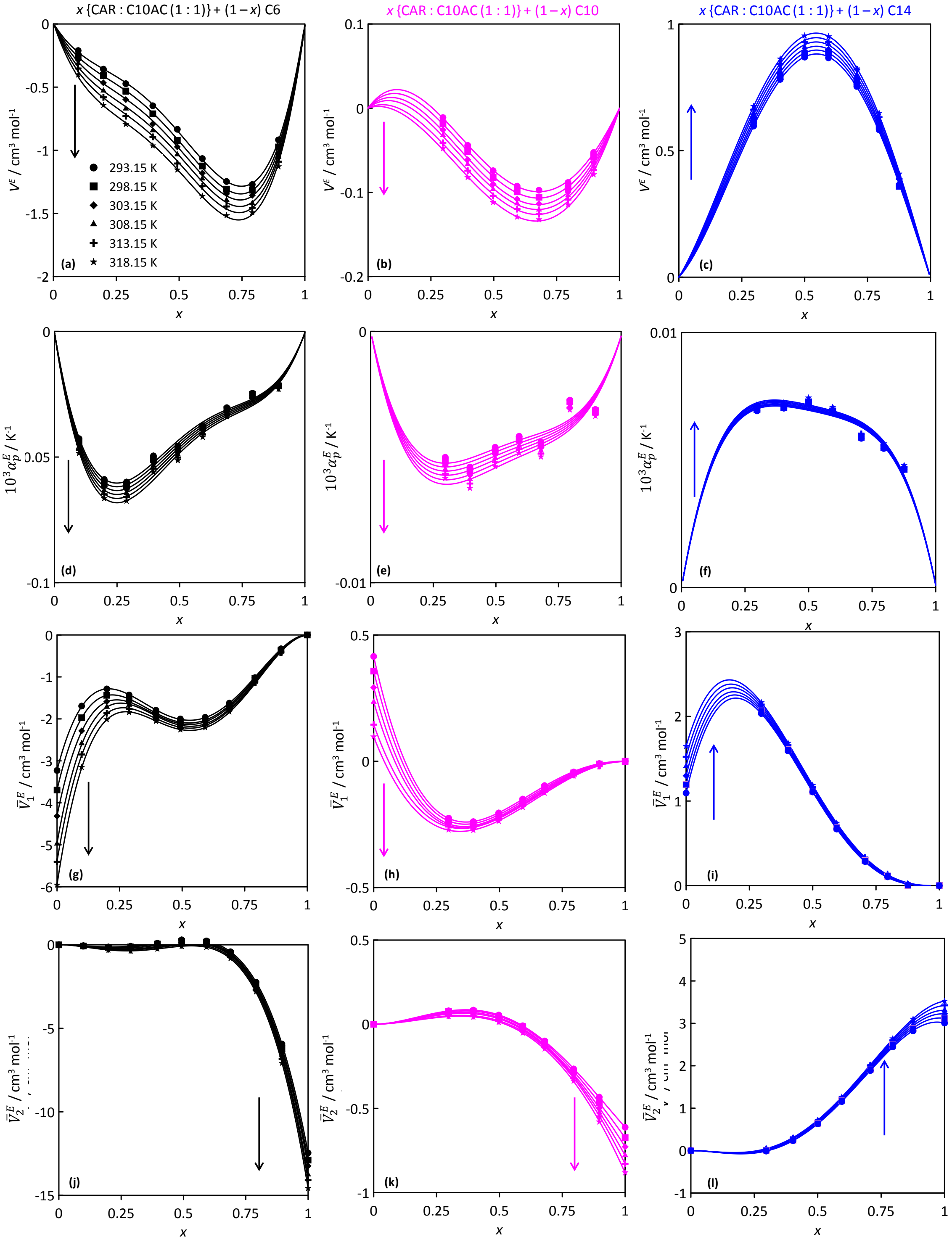

Figure S3. Excess molar volume, V^E, excess thermal expansion coefficient, α_p^E, excess partial molar volume for HNADES, $\overline{V}_1^E$, and alkane, $\overline{V}_2^E$, for the reported mixtures as a function of temperature. Arrows indicate increasing temperature for guiding purposes.

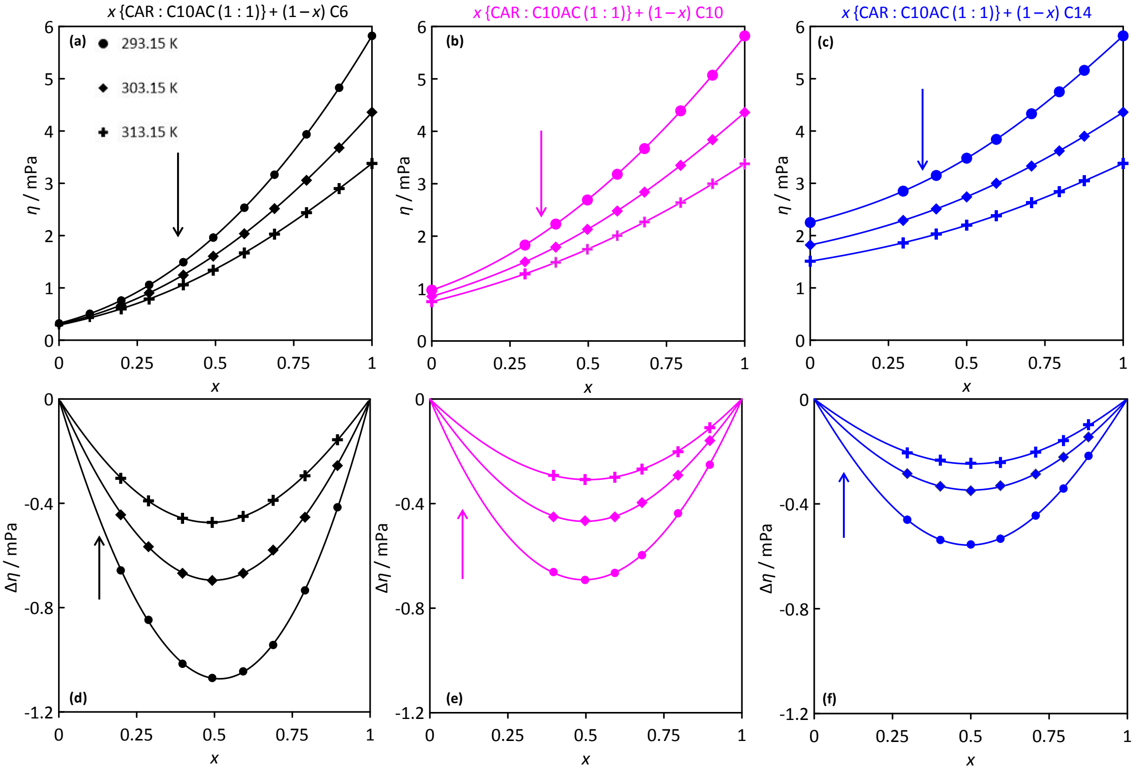

Figure S4. Dynamic viscosity, η, and mixing dynamic viscosity, $\Delta\eta$, for the reported mixtures as a function of temperature. Arrows indicate increasing temperature for guiding purposes.

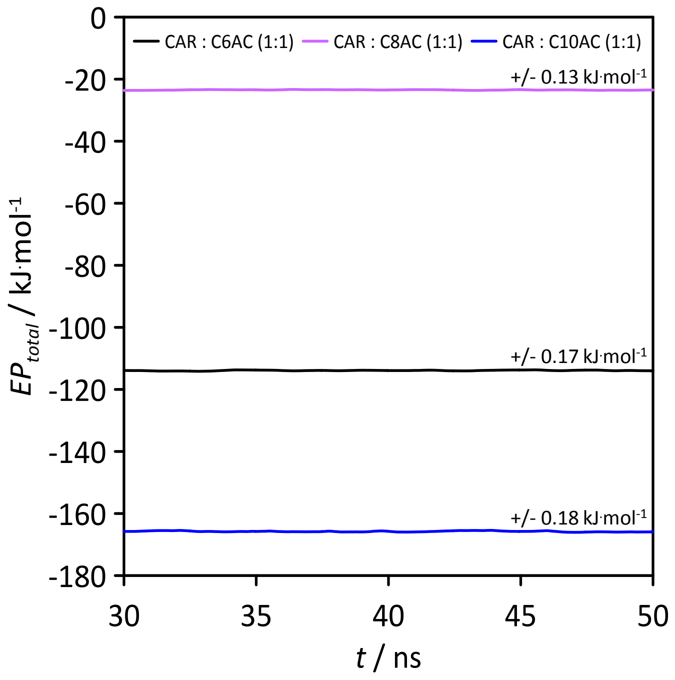

Figure S5. Potential energy, in kJ·mol^{-1}, of the last 20 ns of simulation time to check the equilibration of the systems. Color code: CAR : C6AC (1:1) black; CAR : C8AC (1:1) light purple; CAR : C10AC (1:1) blue.

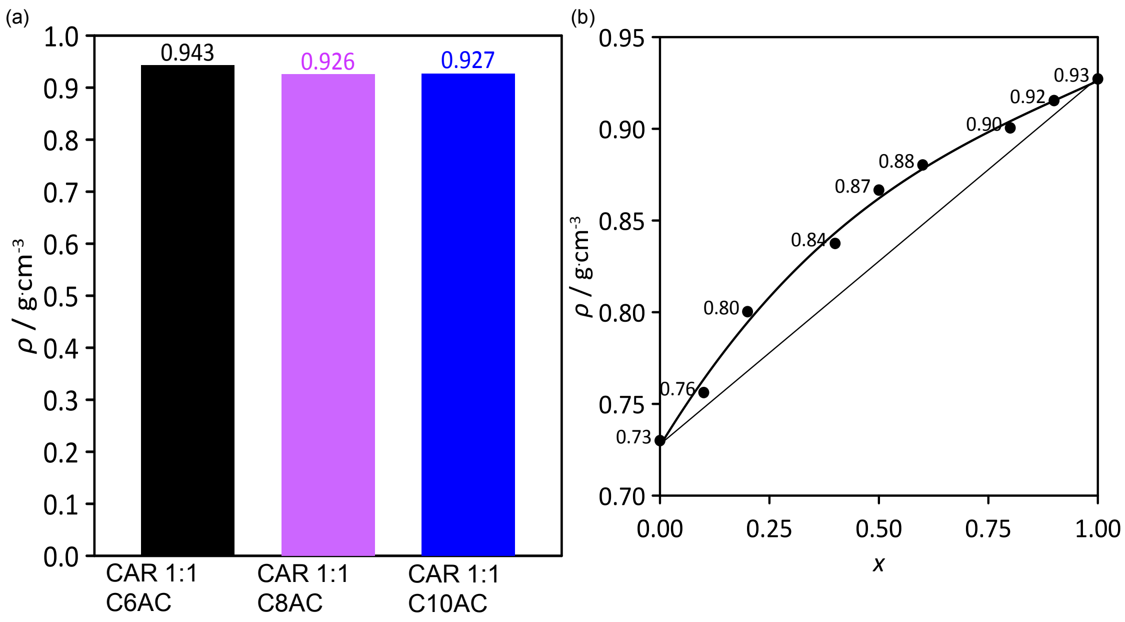

Figure S6. MD predicted density for (a) neat HDESs and (b) *x* CAR : C10AC (1 : 1) + (1 – *x*) C10 mixtures. The straight line in panel (b) represents the ideal mixture.

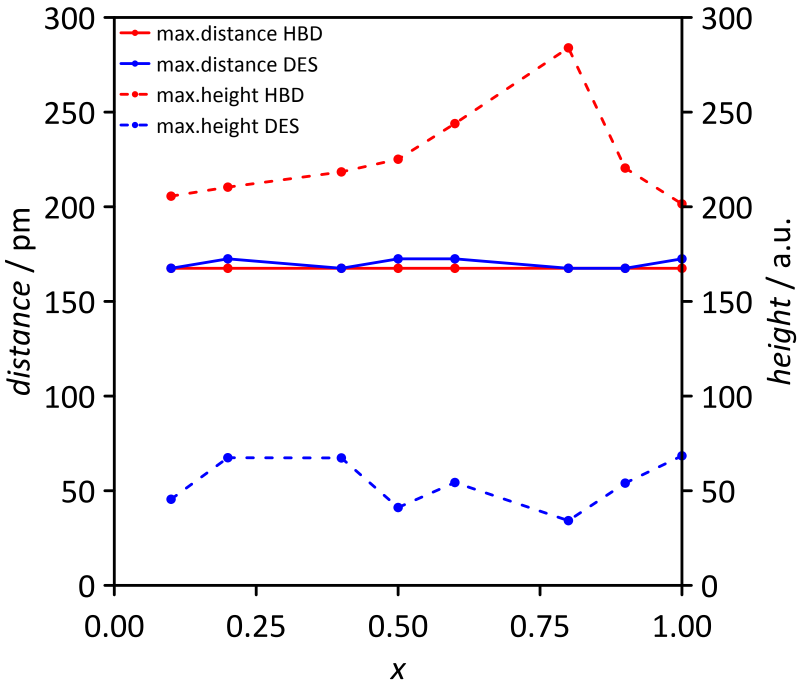

Figure S7. Maximum distance (straight line) and height (dotted line) values extracted from *cmat* functions to characterised the hydrogen bond between CAR as HBA and C10AC as HBD (labelled as DES, color blue) and between C10AC as HBA and C10AC as HBD (labelled HBD, color red) for *x* CAR : C10AC (1:1) + (1-*x*) C10 mixtures.

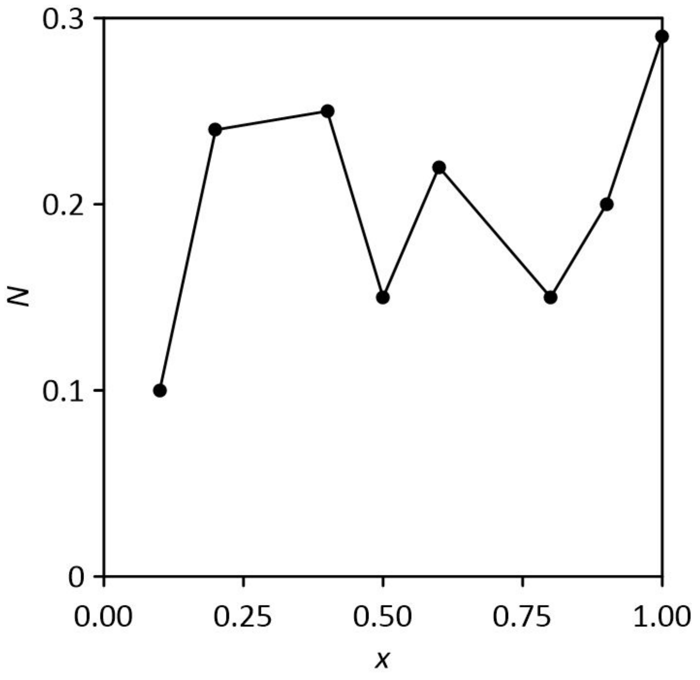

Figure S8. Integrals, *N*, for the first solvation shell, defined for the first minimum in the corresponding RDF for the HNADES-forming hydrogen bond (CAR as HBA and C10AC as HBD) for all the *x* CAR : C10AC (1:1) + (1-*x*) C10 mixtures.
